# Attenuation and Structural Transformation of Crassicauline A During Sand Frying Process and Antiarrhythmic Effects of its Transformed Products

**DOI:** 10.3389/fphar.2021.734671

**Published:** 2021-11-02

**Authors:** Pei Tao, Yan Wang, Yujie Wang

**Affiliations:** ^1^ School of Pharmacy, Chengdu University of Traditional Chinese Medicine, Chengdu, China; ^2^ School of Ethnic Medicine, Chengdu University of Traditional Chinese Medicine, Chengdu, China

**Keywords:** crassicauline A, processing, sand frying, antiarrhythmic, cardiotoxicity

## Abstract

To ensure safety and efficacy, most *Aconitum* herbs should be processed before clinical application. The processing methods include boiling, steaming, and sand frying. Among these methods, the transformation pathways of diterpenoid alkaloids in the process of sand frying are more complicated. Therefore, crassicauline A, a natural product with two ester bonds, was chosen as the experimental object. Consequently, a known alkaloid, together with three new alkaloids, was derived from crassicauline A. Meanwhile, the cardiotoxicity of converted products was reduced compared with their parent compound. Interestingly, some diterpenoid alkaloids have similar structures but opposite effects, such as arrhythmia and antiarrhythmic. Considering the converted products are structural analogues of crassicauline A, herein, the antiarrhythmic activity of the transformed products was further investigated. In a rat aconitine-induced arrhythmia assay, the three transformed products, which could dose-dependently delay the ventricular premature beat (VPB) incubation period, reduce the incidence of ventricular tachycardia (VT), combined with the increasing arrhythmia inhibition rate, exhibited prominent antiarrhythmic activities. Our experiments speculated that there might be at least two transformation pathways of crassicauline A during sand frying. The structure-activity data established in this paper constructs the critical pharmacophore of diterpenoid alkaloids as antiarrhythmic agents, which could be helpful in searching for the potential drugs that are equal or more active and with lower toxicity, than currently clinical used antiarrhythmic drugs.

## Introduction

*Aconitum* L. (Ranunculaceae) is a large herbal medicinal genus, which has 300 species widely distributed in the temperate regions of the northern hemisphere. In China, over 200 species are distributed mainly in western Sichuan, northwestern Yunnan, and eastern Tibet Autonomous Region. Among which 76 species can be extensively employed in traditional Chinese medicine and ethnomedicine (The Flora Committee of Chinese Academy of Sciences, 1979; Xiao et al., 2006). For example, *Aconitum carmichaelii* Debx. and *A. kusnezoffii* Reichb. are commonly used in traditional Chinese medicine (Chinese Pharmacopoeia Commission, 2020), while *A. pendulum* Busch and *A. vilmorinianum* Kom. are usually used in Tibetan medicine and Qiang medicine. They have been employed for the clinical treatment of pains, rheumatics, and neurological disorders (Wang et al., 2007; Wang et al., 2009; Li et al., 2017).

Diterpenoid alkaloids, as the bioactive compounds of *Aconitum* herbs, exert anti-inflammatory and analgesic activities, but also have the drawbacks of cardiotoxicity and neurotoxicity (Chen and Zheng, 1987; Wu et al., 2018). For example, aconitine, a famous diester diterpenoid alkaloid (DDA), can specifically bind with the voltage-sensitive sodium channels on the cell membranes of cardiomyocytes and nerve cells, causing a persistent activation of the sodium channels (Ameri, 1998; Coulson et al., 2017), and continuing sodium influx, destroying the homeostasis of the internal environment, resulting in numbness of limbs, respiratory depression, and arrhythmia (Chan, 2009). In severe cases, it can lead to multiple organ dysfunction and even death.

It must be noted that, in the clinical practice of traditional Chinese medicine and ethnomedicine, most *Aconitum* herbs should be processed with conventional methods to reduce the toxicity, such as boiling, steaming, and sand frying. The boiling (boiling with water for 4∼6 h) and steaming (steaming for 6∼8 h) methods are diffusely applied in traditional Chinese medicine, of which the structural transformation pathways of DDAs have been elucidated clearly, mainly because DDAs are successively hydrolyzed into less toxic monoester- and amines-diterpenoid alkaloids (Gong, 2005). The method of sand frying is a unique processing method for *Aconitum* herbs in ethnomedicine, which requires only 10–15 min of stir-frying with sand to achieve toxicity reduction. However, the transformation pathways of DDAs are more complicated in the sand frying process, and the transformation mechanism is not clear by far.

The processing mechanisms for different processing methods (boiling, steaming, and sand frying) of *A. pendulum* Busch were investigated in our previous studies (Wang et al., 2010a; Wang et al., 2010b; Wang et al., 2011). It was confirmed that aconitine, deoxyaconitine, and 3-acetylaconitine, three main ingredients of *A. pendulum* Busch, were converted into the corresponding monoester- or amines-diterpenoid alkaloids by hydrolyzing the ester bonds at the C-8 and C-14 position during the boiling and steaming process (Wang et al., 2010a; Wang et al., 2010b). However, 16-epi-pyroaconitine and 16-epi-pyrodeoxyaconitine, the corresponding products of aconitine and deoxyaconitine, have been isolated from the sand-fried processed *A. pendulum* Busch (Wang et al., 2010b; Wang et al., 2011). It was found that, by comparing the chemical characteristics of the above four compounds, the substituents at C-15 of aconitine and deoxyaconitine, which were hydroxyl groups, were converted into carbonyl groups after processing. It is speculated that the prototype alkaloids with different substituents at the C-15 position may have different structural transformation pathways.

Crassicauline A (bulleyaconitine A), the main component of many *Aconitum* drugs produced in Yunnan province (Xiao et al., 2006), such as *Aconitum hemsleyanum* E. Pritz. (syn. *A. crassicaule* W. T. Wang) (Wang and Fang, 1981), is a commonly used analgesic in China, whose structural analogues have more potential to be developed as drugs. Besides, crassicauline A does not bear a hydroxyl group at C-15, making it less susceptible to oxidation during the sand frying process, which may have different transformation pathways from aconitine and deoxyaconitine during processing (Wang et al., 2010b; Wang et al., 2011). Therefore, crassicauline A was chosen as the experimental object, and the oil bath heating was used to simulate the sand frying process. Simultaneously, the temperature and time parameters for the structural transformation of crassicauline A were screened via high-performance liquid chromatography (HPLC) method. Moreover, the corresponding processed products of crassicauline A were obtained by processing based on screened parameters. The transformed components were further separated and identified. By comparing the structure changes and toxicity between prototype compound and converted products, the structural transformation pathways of crassicauline A were clarified, as well as whether the sand frying method could attenuate the toxicity.

Notably, some diterpenoid alkaloids exhibit opposite effects, though their structures are extremely similar. For example, aconitine has serious proarrhythmic effects, while aconitine-like compounds such as 14-benzoyltalatisamine, 14-benzoyldelcosine, and 14-benzoyldictyocarpine (Lin et al., 2004; Wang, 2009) have antiarrhythmic effects. Considering crassicauline A is structurally similar to its transformed products. It was unclear, with the transformation of structure, whether the transformed products have antiarrhythmic effects on the premise of reduced toxicity. This intriguing problem beckoned for an explicit solution, to clarify this question, we were engaged in relative experiments to elucidate above speculation.

## Materials and Methods

### General Experimental Procedures

Shimadzu LC-2030C system (Shimadzu Corporation, Japan) and LabSolutions software program were used for chromatographic separation of components and analyzing HPLC data. Preparative-HPLC (Gelaipu Corporation, Chengdu, China), equipping with a GL6000-cp1000 high-pressure infusion pump, GL6000-UV3292 ultraviolet detector, and GelaiInst software program. The packing used in the pre-HPLC column (200 × 100 mm, 10 μm) was purchased from Acchrom Co., Ltd. (Wenling, China). BL-420F Organism Functional Experimental System (Chengdu Techman Co., Ltd. Chengdu, China) was used to record the lead II electrocardiograms (ECGs). CPA2250 electronic analytical balance (Sartorius, Germany) was used to correctly weigh the lab samples. The process of sand frying was simulated by oil bath heating using an HH-SJ heat-collecting magnetic stirrer (Jintan Chengdong Xinrui Instrument Factory, Changzhou, China). Optical rotations were obtained on a Perkin-Elmer 341 polarimeter (PerkinElmer, United States). Melting points were measured on an X-4 micro-melting point apparatus and uncorrected (Shanghai Precision Scientific Instrument Corporation, Shanghai, China). NMR spectra were measured on a Bruker Avance 600 spectrometer (Bruker, Germany) in CDCl_3_ with tetramethylsilane (TMS) as internal standard. Mass spectra were carried out on a micrO-TOF-Q-II mass spectrometer (Bruker, Germany). RE-3000B rotary evaporator (Shanghai Yarong Biochemical Instrument Factory, Shanghai, China) was used to evaporate the solvent. Silica gel G (200–300 mesh) for column chromatography and TLC plates (silica gel G) were obtained from Qingdao Sea Chemical Factory (Qingdao, China).

### Chemicals and Reagents

Crassicauline A (purity ≥98%) was purchased from Chengdu Desite Bio-Technology Co., Ltd. (Chengdu, China), aconitine (purity = 99%) from Shaanxi Herbchem Bio-Technology Co., Ltd. (Xi’an, China). Propafenone hydrochloride (purity = 99.8%) and lidocaine hydrochloride (purity = 93.4%) were purchased from National Institutes for Food and Drug Control (Beijing, China), urethane from Chengdu Kelong Chemical Reagent Factory (Chengdu, China). Compounds **1**–**4** (purity ≥95%) were separated and identified from processed crassicauline A, the structures were verified by ^1^H NMR, ^13^C NMR, and HR-ESI-MS.

HPLC-grade acetonitrile was supplied by Fisher Chemical Co. (New Jersey, United States). Ultrapure water was directly obtained from a ULUP-I-10T water purification system (Chengdu, China). The remaining reagents were all of analytical grade. Spots on TLC plates were visualized with Dragendorff’s reagent or iodine vapor.

### Animals

SPF grade Sprague Dawley rats (male and female) weighing between 180 and 220 g [certificate No. SCXK (CHUAN) 2015-030 and 2020-030] were supplied from Chengdu Dossy experimental animals Co., Ltd. (Chengdu, China) and maintained under a controlled light/dark cycle and temperature (20 ± 2°C), with free access to food and water. They were left for 2 days for acclimatization to animal room conditions. The animal study was reviewed and approved by the Animal Experimentation Ethics Committee of Chengdu University of Traditional Chinese Medicine (permission No. 2020-15).

### Screening of Processing Parameters

#### Chromatographic Conditions

Quantitative analyses were performed with a Shimadzu LC-2030C system (Shimadzu Corporation, Japan), using a Supersil ODS-B C_18_ column (250 × 4.6 mm, 5 μm-Elite, China) maintained at 40°C. The detection wavelength was 260 nm. The mobile phase comprised acetonitrile (A) and 0.03 moL/L NH_4_HCO_3_ (The aqueous was alkalized to pH 9.5 using 25% NH_4_OH, B) with a gradient program of 37% A at 0–40 min, 37–70% A at 40–60 min, and 70% A at 60–85 min. The injection volume was 10 μl and the flow rate was 1 ml/min.

### Preparation of Standard Solution

100.60 mg crassicauline A standard was weighed accurately into a 250 ml volumetric flask and dissolved in dichloromethane to make a concentration of 402.4 μg/ml reaction stock solution. 4 ml of the reaction stock solution was added into a 10 ml volumetric flask, with dichloromethane evaporating, 0.1% (v/v) HCl-methanol was added for dissolution to prepare a final concentration of 160.96 μg/ml. The standard solution was filtered with 0.45 μm syringe filter before injection into the HPLC.

### Preparation of Processed Product Sample Solution

Twenty-four 100 ml round-bottomed flasks were divided into four temperature groups (120°C, 140°C, 160°C, and 180°C), six reaction time points (1, 5, 10, 20, 30, and 40 min) were set at each temperature. Accurately adding 4 ml of the reaction stock solution into each flask, and the solvent was evaporated. The flasks were subsequently immersed in an oil bath and processing according to set parameters, cooling to room temperature after reaction. The residue was diluted with 0.1% (v/v) HCl-methanol in a 10 ml volumetric flask and subsequently filtered with 0.45 μm syringe filter before injection into the HPLC.

#### Preparation and Separation of Compounds 1-3

1.83 g crassicauline A was dissolved in a 250 ml round-bottomed flask with a proper amount of dichloromethane, and the solvent was evaporated with a rotary evaporator at 40°C to make crassicauline A uniformly adhere to the inner wall of the flask, and processed in an oil bath at 160°C for 30 min, obtaining a crude residue of crassicauline A (1.65 g) for column chromatography.

The residue obtained was purified over silica gel column chromatography (120 g, 200–300 mesh) and eluted with petroleum ether-acetone-triethylamine 8:1:0.01 (0.4 L), 6:1:0.01 (3 L), and 3:1:0.01 (1.2 L) to furnish three fractions (A-C). Fraction B (1 g) was further separated by preparative TLC on silica gel G (petroleum ether-acetone-triethylamine, 3:3:0.01) to obtain Frs T_1_ (R*f* 0.41), Frs T_2_ (R*f* 0.51), and Frs T_3_ (R*f* 0.77). Frs T_3_ was recrystallized from dichloromethane-methanol to generate compound **3** (100 mg). Frs T_1_ (200 mg) and Frs T_2_ (430 mg) were further purified by preparative HPLC, with the chromatographic condition of acetonitrile −0.03 moL/L NH_4_HCO_3_ at 42:58 (The aqueous was basified to pH 9.5 using 25% NH_4_OH, and the detection wavelength was 260 nm), the collected fractions were evaporated under reduced pressure to no acetonitrile, leaving only the aqueous phase. Following this, the aqueous phase was extracted twice with dichloromethane. The combined extracts were subsequently dried over Na_2_SO_4_, the solvent was removed in vacuo to obtain compounds **1** (110 mg) and **2** (310 mg).

#### Preparation and Separation of Compound 4

1.11 g crassicauline A was weighed and processed upon above-mentioned processing method, the processing parameters were set at 180°C for 30 min, to obtain a crude residue of crassicauline A (1 g) for column chromatography.

The residue was subjected to column chromatography (silica gel, 120 g, 200–300 mesh) and eluted with dichloromethane-acetone-methanol-triethylamine 60:2:1:0.01 (5.7 L) and 10:2:1:0.01 (0.8 L) to obtain three fractions (D-F). Fraction D (400 mg) was further separated by preparative TLC on silica gel G (petroleum ether-acetone-triethylamine, 2:1:0.01) to generate fraction T_4_ (R*f* 0.55), which was recrystallized from petroleum ether-acetone-methanol to afford compound **4** (300 mg).

### Electrocardiography

The rats were anesthetized by intraperitoneal (*i.p.*) injection of 20% urethane (1.2 g/kg), with their back fixed and four limbs in subcutaneous penetration of needle-electrodes. Lead II ECGs were recorded after the administration of urethane.

### Cardiotoxicity Test

**FIGURE 1:**
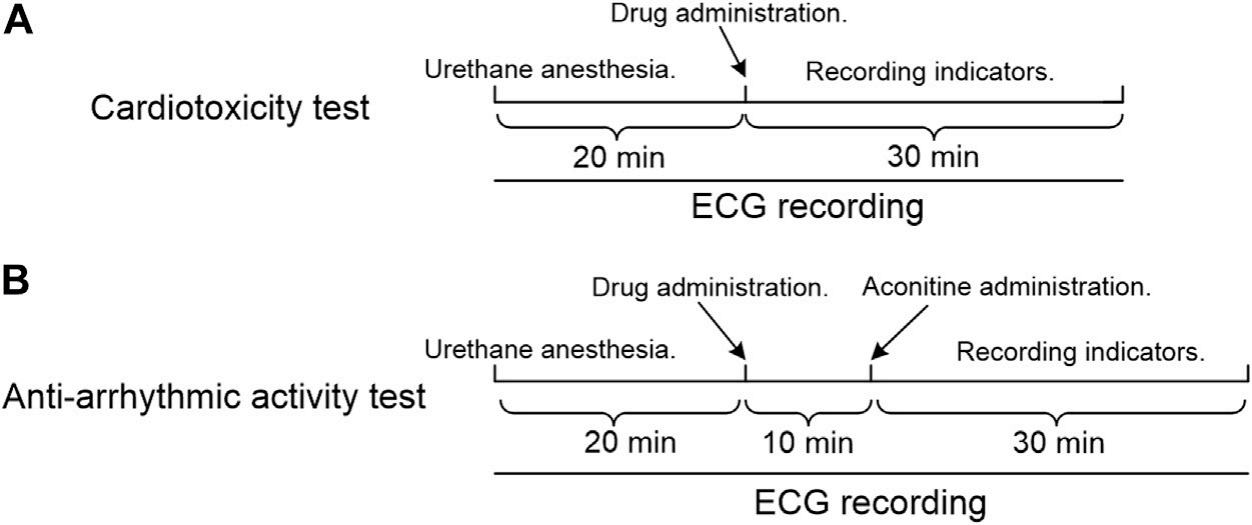
Experimental timeline. **(A)** Cardiotoxicity test; **(B)** Anti-arrhythmic activity test.

*In vivo* studies were carried out to assess the cardiotoxicity of crassicauline A and its converted products. 40 rats were equally divided into four groups, five female and five male rats for each group were intravenously injected with the same dose of compounds. Lead II ECGs were recorded for 20 min prior to drug administration, recording the ECG changes within 30 min after administration (Figure 1).

Through the preliminary experiment, it was found that 0.10 mg/kg crassicauline A could cause ventricular premature beat (VPB), ventricular tachycardia (VT), and ventricular fibrillation (VF) in rats. Comparing under the same dose, whether the converted products caused arrhythmias can directly reflect the cardiotoxicity pre- and post-processing.

### Aconitine-Induced Arrhythmia Test

To further investigate the antiarrhythmic activity of the three converted alkaloids, the testing was performed *in vivo* in 233 rats. The rats were randomly divided into the following 15 groups, a minimum of ten animals were used in each group: the blank solvent group (Take 4 ml of 1% HCl-ethanol solution, dilute it with saline, add 5% NaOH to adjust pH 7, and fixed volume with saline to 100 ml), the control group (Aconitine, 0.03 mg/kg body weight), the positive control groups (Lidocaine, 5.0 mg/kg (Hong et al., 2019) body weight; propafenone, 3.2 mg/kg (Li et al., 2006) body weight), the compound **1** groups (0.05, 0.20, 0.40, and 0.60 mg/kg body weight), the compound **2** groups (0.05, 0.20, 0.30, and 0.40 mg/kg body weight), and the compound **4** groups (0.05, 0.20, and 0.40 mg/kg body weight). The preparation methods of aconitine, positive drugs, and the experimental compounds were the same as the blank solvent. To confirm whether the blank solvent had an influence on rats, the rats of solvent group were only administered the same volume of blank solvent to observe ECG changes during the recording time.

The rats were anesthetized using 20% urethane (1.2 g/kg, *i.p.*) (Bai et al., 2012; Mohamed et al., 2016). Recording the lead II ECGs for 20 min prior to administration, the experimental compounds, positive drugs, and equal volume of saline were subsequently administered *via* the exposed vena femoral, respectively. After stabilization for 10 min (Zhang et al., 2006), aconitine was administered into the uncovered vena femoral at a dosage of 0.03 mg/kg (Bartosová et al., 2005; Klekot, 2006; Bartosova et al., 2007) to establish arrhythmia (Figure 1). The onset time of VPB (Qiu et al., 2016) was recorded within 30 min (Sun et al., 2006; Zhang and Xiong, 2015; Qiu et al., 2016) after aconitine. Meanwhile, the VT (Meng et al., 1992; Ma et al., 2018) or arrhythmia (Wang et al., 1997a; Wang et al., 1997b), if any, was recorded for each group at the end of the observation period.

### Statistical Analysis

The data obtained were analysed using statistical package for social sciences (SPSS) version 20 software. Experimental data were expressed as mean ± SD or proportion. Descriptive statistics were examined individually. When the data conformed to the normal distribution, the one-way ANOVA would be used for those with homogeneous variance. If the variance was not homogeneous, Tamhane’s T2 test would be used for comparison between groups. Comparisons of proportions were made with the Pearson’s chi-square (*χ* ^2^) test. Statistical significance was set at *p* < 0.05.

## Results

### Method Validation

Analysis of the peak area of the standard sample resulted from HPLC as well as linear regression (*Y* = 21239X-3038.8, *r* = 0.9996), the obtained results showed that crassicauline A exhibited satisfactory linearity within the range of 2–200 μg/ml. The LOD and LOQ of crassicauline A under the chromatographic conditions were 0.18 μg/ml and 0.60 μg/ml, respectively.

Precision, stability, and repeatability of the method were additionally verified. During the determination, the RSD values of crassicauline A for the precision, stability, and repeatability were 1.29, 2.33, and 2.69%, respectively, indicating that the established method was precise, stable, and accurate enough for determination.

### Temperature and Time for the Structural Transformation of Crassicauline A

**FIGURE 2:**
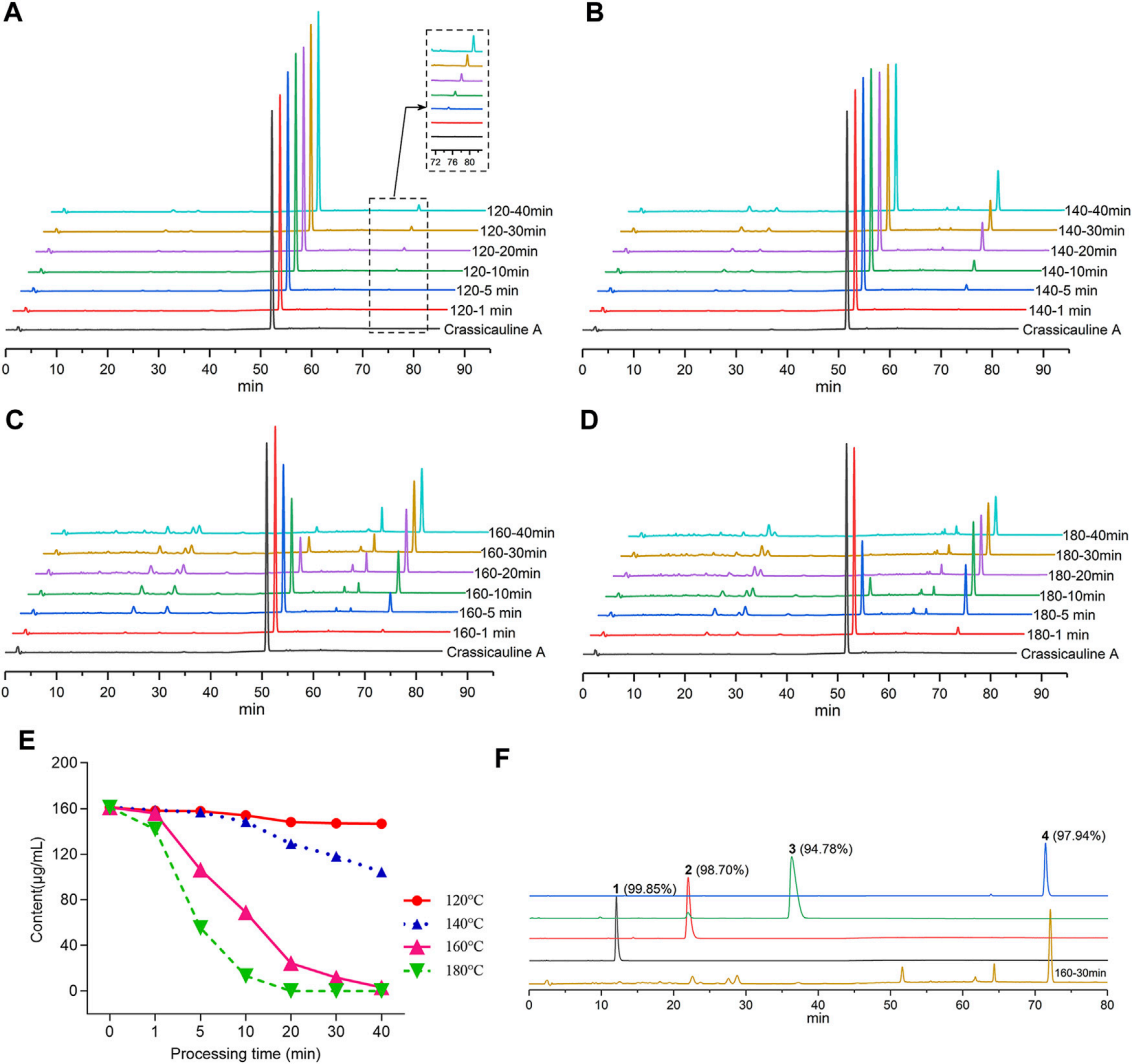
Contents for crassicauline A of different processed products and purity test. **(A–D)** HPLC chromatograms of different carssicauline A processed products. (A: 120°C, B: 140°C, C: 160°C, D: 180 °C). **(E)** Content variety of crassicauline A under different processing temperatures and time. **(F)** Purity of the transformed products.

To screen out the temperature and time range for the structural transformation of crassicauline A in the sand frying process, the sample solutions, acquired under different processing temperatures and time, along with the standard solutions, were injected into the HPLC, respectively. The chromatograms were obtained after determination, which could visually reflect the dynamic change of crassicauline A. The chromatograms are shown in Figures 2A–D.

In light of the above experimental findings (Figures 2A–E), it could be found that the content of crassicauline A before processing was 160.96 μg/ml. When crassicauline A was processed at 140–180°C, with the elevation of temperature and prolongation of processing time, the content of crassicauline A decreased. For instance, the content of crassicauline A was 69.07 μg/ml after being processed for 10 min at 160°C and continuously dropped to 24.42 μg/ml for 20 min. When processed at 180°C for 5 min, the content decreased to 55.47 μg/ml, even declined to 0 μg/ml for another 15 min. Meanwhile, some unknown chromatographic peaks emerged in chromatograms. As a result, the quantity and content of the above transformed products were increased obviously when processed at 160°C for 30 min. Therefore, in this paper, the processed products were prepared based on above processing parameters.

### Structural Identification of Converted Products

Compounds **1**-**4** were subsequently isolated from processed crassicauline A by procedures described in the experimental section, and the purity of converted products was determined by re-injected into the HPLC (Figure 2F).

**FIGURE 3:**
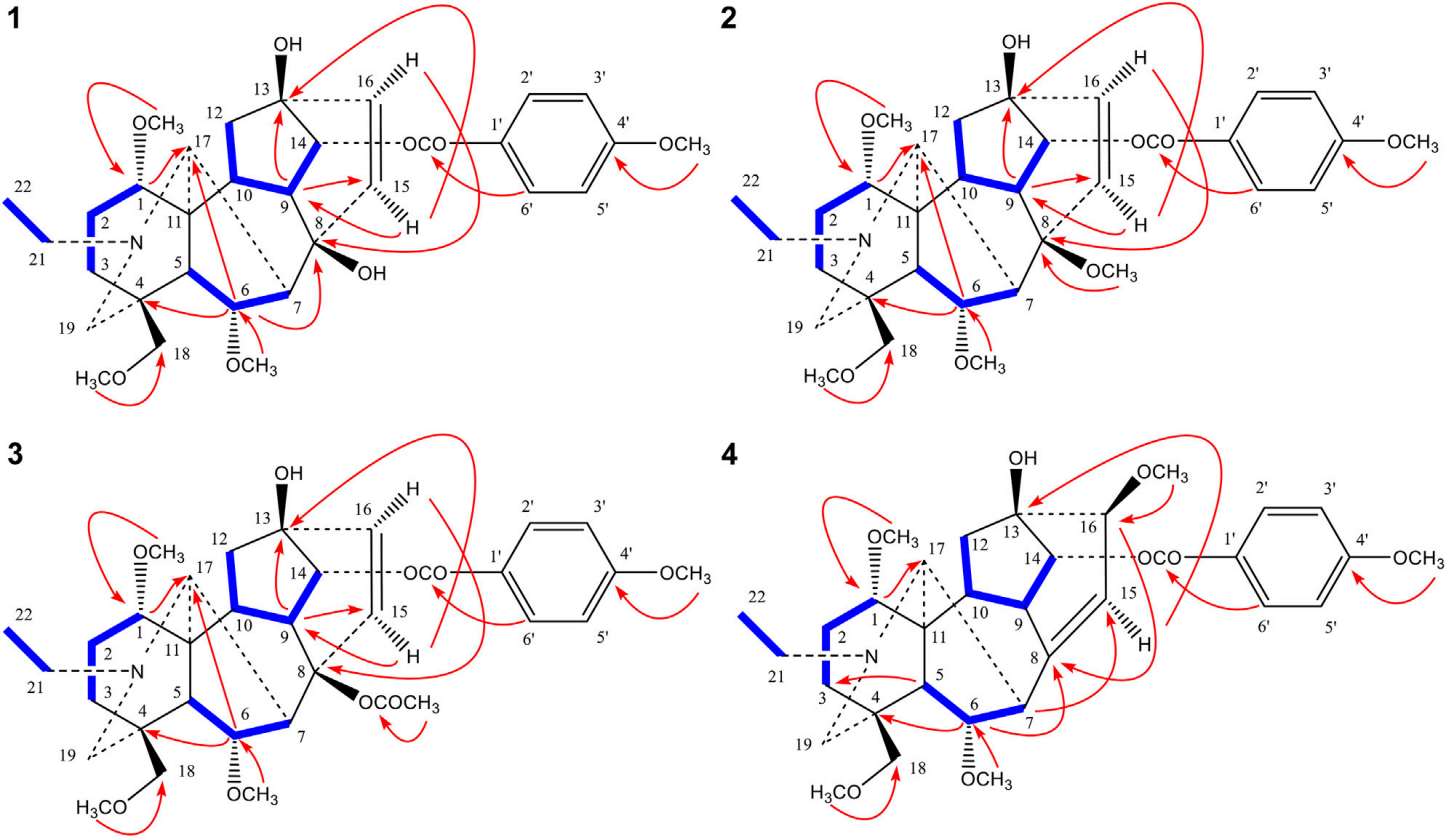
Key ^1^H-^1^H COSY (▬) and HMBC (H→C) correlations of compounds **1**–**4**.

**FIGURE 4:**
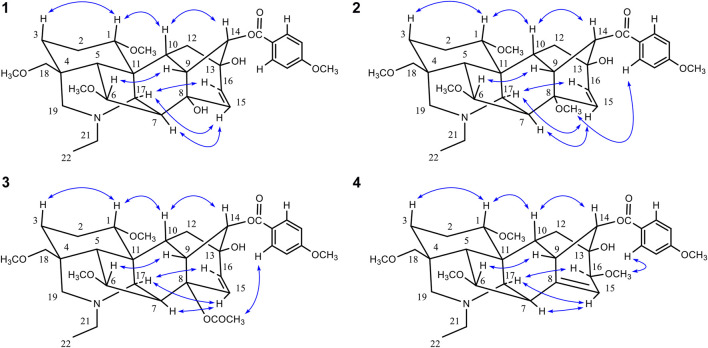
Key NOE correlations (H↔H) of compounds **1**–**4**.

16-demethoxy-∆^15(16)^-8-deacetylcrassicauline A (**1**) was isolated as an amorphous powder, ${\left[\text{α}\right]}_{\text{D}}^{20}$ + 47.1°(*c* = 0.92, CH_3_OH). It showed a positive reaction with Dragendorff’s reagent. Its molecular formula was deduced to be C_32_H_43_NO_8_ from a protonated molecule ion at *m/z* 570.3052 (M + H)^+^ (calcd. 570.3061) in its HR-ESI-MS. The NMR spectra (Table 1) of compound **1** showed the presence of an *N*-ethyl group (*δ*_H_ 1.06, 3H, t, *J* = 7.32 Hz; *δ*_H_ 2.51, 2.59, each 1H, m; *δ*_C_ 13.3 q, 49.3 t), three methoxyl groups (*δ*_H_ 3.24, 3.30, 3.32, each 3H, s; *δ*_C_ 56.2 q, 57.6 q, 59.2 q), one *p*-methoxyl benzoyloxy group (*δ*_H_ 6.92, 7.90, each 2H, AA’BB’ system, *J* = 8.82 Hz; 3.85, 3H, s; *δ*_C_ 167.1 s, 121.6 s, 131.7 d (2C), 114.0 d (2C), 163.9 s, 55.5 q), as well as four quaternary carbons (*δ*_C_ 39.4, 50.2, 73.9, 76.9). The aforementioned NMR features suggested an aconitine-type alkaloid for compound **1** (Pelletier et al., 1984). The ^1^H-doublet signal at *δ*_H_ 5.17 (*J* = 5.1 Hz) was assigned to H-14β (Pelletier and Joshi, 1991; Gao et al., 2006), resulting in location of the *p*-methoxyl benzoyloxy group to C-14. Three methoxyl groups were attributed to C-1, C-6, and C-18 based on the cross-peaks between 1-OCH_3_ (*δ*_H_ 3.24) and C-1, 6-OCH_3_ (*δ*_H_ 3.30) and C-6, 18-OCH_3_ (*δ*_H_ 3.32) and C-18 in its HMBC spectrum (Figure 3). Two hydroxyl groups were assigned to C-8 and C-13 based on the correlations between the C-8 and H-6, H-10, H-14, H-16, H-17, as well as C-13 and H-9, H-15 in the HMBC of **1**. The ^1^H NMR signals at *δ* 5.61 (1H, d, *J* = 9.54 Hz, H-15), *δ* 5.91 (1H, d, *J* = 9.54 Hz, H-16), and the ^13^C NMR signals at *δ* 130.1 and 134.8 indicated the existence of a disubstituted double bond in the molecule (Yue et al., 1994a; Yue et al., 1994b; Desai et al., 1998). Also, long-range correlations between C-13 and H-15, C-8 and H-16 confirmed the presence of a double bond between C-15 and C-16. In addition, the coupling constant between H-15 and H-16 was 9.54 Hz, suggesting that the two hydrogen atoms were CIS structures. The key NOE correlations (Figure 4) between H-15 and H-17, H-16 and H-17 also confirmed the configuration of H-15 and H-16 in **1** was α-orientation. The NMR spectra of compound **1** lacked a methoxyl group at C-16, when compared with crassicauline A (Chen et al., 2002), and the ^13^C NMR spectra of **1** clearly showed changes in the chemical shifts of C-8, C-15, and C-16 due to the absence of an acetoxyl group and the presence of a double bond. Except for these points, the ^13^C NMR spectra of the two alkaloids were very similar. From these deductions, the structure of compound **1** was assigned as 16-demethoxy-∆^15(16)^-8-deacetylcrassicauline A, a new diterpenoid alkaloid (Supplementary Figures S1–S13).

**TABLE 1:** ^1^H (600 MHz) and ^13^C (150 MHz) NMR data for compound **1** (CDCl_3_).

Position	*δ*_H_ (*J* in Hz)	*δ*_C_, type	HMBC	NOESY	^1^H-^1^H COSY
1	3.05 m	85.1, CH	C-10, C-17, 1-OCH_3_	H-3β, H-10, 1-OCH_3_	H-2α, β
2α	2.29 m	25.9, CH_2_	—	—	H-1, H-2β, H-3α, β
2β	1.91 m		—	—	H-1, H-2α, H-3α, β
3α	1.70 m	34.7, CH_2_	—	H-18α, β	H-2α, β, H-3β
3β	1.58 m		C-19	H-1, H-5	H-2α, β, H-3α
4	—	39.4, C	—	—	—
5	2.07 d (6.6)	49.1, CH	C-7, C-10, C-17, C-19	H-3β, H-18β	H-6
6	4.19 d (6.6)	81.7, CH	C-4, C-8, C-17, 6-OCH_3_	H-9, 6-OCH_3_	H-5, H-7
7	2.11 s	49.1, CH	C-11, C-15	H-15, 6-OCH_3_	H-6
8	—	73.9, C	—	—	—
9	2.55 m	47.0, CH	C-12, C-13, C-15	H-6	H-10, H-14
10	2.17 m	42.7, CH	C-8, C-17	H-1, H-14	H-9, H-12α, β
11	—	50.2, C	—	—	—
12α	3.02 m	40.9, CH_2_	C-11, C-14, C-16	—	H-10, H-12β
12β	1.96 m		C-9, C-16	H-14	H-10, H-12α
13	—	76.9, C	—	—	—
14	5.17 d (5.1)	80.3, CH	C-8, C-16, ArC = O	H-10, H-12β	H-9
15	5.61 d (9.54)	130.1, CH	C-9, C-13	H-7, H-17	H-16
16	5.91 d (9.54)	134.8, CH	C-8	H-17	H-15
17	3.07 s	62.6, CH	C-6, C-8, C-10, C-19	H-15, H-16, H-21, H-22	—
18α	3.29 d (8.46)	80.6, CH_2_	C-3, C-5, C-19, 18-OCH_3_	H-3α, H-5, H-19α, β, 18-OCH_3_	H-18β
18β	3.72 d (8.46)		C-3, C-19, 18-OCH_3_	H-5, H-19β, 18-OCH_3_	H-18α
19α	2.58 m	54.2, CH_2_	C-3, C-5, C-17	H-18α	H-19β
19β	2.65 m		C-3	H-18α, β	H-19α
21α	2.51 m	49.3, CH_2_	—	H-17	H-22
21β	2.59 m		—	H-17	H-22
22	1.06 t (7.32)	13.3, CH_3_	—	H-17	H-21α, β
1-OCH_3_	3.24 s	56.2, CH_3_	C-1	H-1	—
6-OCH_3_	3.30 s	57.6, CH_3_	C-6	H-6, H-7	—
18-OCH_3_	3.32 s	59.2, CH_3_	C-18	H-18α, β	—
ArC = O	—	167.1, C	—	—	—
1′	—	121.6, C	—	—	—
2′, 6′	7.90 d (8.82)	131.7, CH	C-4′, ArC = O	—	H-3′, 5′
3′, 5′	6.92 d (8.82)	114.0, CH	C-1′	4′-OCH_3_	H-2′, 6′
4′	—	163.9, C	—	—	—
4′-OCH_3_	3.85 s	55.5, CH_3_	C-4′	H-3′, 5′	—

16-demethoxy-∆^15(16)^-8-*O*-methylcrassicauline A (**2**) was isolated as a white amorphous powder, ${\left[\alpha \right]}_{\text{D}}^{20}$ + 61.5°(*c* = 0.78, CH_3_OH). Its molecular formula C_33_H_45_NO_8_ was derived from the HR-ESI-MS spectral data: *m/z* 584.3203 (M + H)^+^ (cacld. 584.3218). ^1^H- and ^13^C-NMR spectra of **2** (Table 2) showed the distinct NMR features of an aconitine-type C_19_-diterpenoid alkaloid skeleton (Pelletier et al., 1984), bearing an *N*-ethyl group (*δ*_H_ 1.05, 3H, t, *J* = 7.38 Hz; *δ*_H_ 2.49, 2.54, each 1H, m; *δ*_C_ 13.3 q, 49.2 t), four methoxyl groups (*δ*_H_ 3.12, 3.23, 3.26, 3.29, each 3H, s; *δ*_C_ 49.1 q, 56.2 q, 57.8 q, 59.2 q), a disubstituted double bond (*δ*_H_ 5.80, 1H, d, *J* = 9.9 Hz; *δ*_H_ 6.03, 1H, d, *J* = 9.9 Hz), and a *p*-methoxyl benzoyloxy group (*δ*_H_ 6.89, 7.98, each 2H, AA’BB’ system, *J* = 8.82 Hz; 3.84, 3H, s; *δ*_C_ 167.3 s, 122.7 s, 131.9 d (2C), 113.5 d (2C), 163.4 s, 55.4 q). The ^1^H-doublet signal at *δ*_H_ 4.95 (*J* = 4.08 Hz) was assigned to H-14β (Pelletier and Joshi, 1991; Gao et al., 2006), resulting in location of the *p*-methoxyl benzoyloxy group to C-14. Four methoxyl groups were assigned to C-1, C-6, C-8, C-18 due to the long-range correlations between 1-OCH_3_ (*δ*_H_ 3.23) and C-1, 6-OCH_3_ (*δ*_H_ 3.26) and C-6, 8-OCH_3_ (*δ*_H_ 3.12) and C-8, 18-OCH_3_ (*δ*_H_ 3.29) and C-18 in the HMBC spectrum of **2** (Figure 3). A remaining hydroxyl group was assigned to C-13 based on the correlations between the C-13 and H-9, H-15 in the HMBC of **2**. Also, the presence of a double bond between C-15 and C-16 could be corroborated by the HMBC correlations (Figure 3) from H-15 to C-13 and from H-16 to C-8 (Yue et al., 1994a; Yue et al., 1994b; Desai et al., 1998). The NOE correlations (Figure 4) could be observed between H-15 and H-17, H-16 and H-17 in compound **2**. As a result, the configuration of H-15 and H-16 in compound **2** was unambiguously established to have an α-orientation.

**TABLE 2:** ^1^H (600 MHz) and ^13^C (150 MHz) NMR data for compound **2** (CDCl_3_).

Position	*δ*_H_ (*J* in Hz)	*δ*_C_, type	HMBC	NOESY	^1^H-^1^H COSY
1	3.03 m	85.3, CH	C-10, C-17, 1-OCH_3_	H-3β, H-10, 1-OCH_3_	H-2α, β
2α	2.27 m	26.1, CH_2_	—	—	H-1, H-2β, H-3α, β
2β	1.93 m		—	—	H-1, H-2α, H-3α, β
3α	1.70 m	34.7, CH_2_	—	H-18α	H-2α,β, H-3β
3β	1.59 m		C-19	H-1, H-5	H-2α,β, H-3α
4	—	39.3, C	—	—	—
5	2.03 d (6.6)	48.8, CH	C-7, C-10, C-17, C-18, C-19	H-3β, H-18α, β	H-6
6	4.10 d (6.6)	82.1, CH	C-4, C-8, C-17, 6-OCH_3_	H-9, 6-OCH_3_	H-5, H-7
7	2.32 s	44.6, CH	C-9, C-11, C-15	H-15, 6-OCH_3_	H-6
8	—	76.9, C	—	—	—
9	2.63 m	45.7, CH	C-12, C-13, C-15	H-6	H-10, H-14
10	2.15 m	42.6, CH	C-8, C-17	H-1, H-14	H-9, H-12α, β
11	—	50.3, C	—	—	—
12α	2.99 m	40.1, CH_2_	C-11, C-14, C-16	—	H-10, H-12β
12β	1.89 m		C-9, C-11, C-16	H-14	H-10, H-12α
13	—	76.9, C	—	—	—
14	4.95 d (4.08)	78.6, CH	C-8, C-16, ArC = O	H-10, H-12β	H-9
15	5.80 d (9.9)	125.2, CH	C-9, C-13	H-7, H-17, 8-OCH_3_	H-16
16	6.03 d (9.9)	137.2, CH	C-8	H-17	H-15
17	2.96 s	62.4, CH	C-5, C-6, C-8, C-10, C-19	H-15, H-16, H-21, H-22	—
18α	3.29 d (8.76)	80.5, CH_2_	C-3, C-5, C-19, 18-OCH_3_	H-3α, H-5, H-19α, β, 18-OCH_3_	H-18β
18β	3.65 d (8.76)		C-3, C-19, 18-OCH_3_	H-5, H-19β, 18-OCH_3_	H-18α
19α	2.56 m	54.1, CH_2_	C-3, C-5, C-17	H-18α	H-19β
19β	2.62 m		C-3	H-18α,β	H-19α
21α	2.49 m	49.2, CH_2_	—	H-17	H-22
21β	2.54 m		—	H-17	H-22
22	1.05 t (7.38)	13.3, CH_3_	—	H-17	H-21α, β
1-OCH_3_	3.23 s	56.2, CH_3_	C-1	H-1	—
6-OCH_3_	3.26 s	57.8, CH_3_	C-6	H-6, H-7	—
8-OCH_3_	3.12 s	49.1, CH_3_	C-8	H-15, H-2′, 6′	—
18-OCH_3_	3.29 s	59.2, CH_3_	C-18	H-18α, β	—
ArC = O	—	167.3 C	—	—	—
1′	—	122.7, C	—	—	—
2′, 6′	7.98 d (8.82)	131.9, CH	C-4′, ArC = O	8-OCH_3_	H-3′, 5′
3′, 5′	6.89 d (8.82)	113.5, CH	C-1′	4′-OCH_3_	H-2′, 6′
4′	—	163.4, C	—	—	—
4′-OCH_3_	3.84 s	55.4, CH_3_	C-4′	H-3′, 5′	—

Comparison of the NMR spectra of **2** with that of crassicauline A (Chen et al., 2002), it was showed that compound **2** bore a methoxyl group at C-8 instead of an acetoxyl group, except for the absence of a methoxyl group at C-16 and the induction of a double bond at C-15 and C-16. Compound **2** exhibited nearly identical ^1^H and ^13^C NMR resonances to those of **1**, and the distinction between the two sets of spectra was demonstrated by the presence of an additional methoxyl group at C-8 in **2** instead of a hydroxyl group in **1**. All of the above arguments determined the structure of **2** as 16-demethoxy-∆^15(16)^-8-*O*-methylcrassicauline A, a new diterpenoid alkaloid (Supplementary Figures S14–S26).

16-demethoxy-∆^15(16)^-crassicauline A (**3**) was obtained as colorless needles, mp 155–157°C, ${\left[\alpha \right]}_{\text{D}}^{20}$ + 25.4°(*c* = 0.80, CH_3_OH). A protonated molecular ion at *m/z* 612.3163 (M + H)^+^ (cacld. 612.3121) in the HR-ESI-MS spectrum suggested a molecular formula of C_34_H_46_NO_9_. Compound **3** exhibited characteristic NMR features (Table 3) of an aconitine-type alkaloid (Pelletier et al., 1984), bearing an *N*-ethyl group (*δ*_H_ 1.05, brs, 3H; *δ*_H_ 2.50, 2.53, each 1H, m; *δ*_C_ 13.3 q, 49.3 t), three methoxyl groups (*δ*_H_ 3.15, 3.24, 3.27, each 3H, s; *δ*_C_ 57.4 q, 56.1 q, 59.2 q), an acetoxyl group (*δ*_H_ 1.39, 3H, s; *δ*_C_ 169.6 s, 21.8 q) (Joshi et al., 1987), as well as a *p*-methoxyl benzoyloxy group (*δ*_H_ 6.88, 7.94, each 2H, AA’BB’ system, *J* = 6.96 Hz; 3.85, 3H, s; *δ*_C_ 166.6 s, 122.4 s, 131.7 d (2C), 113.7 d (2C), 163.6 s, 55.4 q). The one-proton double signal (*J =* 4.8 Hz) at *δ*_H_ 4.92 was attributed to H-14β (Pelletier and Joshi, 1991; Gao et al., 2006), implying the appearance of *p*-methoxyl benzoyloxy group at C-14 position. The location of three methoxyl groups was based on the long-range correlations between 1-OCH_3_ (*δ*_H_ 3.24) and C-1, 6-OCH_3_ (*δ*_H_ 3.15) and C-6, 18-OCH_3_ (*δ*_H_ 3.27) and C-18 in HMBC spectrum (Figure 3). Furthermore, a hydroxyl group was assigned to C-13 based on the correlations between the C-13 and H-9, H-15 in the HMBC of **3**. The ^13^C-NMR signals at *δ* 125.4 and 137.3 showed that a disubstituted double bond was located at C-15 and C-16. The ^1^H-NMR signal at *δ* 6.53 (1H, d, *J* = 9.9 Hz, H-15) and *δ* 6.05 (1H, d, *J* = 9.9 Hz, H-16) also confirmed this deduction (Yue et al., 1994a; Yue et al., 1994b; Desai et al., 1998).

**TABLE 3:** ^1^H (600 MHz) and ^13^C (150 MHz) NMR data for compound **3** (CDCl_3_).

Position	*δ*_H_ (*J* in Hz)	*δ*_C_, type	HMBC	NOESY	^1^H-^1^H COSY
1	3.09 m	85.1, CH	1-OCH_3_	H-3β, H-10, 1-OCH_3_	H-2α, β
2α	2.27 m	26.2, CH_2_	—	—	H-1, H-2β, H-3α, β
2β	1.93 m		—	—	H-1, H-2α, H-3α, β
3α	1.70 m	35.1, CH_2_	—	H-18α	H-2α, β, H-3β
3β	1.65 m		—	H-1, H-5	H-2α, β, H-3α
4	—	39.3, C	—	—	—
5	2.09 d (6.6)	45.5, CH	—	H-3β, H-18α, β	H-6
6	4.10 d (6.6)	82.3, CH	C-4, C-8, C-17, 6-OCH_3_	H-9, 6-OCH_3_	H-5, H-7
7	3.00 brs	45.5, CH	C-9, C-11, C-15	H-15, 6-OCH_3_	H-6
8	—	83.7, C	—	—	—
9	2.92 m	44.4, CH	C-12, C-13, C-15	H-6	H-10, H-14
10	2.20 m	42.1, CH	—	H-1, H-14	H-9, H-12
11	—	49.9, C	—	—	—
12α	3.06 m	39.3, CH_2_	C-11	—	H-10, H-12β
12β	1.89 m		—	H-14	H-10, H-12α
13	—	76.2, C	—	—	—
14	4.92 d (4.8)	77.9, CH	C-8, C-16, ArC = O	H-10, H-12β	H-9
15	6.53 d (9.9)	125.4, CH	C-9, C-13	H-7, H-17	H-16
16	6.05 d (9.9)	137.3, CH	C-8	H-17	H-15
17	3.03 brs	62.8, CH	C-6, C-8, C-11	H-15, H-16, H-21, H-22	—
18α	3.26 d (8.82)	80.5, CH_2_	C-3, 18-OCH_3_	H-3α, H-5, H-19α, β, 18-OCH_3_	H-18β
18β	3.65 d (8.82)		C-3, C-19, 18-OCH_3_	H-5, H-19β, 18-OCH_3_	H-18α
19α	2.52 m	53.8, CH_2_	—	H-18α	H-19β
19β	2.56 m		—	H-18α, β	H-19α
21α	2.50 m	49.3, CH_2_	—	H-17	H-22
21β	2.53 m		—	H-17	H-22
22	1.05 brs	13.3, CH_3_	—	H-17	H-21α, β
1-OCH_3_	3.24 s	56.1, CH_3_	C-1	H-1	—
6-OCH_3_	3.15 s	57.4, CH_3_	C-6	H-6, H-7	—
18-OCH_3_	3.27 s	59.2, CH_3_	C-18	H-18α, β	—
8-C=O	—	169.6, C	—	—	—
CH_3_	1.39 s	21.8, CH_3_	8-C=O	H-2′, 6′	—
ArC = O	—	166.6, C	—	—	—
1′	—	122.4, C	—	—	—
2′, 6′	7.94 d (6.96)	131.7, CH	C-4′, ArC = O	8-CO-CH_3_	H-3′, 5′
3′, 5′	6.88 d (6.96)	113.7, CH	C-1′, C-4′	4′-OCH_3_	H-2′, 6′
4′	—	163.6, C	—	—	—
4′-OCH_3_	3.85 s	55.4, CH_3_	C-4′	H-3′, 5′	—

Comparison of the NMR data of **3** with crassicauline A (Chen et al., 2002) showed that except a disubstituted double bond, compound **3** lacked a methoxyl group at C-16. The NMR spectra of compound **3** were also similar to those of **1** and **2** except for the substituents at C-8. The C-8 substituents of **1** and **2** were hydroxyl group and methoxyl group, respectively, while **3** was acetoxyl group. The key NOE correlations (Figure 4) could be observed between 8-OAc and H-2′, 6′ in compound **3**. Therefore, the configuration of 8-OAc in **3** was unambiguously established to have a β-orientation. Thus, the structure of **3** was elucidated 16-demethoxy-∆^15(16)^-crassicauline A, a new diterpenoid alkaloid (Supplementary Figures S27–S39).

Pyrocrassicauline A (**4**) was obtained as colorless needles, mp 129–131°C, ${\left[\alpha \right]}_{\text{D}}^{20}$ + 182.4°(*c* = 0.81, CH_3_OH). Its molecular formula, determined to be C_33_H_45_NO_8_, was derived from HR-ESI-MS: *m/z* 584.3222 (M + H)^+^ (calcd. 584.3218). ^1^H and ^13^C NMR spectra of **4** (Supplementary Table S1) showed the distinct NMR features of an aconitine-type C_19_-diterpenoid alkaloid skeleton (Pelletier et al., 1984), bearing an *N*-ethyl group (*δ*_H_ 1.07, 3H, t, *J* = 7.2 Hz; *δ*_H_ 2.54, 2.66, each 1H, m; *δ*_C_ 13.6 q, 49.9 t), four methoxyl groups (*δ*_H_ 3.24, 3.28, 3.30, 3.38, each 3H, s; *δ*_C_ 56.4 q, 58.2 q, 59.3 q, 57.2 q), and a *p*-methoxyl benzoyloxy group (*δ*_H_ 6.88, 7.99, each 2H, AA’BB’ system, *J* = 8.4 Hz; 3.84, 3H, s; *δ*_C_ 167.9 s, 122.9 s, 132.0 d (2C), 113.5 d (2C), 163.3 s, 55.4 q). The one-proton double signal (*J* = 3.0 Hz) at *δ*_H_ 4.93 was attributed to H-14β (Pelletier and Joshi, 1991; Gao et al., 2006), implying the appearance of a *p*-methoxyl benzoyloxy group at C-14. Four methoxyl groups were assigned to C-1, C-6, C-16, C-18 due to the long-range correlations between 1-OCH_3_ (*δ*_H_ 3.24) and C-1, 6-OCH_3_ (*δ*_H_ 3.28) and C-6, 16-OCH_3_ (*δ*_H_ 3.38) and C-16, 18-OCH_3_ (*δ*_H_ 3.30) and C-18 in the HMBC spectrum of **4** (Figure 3). Along with the above-mentioned signals, its ^13^C NMR displayed eight oxygenated carbon signals, suggesting that compound **4** possessed an additional hydroxyl group, which could be located at C-13 due to the HMBC correlations between the C-13 and H-15 of **4**. Furthermore, the ^13^C NMR signals at *δ* 146.9 and 116.0 showed that a trisubstituted double bond was located at C-8 and C-15. The ^1^H NMR signal at *δ* 5.53 (1H, d, *J* = 6.0 Hz, H-15) also confirmed this deduction (Wang et al., 2009). The configuration of 16-OCH_3_ in **4** was also determined to have a β-orientation according to the key NOE correlations between 16-OCH_3_ and H-2′, 6′ (Figure 4). The ^13^C NMR spectra of **4** and crassicauline A were very similar except for the chemical shifts of C-8, C-15 signal appeared at low field, caused by the absence of an acetoxyl group and the presence of a double bond (Yue et al., 1994a; Yue et al., 1994b; Desai et al., 1998; Chen et al., 2002). All of the above arguments determined the structure of **4** as pyrocrassicauline A (Supplementary Figures S40–S53).

### Cardiotoxicity Assays

Intravenous injection of 0.10 mg/kg crassicauline A caused arrhythmias in normal rats, such as VPB, VT, and VF, accompanied by regular chest twitching and convulsions. The incubation period of crassicauline A induced arrhythmia was (182.8 ± 84.58) s. In contrast, the three converted products did not exhibit arrhythmias and behavioral manifestations under the same dose. Through this comparative experiment, it could be concluded that the cardiotoxicity was reduced after processing (Table 4; Supplementary Figure S54).

**TABLE 4:** Comparison of cardiotoxicity between crassicauline A and its converted products.

Compound	Dose (mg/kg)	VPB incubation period (s)	VT incidence (%)
Crassicauline A	0.10	182.8 ± 84.58	100
Compound **1**	0.10	—	0
Compound **2**	0.10	—	0
Compound **4**	0.10	—	0

“—”means that no arrhythmia occurred within 30 min after the administration of experimental compound. Data are expressed as mean ± S.D. (*n* = 10).

### Effects of Converted Products on VPB Incubation Period

VPB is the initial manifestation of aconitine-induced arrhythmia model rats, and the ECG of VPB is characterized by premature and bizarrely shaped QRS complexes that appear wide on the ECG, these complexes are not preceded by a P wave, and a T wave is usually oriented in a direction opposite the major deflection of the QRS (Casas et al., 2018; Bae and Kwon, 2019). VPB incubation period refers to the time after the administration of aconitine to the first occurrence of VPB (Qiu et al., 2016). The length of VPB incubation period can reflect the antiarrhythmic effect of the experimental compounds, the longer the incubation period is, the better the antiarrhythmic effect is.

In the control group, the typical characteristic ECGs of VPB and VT occurred successively following the injection of aconitine, even VF occurred in parts of the rats, and lasted for more than 30 min, suggesting that the arrhythmia model was successfully replicated. The VPB incubation period of the control group was (116.5 ± 36.4) s, lidocaine and propafenone groups were (280.3 ± 128.7) s and (193.3 ± 39.9) s, respectively. Two positive drugs had a significant difference in comparison with the control group (*p* < 0.05).

**FIGURE 5:**
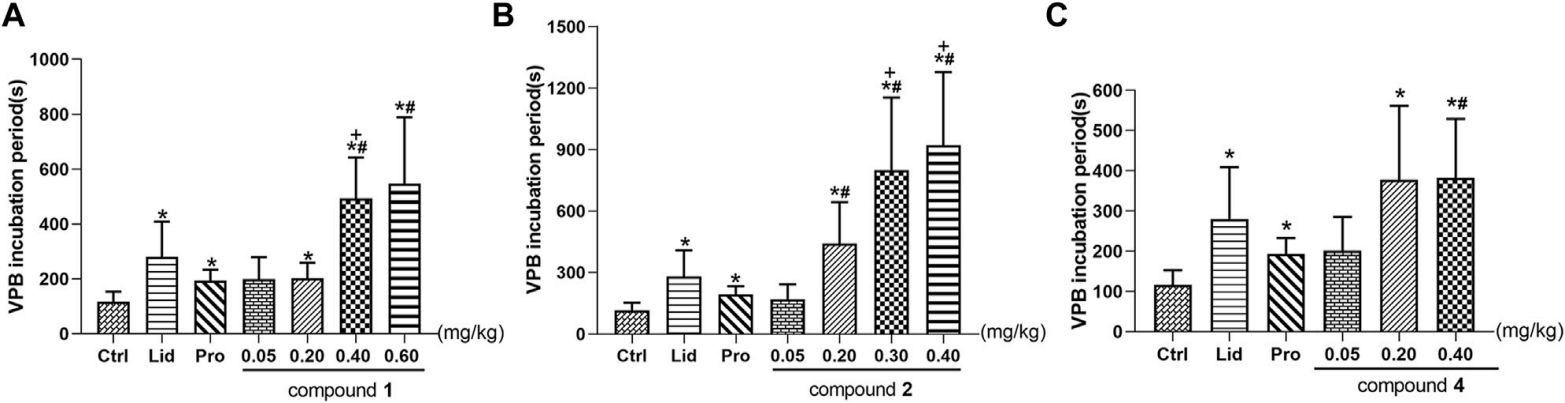
Effects of different experimental compounds on VPB incubation period **(A)**: compound **1**; **(B)**: compound **2**; **(C)**: compound **3**. Data are presented as mean ± SD. ^*^ *p* < 0.05 compared with control group, ^+^ *p* < 0.05 compared with the lidocaine group, ^#^ *p* < 0.05 compared with the propafenone group. Ctrl, the control group. Lid, lidocaine; Pro, propafenone.

The effects of compounds **1**, **2**, and **4** on VPB incubation period in rats are shown in Table 5; Figure 5. Compared with the control group, different dose groups of compound **1** (0.20, 0.40, and 0.60 mg/kg) significantly delayed the emerge of VPB (*p* < 0.05, Figure 5A). The VPB incubation periods in 0.40 and 0.60 mg/kg groups were (493.7 ± 148.7) s and (547.3 ± 241.8) s, respectively. They were significantly different from propafenone (*p* < 0.05). Similarly, there was a significant difference between 0.40 mg/kg group and lidocaine group (*p* < 0.05), indicating that a 0.40 mg/kg or more intravenous dose of compound **1** exhibited a marked activity relative to positive drugs.

**TABLE 5:** Effects of different compounds on VPB incubation period.

Group	Dose (mg/kg)	Number (with arrhythmia/total number)	VPB incubation period (s)
Control	—	10/10	116.5 ± 36.4
Lidocaine	5.0	12/21	280.3 ± 128.7*
Propafenone	3.2	10/10	193.3 ± 39.9*
Compound **1**	0.05	10/10	199.4 ± 79.7
	0.20	11/12	202 ± 56.4*
	0.40	10/13	493.7 ± 148.7*^+#^
	0.60	11/25	547.3 ± 241.8*^#^
Compound **2**	0.05	11/11	168.4 ± 74.0
	0.20	11/13	441.4 ± 202.3*^#^
	0.30	10/22	800.5 ± 353.2*^+#^
	0.40	10/28	920.5 ± 358.1*^+#^
Compound **4**	0.05	10/10	201.0 ± 84.9
	0.20	11/13	377.8 ± 184.0*
	0.40	19/25	382.9 ± 146.5*^#^

Data are presented as mean ± S.D. *p < 0.05 compared with control group, ^+^p < 0.05 compared with the lidocaine group, ^#^p < 0.05 compared with the propafenone group.

As mentioned above, the differentiation between compounds **1** and **2** is the substituents at C-8 position. To explore the effects of these structural distinctions, the antiarrhythmic effect of compound **2** was further evaluated. In comparison with the control group, different dose groups of compound **2** (0.20, 0.30, and 0.40 mg/kg) had a significant delay on the VPB incubation period (*p* < 0.05, Figure 5B). The VPB incubation periods in 0.20, 0.30, and 0.40 mg/kg groups were (441.4 ± 202.3) s, (800.5 ± 353.2) s, and (920.5 ± 358.1) s, respectively, which were significantly different from propafenone (*p* < 0.05). Compared with lidocaine, 0.30 and 0.40 mg/kg groups had significant difference (*p* < 0.05). It was demonstrated that a 0.30 mg/kg or more dose of compound **2** exhibited superior antiarrhythmic activity relative to compound **1** and the positive drugs.

On the contrary, compound **3**, bearing two ester bonds at C-8 and C-14, belonging to DDAs, may exert relatively strong toxicity (Gong, 2005). Consequently, its antiarrhythmic activity was not investigated. Different from compounds **1** and **2**, the double bond of compound **4** is located at C-8/C-15 rather than C-15/C-16. To exemplify different positions of double bonds on the strength of the antiarrhythmic efficacy, we further investigated the antiarrhythmic effect of compound **4**. Different dose groups of compound **4** (0.20 and 0.40 mg/kg) significantly delayed the onset time of VPB compared with the control group (*p* < 0.05, Figure 5C). The incubation period of VPB was (382.9 ± 146.5) s in the 0.40 mg/kg group, and it was significantly different from propafenone (*p* < 0.05), but showed an equivalent effect relative to lidocaine (*p* > 0.05).

In summary, within their own dose ranges, compounds **1**, **2**, and **4** could delay the onset time of VPB in a dose-dependent manner. Additionally, at the dose of 0.40 mg/kg, the VPB incubation periods were compound **2** >compound **1** >compound **4**, indicating that compound **2** had the best antiarrhythmic effect among these compounds.

### Effects of Converted Products on Incidence of VT

VT is the result of further progression of VPB, as well as an established risk factor for sudden cardiac death (Batiste et al., 2019). The ECGs of VT are characterized by three or more consecutive beats of apparent ventricular origin, the QRS complexes are wide and deformed, without constant P wave (Swerdlow et al., 1983; Baldzizhar et al., 2016; Brady et al., 2017). The incidence of VT can be used to evaluate whether the experimental compound can effectively prevent the progress of VPB, the lower the occurrence is, the better the efficacy is.

**FIGURE 6:**
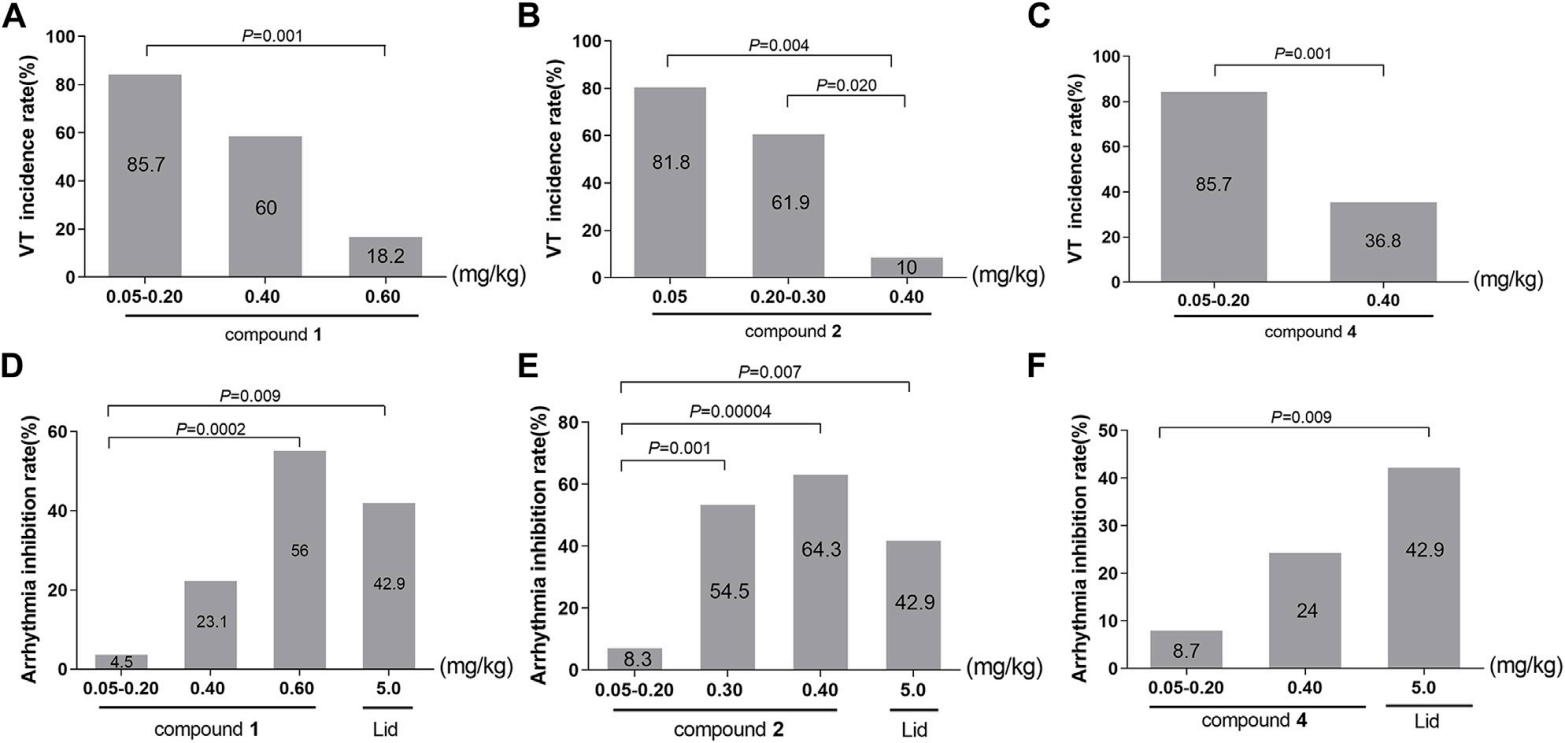
**(A–C)** Effects of converted products on the incidence of VT. **(A)**: compound **1**; **(B)**: compound **2**; **(C)**: compound **3**. **(D–F)** Effects of converted products on arrhythmia inhibition rate. **(D)**: compound **1**; **(E)**: compound **2**; **(F)**: compound **3**.

In accordance with the data requirements of chi-square test, the adjacent dose groups with similar VT incidence should be combined to carry out chi-square test. The subgroups of compound **1** were 0.05–0.20, 0.40, and 0.60 mg/kg. As vividly displayed in Table 6; Figure 6A, chi-square test showed a significant difference in the incidence of VT among the three groups (*χ* ^2^ = 14.704, *p* = 0.001). The pairwise comparison revealed a highly significant drop in the incidence of VT from 85.7% in the 0.05–0.20 mg/kg group to only 18.2% in the 0.60 mg/kg group (*χ* ^2^ = 11.313, *p* = 0.001).

**TABLE 6:** Chi-square analysis of the incidence of VT among different dose groups.

Compound	Dose (mg/kg)	VT (frequency)	Without VT (frequency)	*χ* ^2^	*p*
Compound **1**	0.05–0.20	18	3	14.704	0.001
	0.40	6	4		
	0.60	2	9		
Compound **2**	0.05	9	2	13.000	0.002
	0.20–0.30	13	8		
	0.40	1	9		
Compound **4**	0.05–0.20	18	3	10.165	0.001
	0.40	7	12		

Following the identical research approach as VPB incubation period, we further investigated the incidence of VT in compounds **2** and **4**. Compound **2** was divided into the 0.05, 0.20–0.30, and 0.40 mg/kg groups. As described in Table 6; Figure 6B, chi-square test showed that there was a marked difference in the incidence of VT among the three groups (*χ* ^2^ = 13.000, *p* = 0.002). In comparison with the 0.05 mg/kg (81.8%) and 0.20–0.30 mg/kg (61.9%) groups, the incidence of VT was significantly dropped to 10% in 0.40 mg/kg group (*χ* ^2^ = 8.144, *p* = 0.004; *χ* ^2^ = 5.422, *p* = 0.020).

The subgroups of compound **4** were 0.05–0.20 and 0.40 mg/kg groups. As presented in Table 6; Figure 6C, the chi-square test showed a highly difference in the incidence of VT between the two subgroups (*χ* ^2^ = 10.165, *p* = 0.001). Furthermore, there was a greatly significant reduction in the incidence of the VT from 85.7% in 0.05–0.20 mg/kg group to 36.8% in 0.40 mg/kg group (*χ* ^2^ = 10.165, *p* = 0.001).

On the basic of aforementioned results, it can be concluded that compounds **1**, **2**, and **4** could reduce the incidence of VT in a dose-dependent manner, suggesting that they could effectively prevent the further progression of VPB.

### Effects of Converted Products on Arrhythmia Inhibition Rate

The incidence of arrhythmia (Wang et al., 1997a; Wang et al., 1997b) is defined as the proportion of arrhythmia that occurs within 30 min after pre-intravenous injections of test drugs, followed by an arrhythmia model established with aconitine. The occurrence of any kinds of ECGs such as VPB, VT, or VF should be judged as arrhythmia. Arrhythmia inhibition rate (%) = 100%-arrhythmia incidence rate (%), that is, the proportion that no arrhythmia occurs within 30 min, which can be used to evaluate whether the experimental compound could completely inhibit the proarrhythmic effect of aconitine. Arrhythmia inhibition rate is the most intuitive index reflecting the strength of the drug efficacy, the higher it gets, the better the efficacy is.

Considering the number of rats without arrhythmia in dose groups of 0.05 and 0.20 mg/kg was small, the original grouping could not meet the requirements of the chi-square test. Therefore, adopting the similar data analysis method as VT incidence, chi-square analysis was carried out in this paper by combining 0.05 and 0.20 mg/kg groups into 0.05–0.20 mg/kg group in each experimental compound.

In this paper, the effects of different doses of compound **1** (0.05–0.20, 0.40, and 0.60 mg/kg) and lidocaine on arrhythmia inhibition rate in rats were compared. Chi-square test showed that the arrhythmia inhibition rates in the four groups were significantly different (*χ* ^2^ = 15.457, *p* = 0.001), as shown in Table 7. Compared with 0.05–0.20 mg/kg group, the arrhythmia inhibition rates in 0.60 mg/kg and lidocaine groups significantly increased from 4.5% to 56% and 42.9%, respectively (*χ* ^2^ = 14.258, *p* = 0.0002; *χ* ^2^ = 6.820, *p* = 0.009). Notably, the arrhythmia inhibition rate in 0.60 mg/kg group was higher than lidocaine, exhibiting a better effect, as shown in Figure 6D.

**TABLE 7:** Chi-square analysis of arrhythmia inhibition rate between different experimental compounds and lidocaine.

Compound	Dose (mg/kg)	Arrhythmia (frequency)	Without arrhythmia (frequency)	*χ* ^2^	*p*
Compound **1**	0.05–0.20	21	1	15.457	0.001
	0.40	10	3		
	0.60	11	14		
Lidocaine	5.0	12	9		
Compound **2**	0.05–0.20	22	2	18.123	0.0004
	0.30	10	12		
	0.40	10	18		
Lidocaine	5.0	12	9		
Compound **4**	0.05–0.20	21	2	6.908	0.032
	0.40	19	6		
Lidocaine	5.0	12	9		

To ascertain the influence of different substituents at C-8 and the location of double bond on drug efficacy, we further analysed the arrhythmia inhibition rate of compounds **2** and **4**, the result of chi-square test showed that the arrhythmia inhibition rates in compound **2** subgroups (0.05–0.20, 0.30, and 0.40 mg/kg) and lidocaine were statistically different (*χ* ^2^ = 18.123, *p* = 0.0004), as shown in Table 7. In comparison with 0.05–0.20 mg/kg group, the arrhythmia inhibition rate in 0.30 mg/kg, 0.40 mg/kg, and lidocaine groups significantly rose from 8.3% to 54.5%, 64.3%, and 42.9%, respectively (*χ* ^2^ = 11.578, *p* = 0.001; *χ* ^2^ = 17.093, *p* = 0.00004; *χ* ^2^ = 7.228, *p* = 0.007, respectively.), indicating that the antiarrhythmic effect of compound **2** was stronger with the increasing dose. Furthermore, a 0.30 mg/kg or more dosage of compound **2** exhibited a superior antiarrhythmic effect than lidocaine, as illustrated in Figure 6E.

Chi-square test showed that the arrhythmia inhibition rates in different dose groups (0.05–0.20 and 0.40 mg/kg) of compound **4** and lidocaine were significantly different (*χ* ^2^ = 6.908, *p* = 0.032), as shown in Table 7. The pairwise comparison result showed that the arrhythmia inhibition rate of lidocaine was significantly higher than 0.05–0.20 mg/kg group (*χ* ^2^ = 6.832, *p* = 0.009). However, a 0.40 mg/kg intravenously dose of compound **4** exhibited an equal antiarrhythmic activity relative to lidocaine (*p* > 0.05), as displayed in Figure 6F.

In summary, within their respective dose ranges, compounds **1**, **2**, and **4** could increase the arrhythmia inhibition rate in a dose-dependent manner. Among them, compound **1** in 0.60 mg/kg group (56%), compound **2** in 0.30 and 0.40 mg/kg groups (54.5%; 64.3%) had a higher arrhythmia inhibition rate than lidocaine (42.9%), manifesting that these two compounds possessed excellent antiarrhythmic effects.

## Discussion

In this paper, the HPLC method was used to investigate the structural transformation pathway of crassicauline A in sand frying process. Meanwhile, the cardiotoxicity between crassicauline A and its converted products was compared, the antiarrhythmic effect of the products was ultimately investigated. Considering crassicauline A is a monomer compound, readily adhering to the surface of the sand during processing, which is inconvenient to prepare and recover samples. In our previous study (Wang et al., 2020), it was confirmed that oil bath heating could simulate the process of sand frying, truly reflecting the dynamic variety of compounds during processing. In this paper, the processing parameters were determined according to the contents of crassicauline A and converted products in different processed samples. When processed at 160°C for 30 min, the converted products would get a relatively large quantity and higher content compared with other samples. As a result, the crassicauline A was processed, four converted products were further obtained by chromatographic techniques and elucidated by spectroscopic methods, which were identified as compounds **1**, **2**, **3**, and **4**, respectively.

**FIGURE 7:**
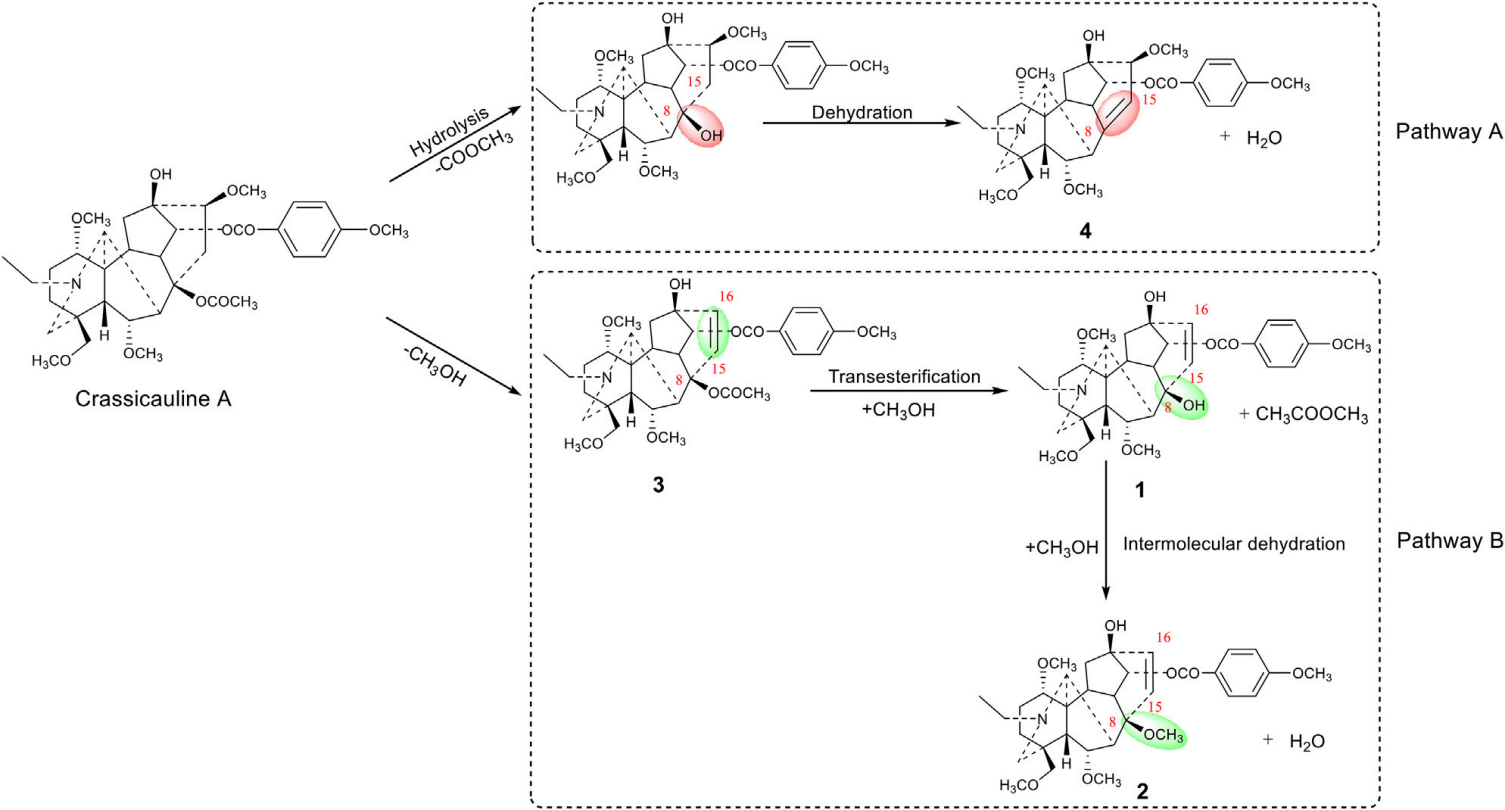
Transformation pathways of crassicauline A during sand frying process.

**FIGURE 8:**
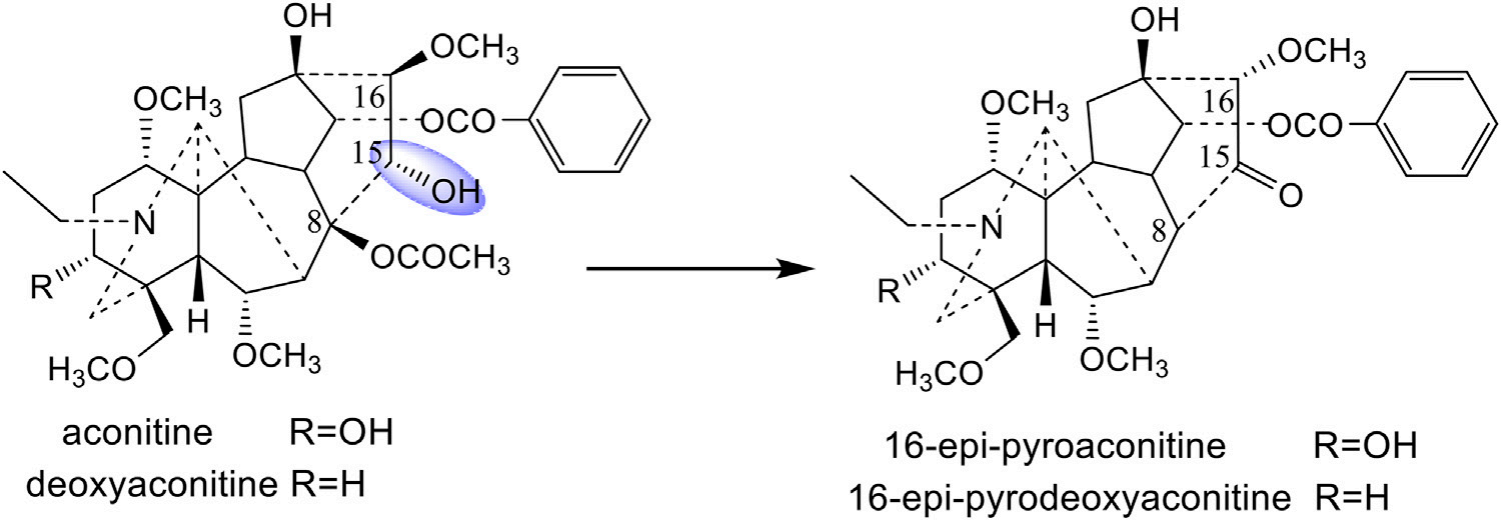
Prototype compounds and the corresponding sand-fried products (Wang et al., 2010b; Wang et al., 2011).

Crassicauline A is thermally unstable in structure and easily decomposed at high temperatures. Comparing the structures of the transformed products and the prototype compound, we found that the substituents at C-8, C-15, and C-16 of crassicauline A were converted after processing, and there might be at least two corresponding transformation pathways: (A) the acetoxyl group at C-8 of crassicauline A was firstly hydrolyzed to a hydroxyl group, a double bond was subsequently introduced at C-8/C-15 via further dehydration, which converted into compound **4**. (B) a double bond was firstly introduced at C-15/C-16 of crassicauline A by elimination of C-15 hydrogen atom and C-16 methoxyl group, to obtain compound **3**. The removed hydrogen atom and methoxyl group might generate methanol. The acetoxyl group at C-8 of compound **3** could undergo transesterification with methanol produced in the previous step, to generate compound **1** and methyl acetate. Finally, compound **2** was obtained by intermolecular dehydration of compound **1** with methanol (Figure 7). It was demonstrated that the structural transformation pathways of crassicauline A were different from those of aconitine and deoxyaconitine (Figures 7, 8, Wang et al., 2010b; Wang et al., 2011), which confirmed our speculation, the prototype alkaloids with different substituents at the C-15 position may have different structural transformation pathways.

Cardiotoxicity assays showed that intravenously administration of 0.10 mg/kg crassicauline A caused VPB, VT, and VF in normal rats, but no arrhythmias occurred when injecting the same dose of converted products. The cardiotoxicity of the transformed products was reduced relative to crassicauline A, indicating that the sand frying method could attenuate the cardiotoxicity of the parent compound.

Aconitine-induced arrhythmia model, a classical experimental modeling for arrhythmia, has long been used to test the efficacy of antiarrhythmic agents (Winslow, 1980; Xu et al., 2005). Aconitine mainly targets sodium channels and prolongs the open state, the cells are depolarized by a sustained Na^+^ influx, which accelerates the autonomy of the pacemaker and induces ectopic rhythm points, thus forming a multi-focal ectopic rhythm and shortening the myocardial refractory period, and finally leading to arrhythmia (Xu et al., 2005; Chan, 2009). The transformed products, as well as the parent compound, have structural similarities to aconitine. As mentioned above, some structurally similar diterpenoid alkaloids may exert opposite effects. We hypothesized that these converted alkaloids might have antiarrhythmic effects through an opposite mechanism to aconitine. Therefore, aconitine was chosen to establish the arrhythmia model in this paper for pharmacodynamic evaluation. Furthermore, concerning aconitine induces arrhythmia via continuously activating the sodium channel, two classical Class I sodium channel blockers, Class Ib lidocaine and Class Ic propafenone, were selected as positive drugs.

In this paper, three indicators, VPB incubation period, the incidence of VT, and the arrhythmia inhibition rate, were selected to comprehensively assess the antiarrhythmic activity of the three transformed products of crassicauline A. The experimental results manifested that, in their respective dose ranges, the transformed products could delay the VPB incubation period, decline the occurrence of VT and increase the arrhythmia inhibition rate. Within the dose ranges, if the optimal dose represented the strength of the antiarrhythmic activity of the tested compounds, the antiarrhythmic activities of the three transformed products would be ranked as follows. Compound **2** exhibited superior antiarrhythmic activity. At the dose of 0.40 mg/kg, 64.3% of the rats could not suffer from arrhythmia. And the onset time of VPB, among the rats undergoing arrhythmia, was found even at the point of approximately 15 min after aconitine administration, with the occurrence of VT being only 10%. Following compound **2**, compound **1** at a dose of 0.60 mg/kg exhibited a moderate antiarrhythmic activity, 56% of the rats were immune to arrhythmia. Within the rest rats, the arrhythmia was delayed for about 10 min. Among them, a 0.40 mg/kg intravenous dose of compound **4** produced the lowest antiarrhythmic effect. Only 24% of rats could be prevented from arrhythmia.

The antiarrhythmic structure-activity relationship acquired for the converted products exhibited promising antiarrhythmic effects relative to the parent compound crassicauline A. The activity can be manipulated by the positions of the double bond, as well as the substituents at the C-8 position. For example, compound **2** possessed a methoxyl group at C-8 instead of a hydroxyl group (compound **1**) exhibited superior antiarrhythmic activity. On the other hand, introduction of a double bond at C-8/C-15 (compound **4**) rather than at C-15/C-16 (compounds **1**, **2**) displayed a relatively weaker activity. These results indicated that a hydroxyl or a methoxyl group at C-8, elimination of C-16 methoxyl group, and a double bond at C-15/C-16 or C-8/C-15 might be important structural features to the antiarrhythmic activity of this kind of diterpenoid alkaloids. The structures of converted products could be beneficial in searching for the potential antiarrhythmic activity agents that are equal or more active, with lower toxicity, than antiarrhythmic drugs currently in clinical use.

## Conclusion

In summary, this paper used oil bath heating to simulate the process of sand frying, and screened out the temperature and time parameters for the structural transformation of crassicauline A, which provided a reference for the standardization and quantification of sand frying processing technology. In addition, it was found that the prototype alkaloids, which had different substituents at the C-15 position, might have different transformation pathways. Furthermore, it also demonstrated from the *in vivo* experiments that, with the structural transformation of crassicauline A, the converted products displayed lower cardiotoxicity and relatively strong antiarrhythmic effects. However, the converted products were not completely isolated, there might be other structural transformation pathways that remained investigate.

## Data Availability

The original contributions presented in the study are included in the article/Supplementary Material, further inquiries can be directed to the corresponding author.
