# Supplementary material for: Attenuation and Structural Transformation of Crassicauline A During Sand Frying Process and Antiarrhythmic Effects of its Transformed Products
Source: Front Pharmacol. 2021 Nov 2;12:734671. doi: 10.3389/fphar.2021.734671 (PMC8593248; doi:10.3389/fphar.2021.734671)
Supplement: Supplementary file 1 [file DataSheet1.PDF]

Supplementary Material

1 Supplementary Methods

Linearity, LOD and LOQ Crassicauline A standard solution was prepared by dissolving appropriate amounts of standard into 0.1% (v/v) HCl-methanol to achieve a concentration of 2.0 mg/mL. Accurately measuring 0.01, 0.1, 0.2, 0.8, and 1.0 mL into per 10 mL volumetric flask, 0.1% (v/v) HCl-methanol was added for dissolution to prepare solutions with concentrations of 2 μg/mL, 20 μg/mL, 40 μg/mL, 160 μg/mL, and 200 μg/mL. Calibration curve was plotted by using the peak areas (Y) versus the corresponding concentrations (X).

The limit of detection (LOD) and the limit of quantification (LOQ) were determined by injecting a series of standard solutions with appropriate concentrations until the signal-to-noise ratio (S/N) for the substance was 3 and 10 for LOD and LOQ, respectively.

Precision To evaluate precision, the HPLC analysis was performed on the standard solution consecutively six times in one day. The precision was evaluated by calculating the RSD value of the peak areas of crassicauline A.

Repeatability The repeatability was examined by injecting six different samples. Accurately adding 4 mL of reaction stock solution into 6 round-bottomed flasks, respectively. The samples were subsequently processed at 160 °C for 5 min, cooling to room temperature after reaction. The residue was diluted with 0.1% (v/v) HCl-methanol in a 10 mL volumetric flask, and subsequently filtered with 0.45 μm syringe filter before injection into the HPLC. The repeatability was evaluated by calculating the RSD value of the peak areas of crassicauline A.

Stability The standard solution was tested by HPLC analysis after storing them at 4 °C for 0, 2, 4, 6, 8, 12, and 24 h, respectively. The stability was determined by calculating the RSD value of the peak areas of crassicauline A.

2 Supplementary Figures and Tables

2.1 Supplementary Figures

2.1.1 Structure elucidation of 16-demethoxy-Δ$^{15(16)}$ -8-deacetylcrassicauline A (compound 1)

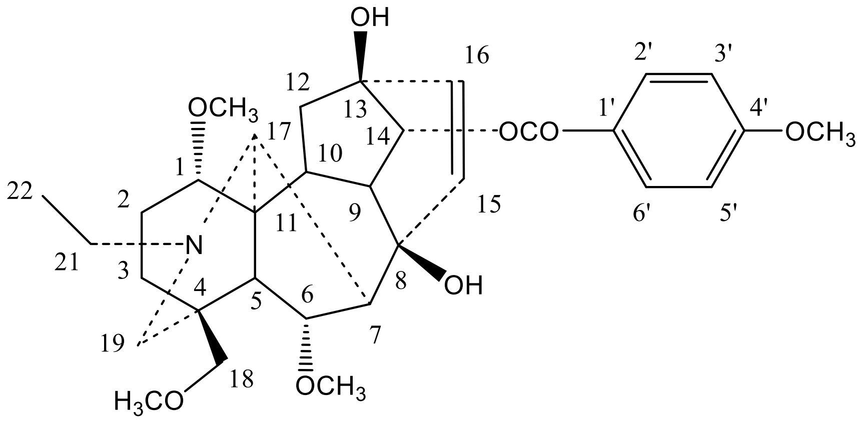

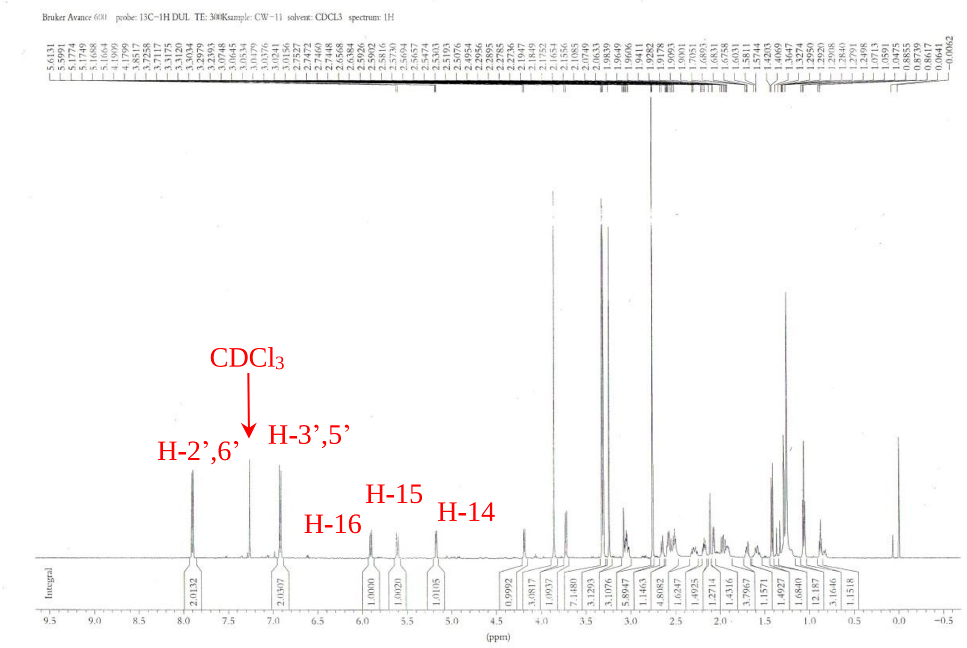

Supplementary Figure 1. ^{1}H NMR of 16-demethoxy-Δ$^{15(16)}$ -8-deacetylcrassicauline A.

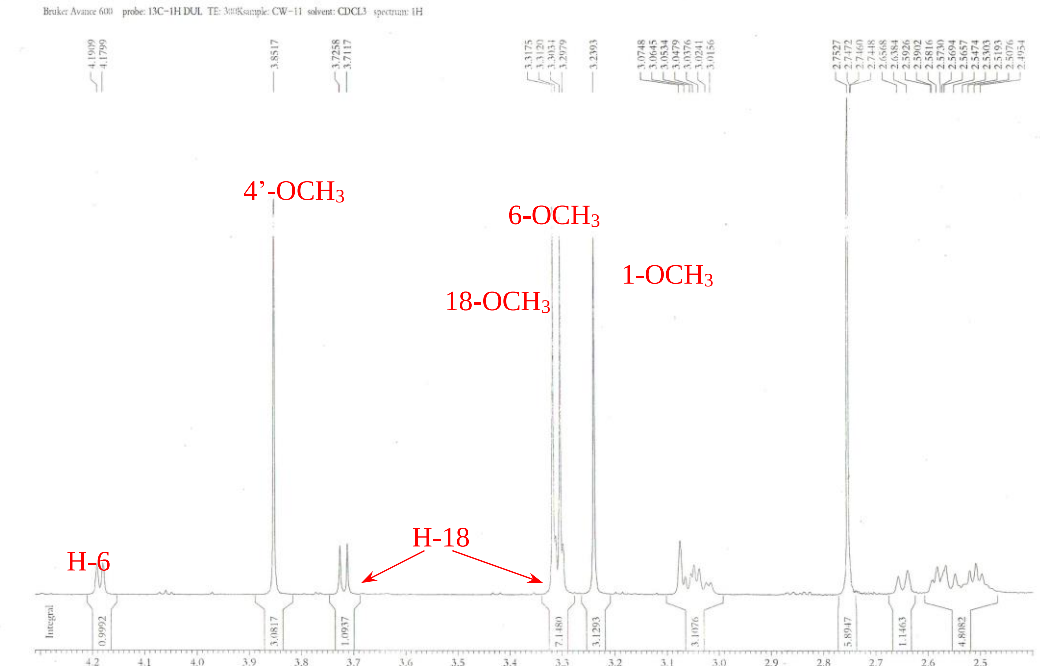

Supplementary Figure 2. ^{1}H NMR of 16-demethoxy-$\Delta^{15(16)}$ -8-deacetylcrassicauline A (Detail).

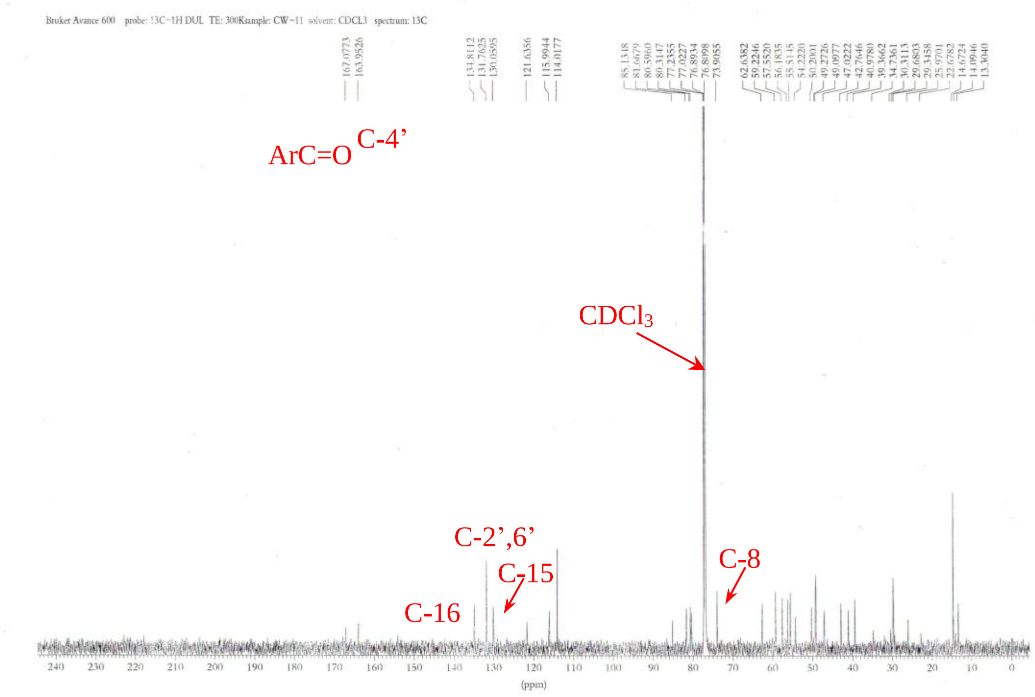

Supplementary Figure 3. ^{13}C NMR of 16-demethoxy-$\Delta^{15(16)}$ -8-deacetylcrassicauline A.

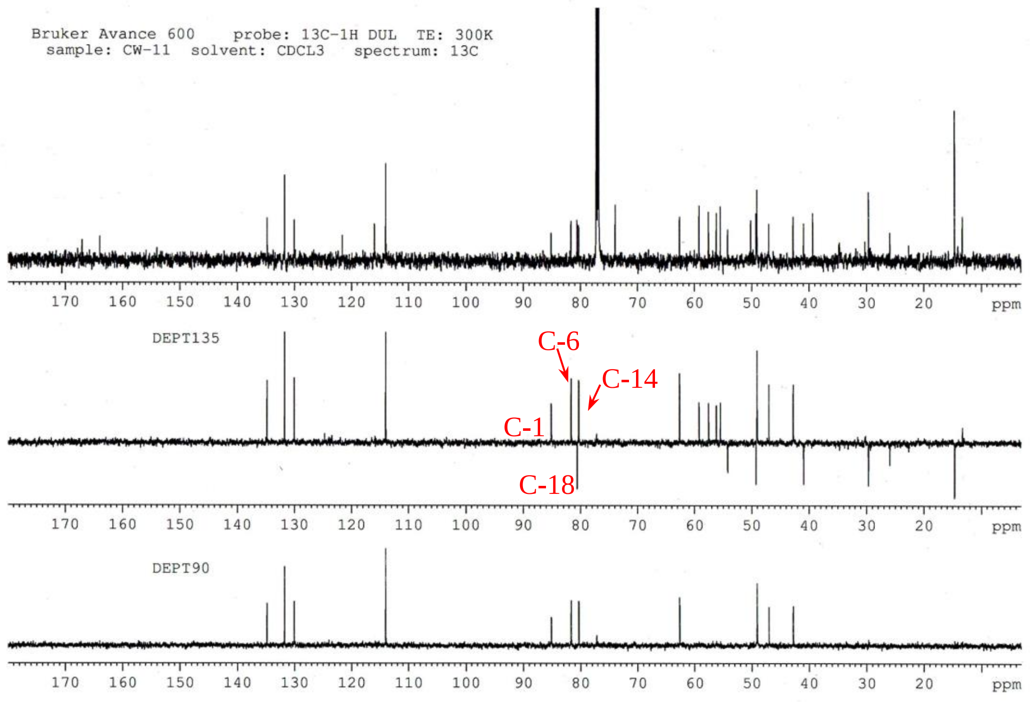

Supplementary Figure 4. DEPT of 16-demethoxy-$\Delta^{15(16)}$-8-deacetylcrassicauline A.

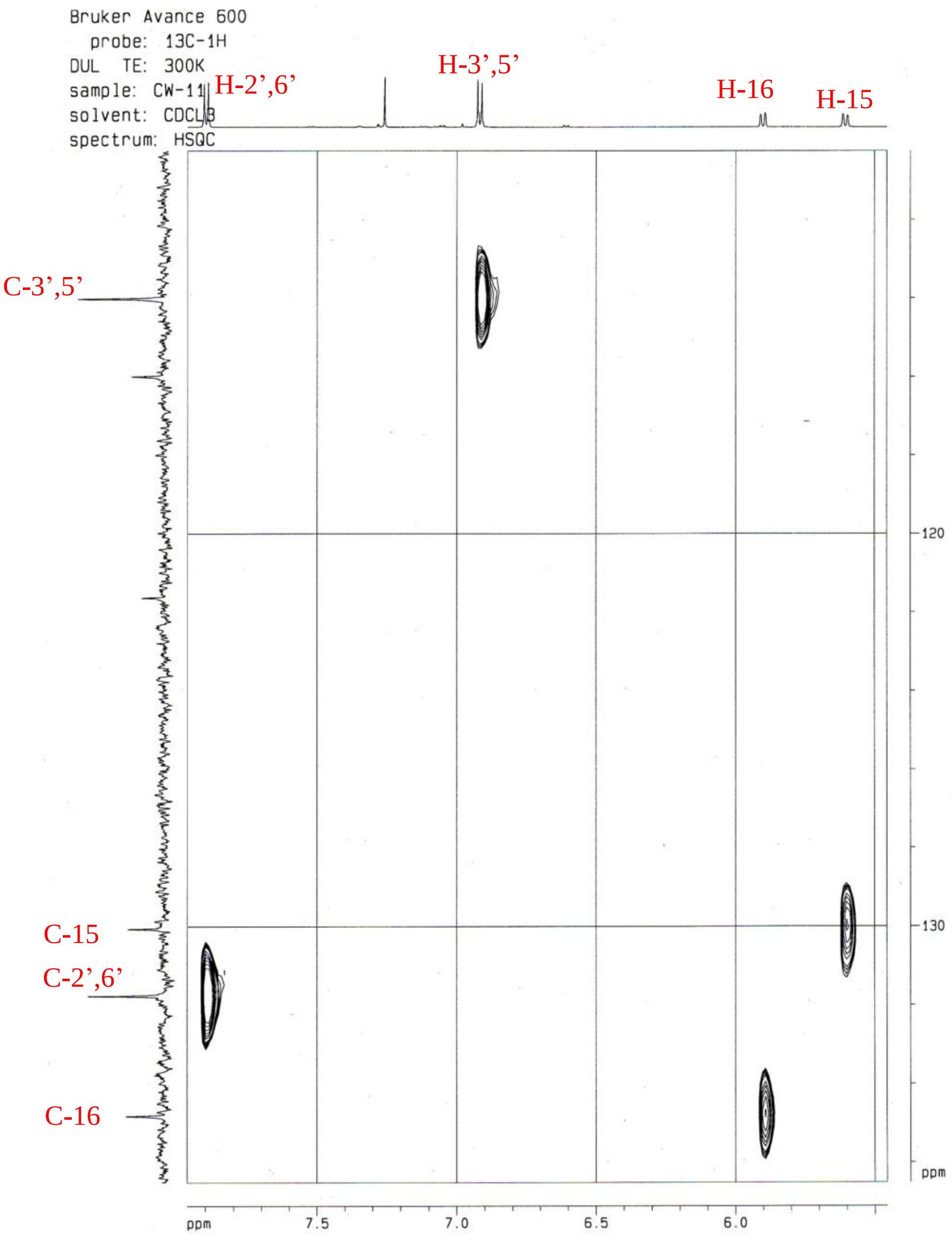

Supplementary Figure 5. Key HSQC correlations of 16-demethoxy-$\Delta^{15(16)}$-8-deacetylcrassicauline A (Detail 1).

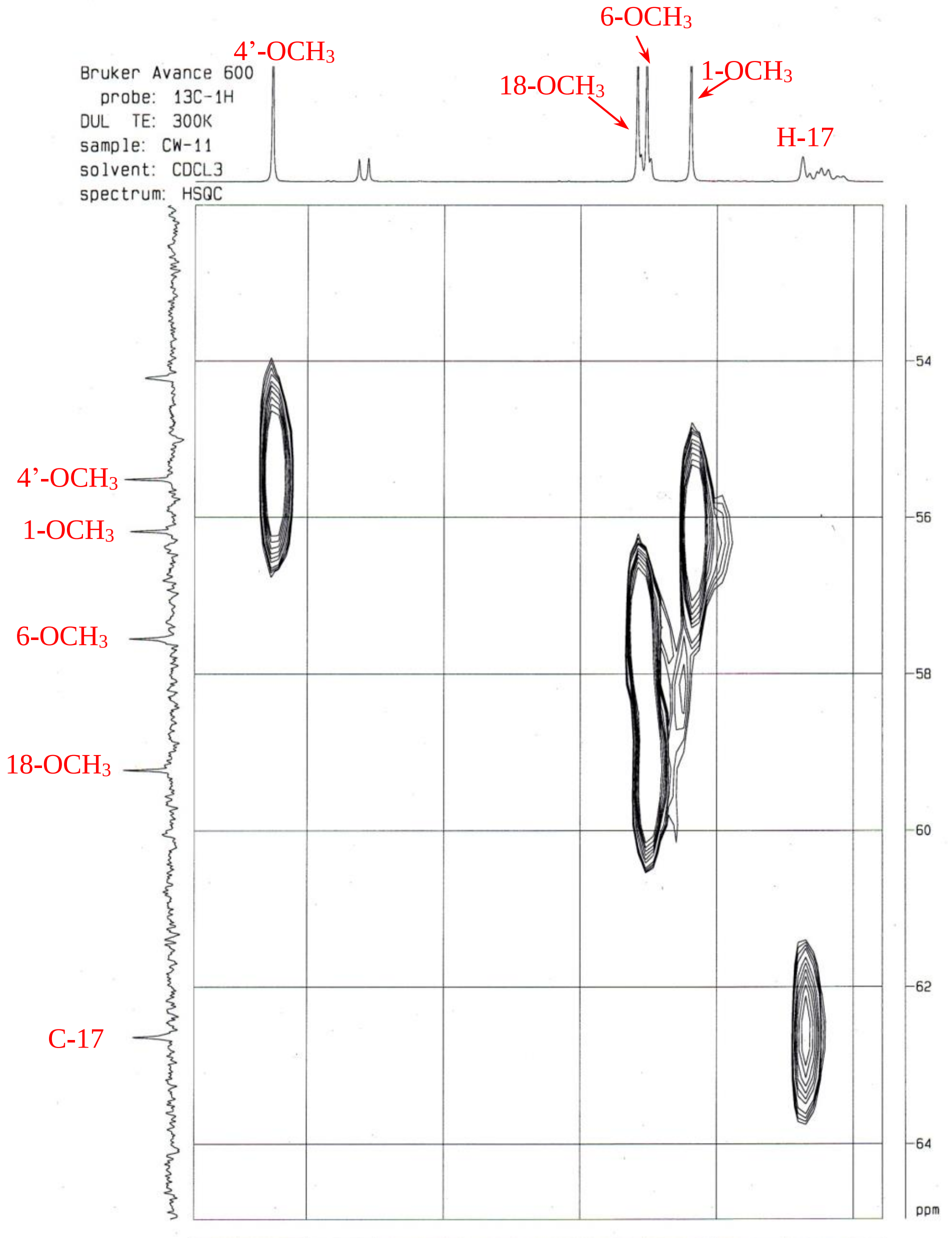

Supplementary Figure 6. Key HSQC correlations of 16-demethoxy-$\Delta^{15(16)}$-8-deacetylcrassicauline A (Detail 2).

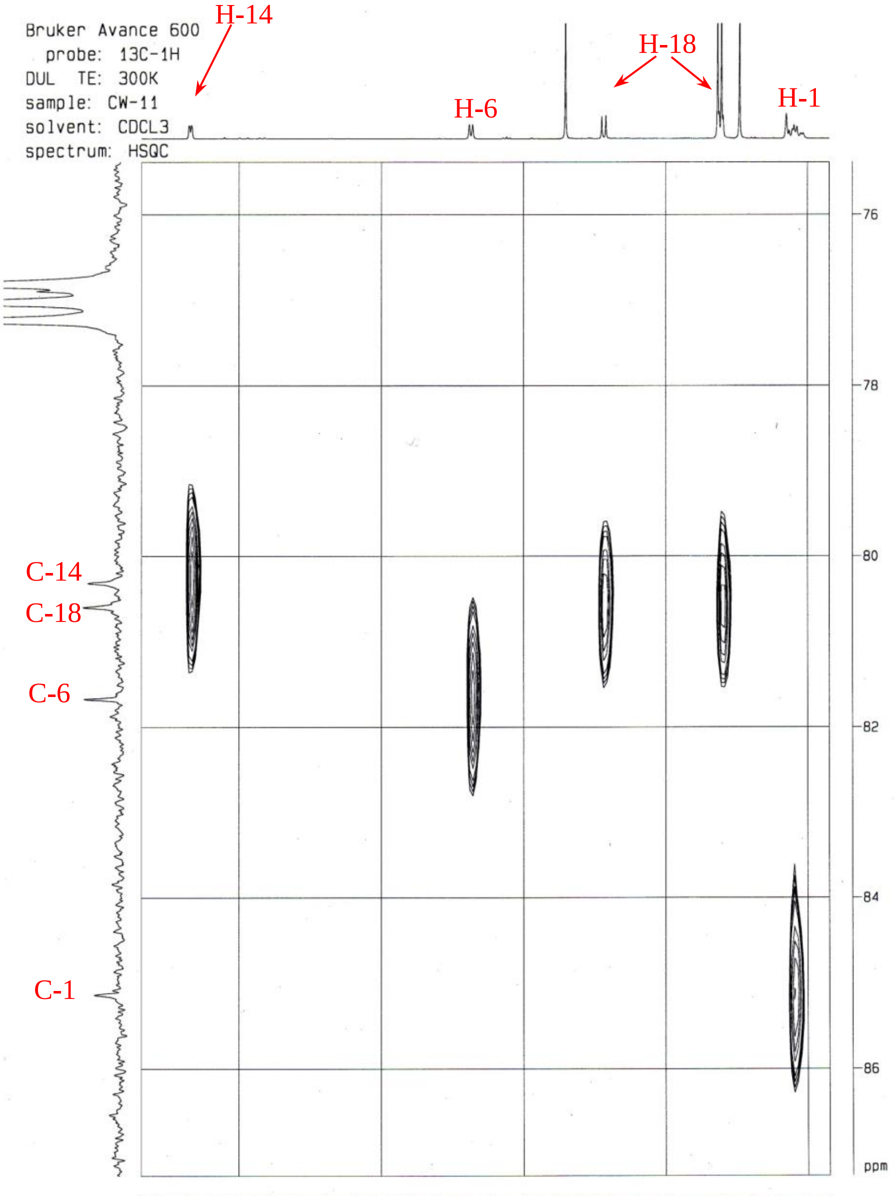

Supplementary Figure 7. Key HSQC correlations of 16-demethoxy-$\Delta^{15(16)}$-8-deacetylcrassicauline A (Detail 3).

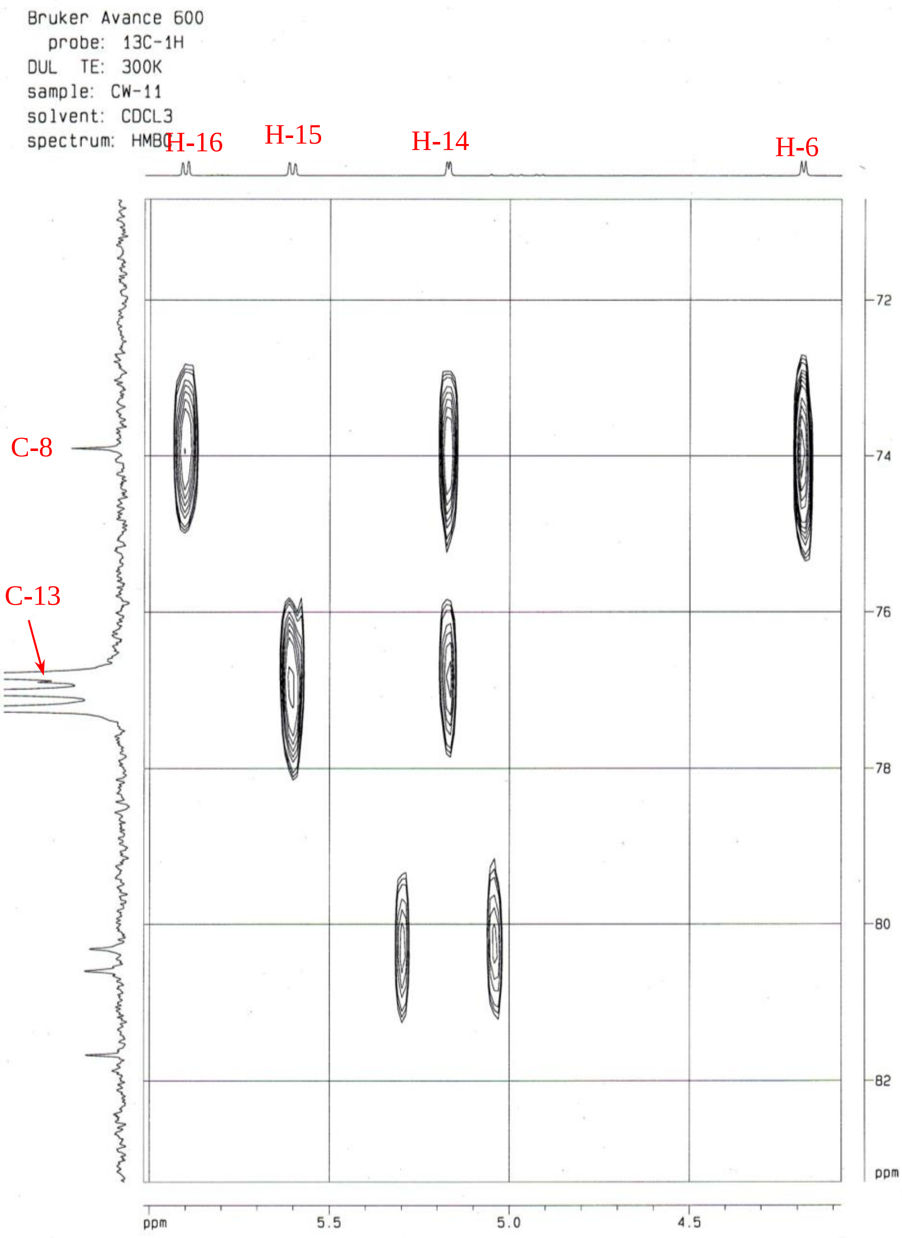

Supplementary Figure 8. Key HMBC correlations of 16-demethoxy-Δ$^{15(16)}$ -8-deacetylcrassicauline A (Detail 1).

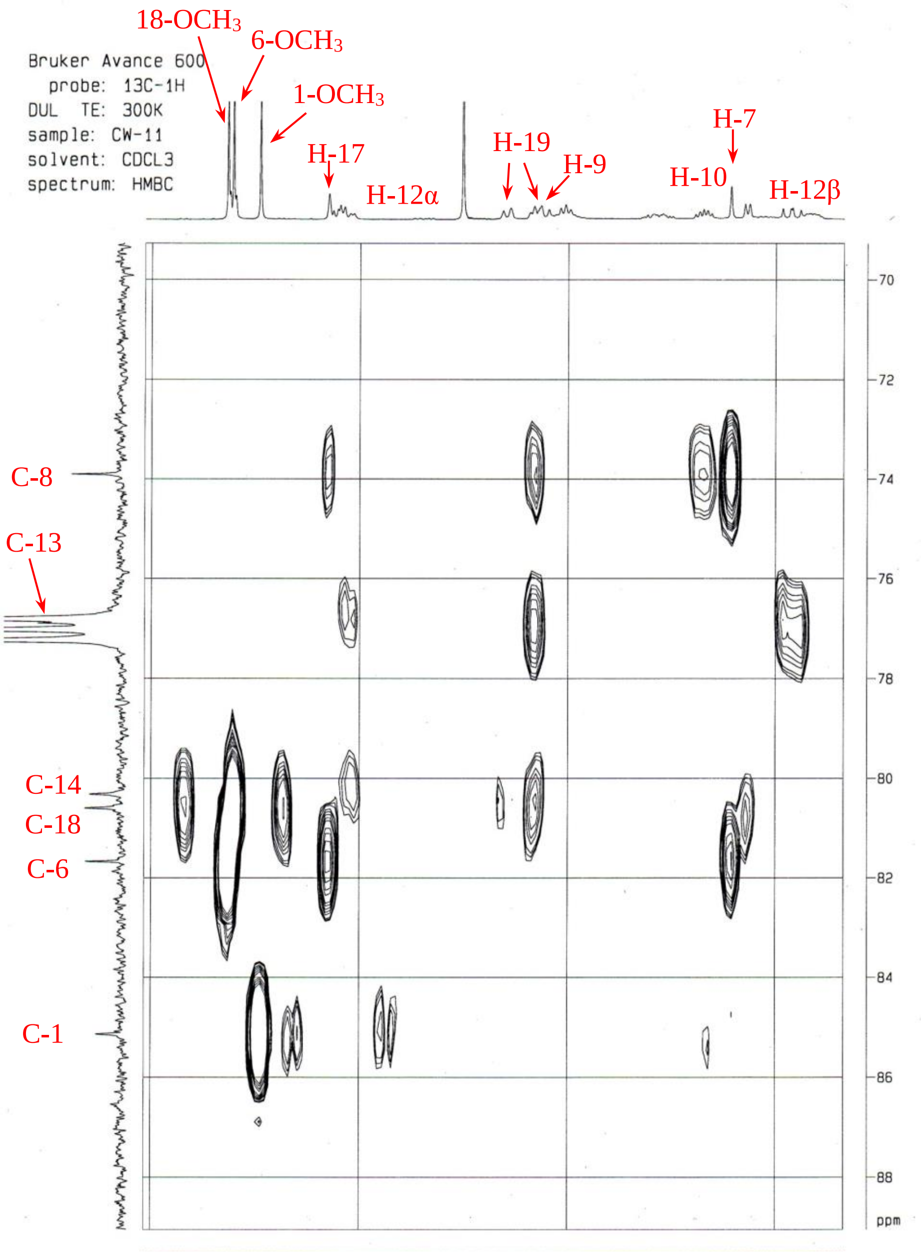

Supplementary Figure 9. Key HMBC correlations of 16-demethoxy-$\Delta^{15(16)}$ -8-deacetylcrassicauline A (Detail 2).

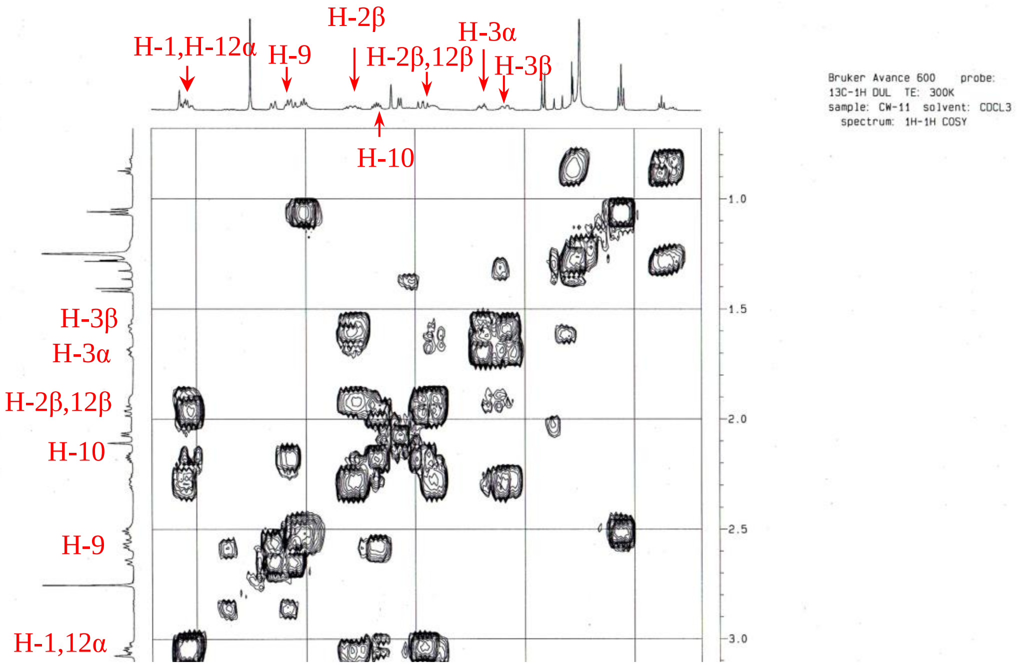

Supplementary Figure 10. Key ^{1}H-^{1}H COSY correlations of 16-demethoxy-$\Delta^{15(16)}$ -8-deacetylcrassicauline A.

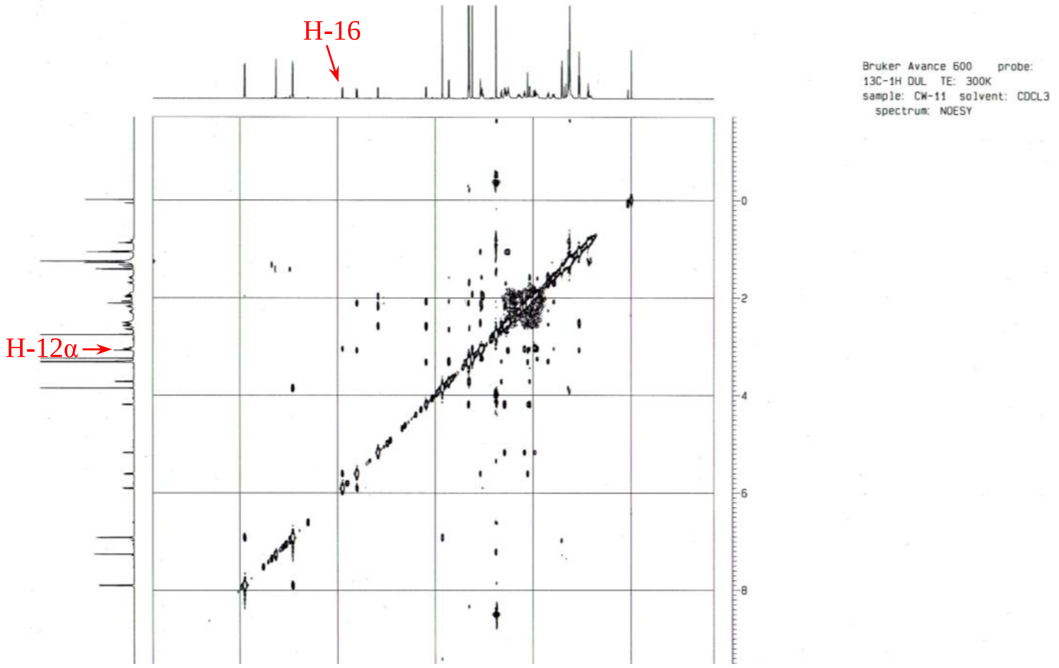

Supplementary Figure 11. Key NOESY correlations of 16-demethoxy-$\Delta^{15(16)}$-8-deacetylcrassicauline A.

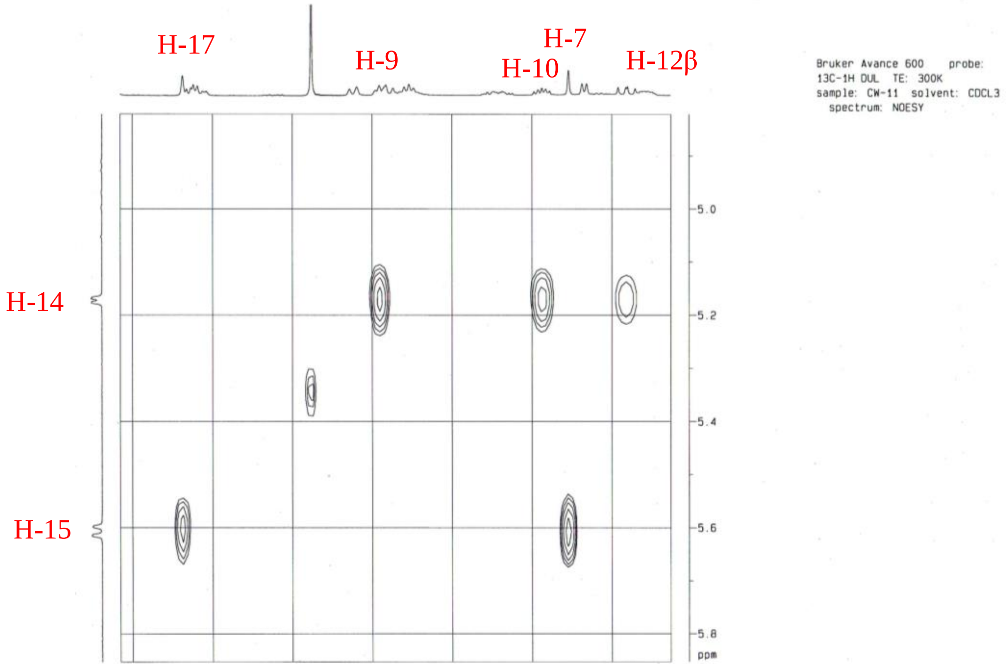

Supplementary Figure 12. Key NOESY correlations of 16-demethoxy-$\Delta^{15(16)}$-8-deacetylcrassicauline A (Detail).

Mass Spectrum SmartFormula Report

Analysis Info

Analysis Name	D:\Data\USER-2020\CW-11.d	Acquisition Date	7/30/2020 2:52:57 PM
Method	tune_low_NEW.m	Operator	Ma
Sample Name	CW-11	Instrument / Ser#	micrOTOF-Q II 10203
Comment			

Acquisition Parameter

Source Type	ESI	Ion Polarity	Positive	Set Nebulizer	0.8 Bar
Focus	Not active	Set Capillary	4500 V	Set Dry Heater	180 °C
Scan Begin	50 m/z	Set End Plate Offset	-500 V	Set Dry Gas	6.0 l/min
Scan End	1500 m/z	Set Collision Cell RF	150.0 Vpp	Set Divert Valve	Source

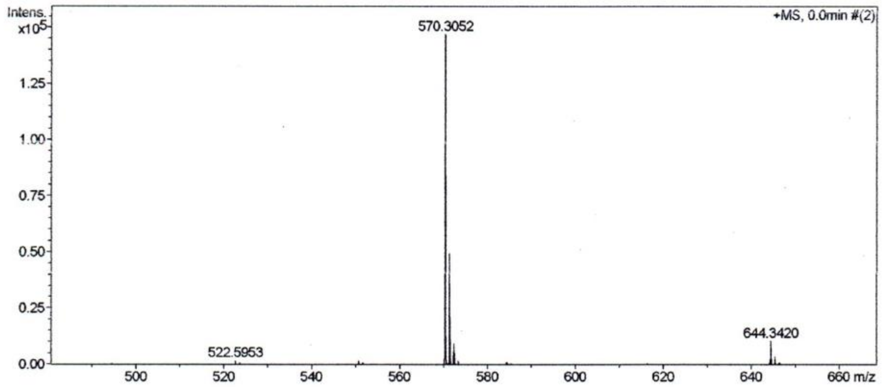

Meas. m/z	Formula	m/z	err [ppm]	mSigma	N-Rule	e⁻ Conf
570.3052						
	C 32 H 44 N O 8	570.3061	1.7	11.14	ok	even

Bruker Compass DataAnalysis 4.0 Page 1 of 1

Supplementary Figure 13. High-resolution ESI-MS of 16-demethoxy-$\Delta^{15(16)}$ -8-deacetylcrassicauline A.

2.1.2 Structure elucidation of 16-demethoxy-Δ$^{15(16)}$-8-*O*-methylcrassicauline A (compound 2)

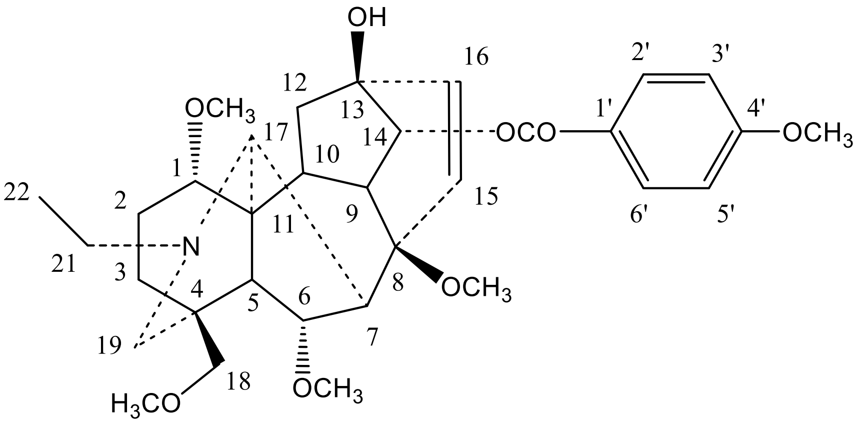

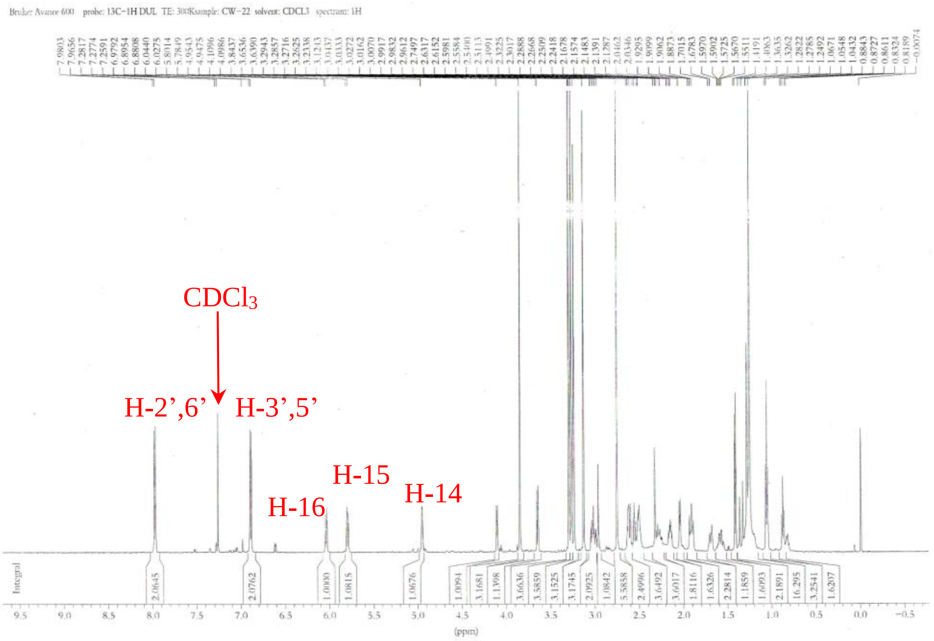

Supplementary Figure 14. ^{1}H NMR of 16-demethoxy-Δ$^{15(16)}$-8-*O*-methylcrassicauline A.

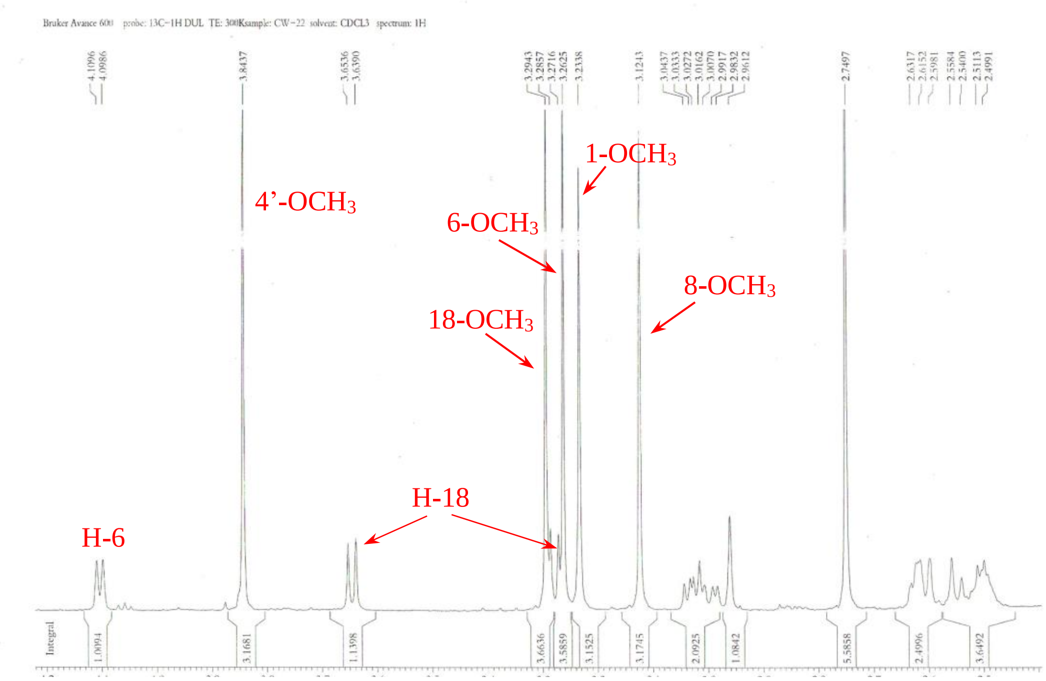

Supplementary Figure 15. ^{1}H NMR of 16-demethoxy-$\Delta^{15(16)}$-8-*O*-methylcrassicauline A (Detail).

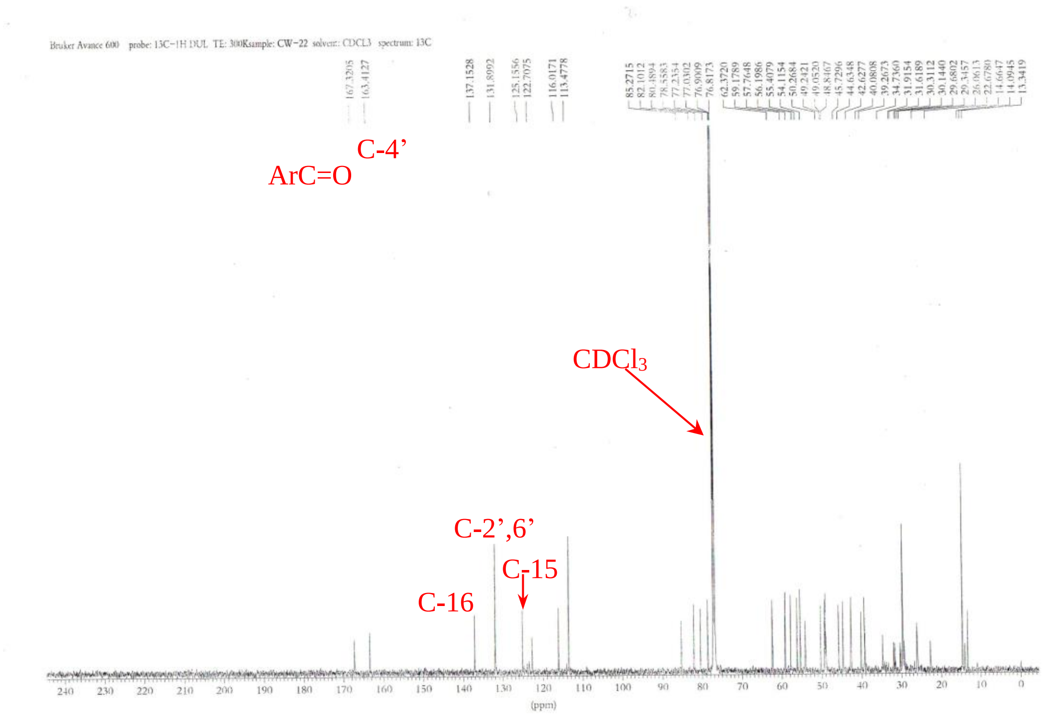

Supplementary Figure 16. ^{13}C NMR of 16-demethoxy-$\Delta^{15(16)}$-8-*O*-methylcrassicauline A.

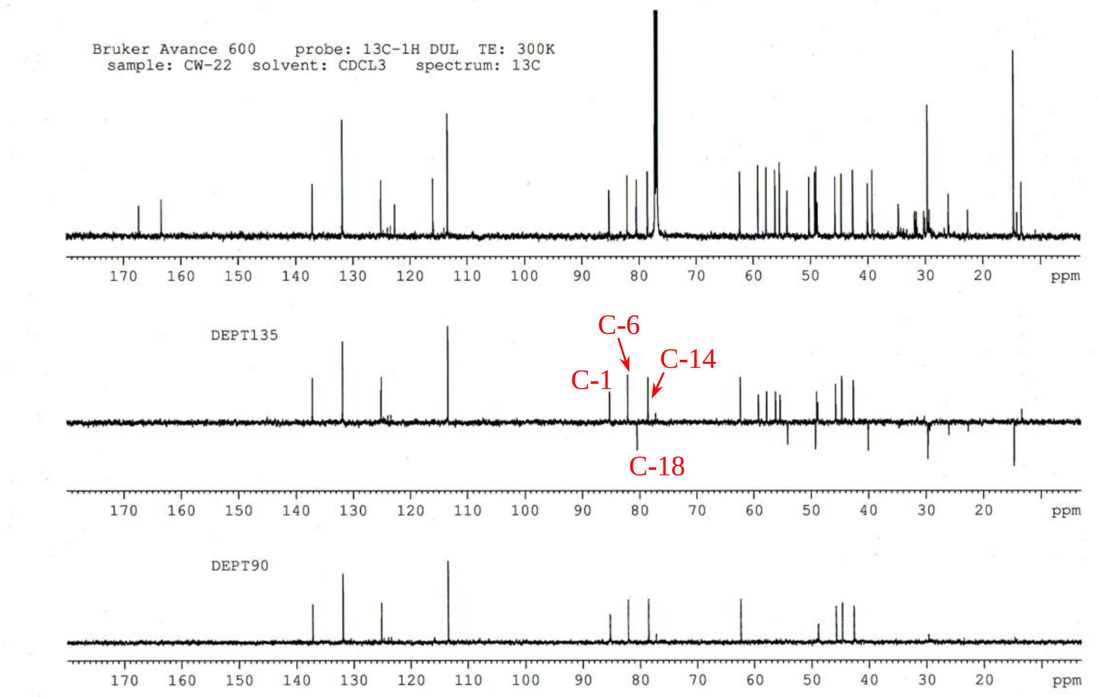

Supplementary Figure 17. DEPT of 16-demethoxy-$\Delta^{15(16)}$-8-*O*-methylcrassicauline A.

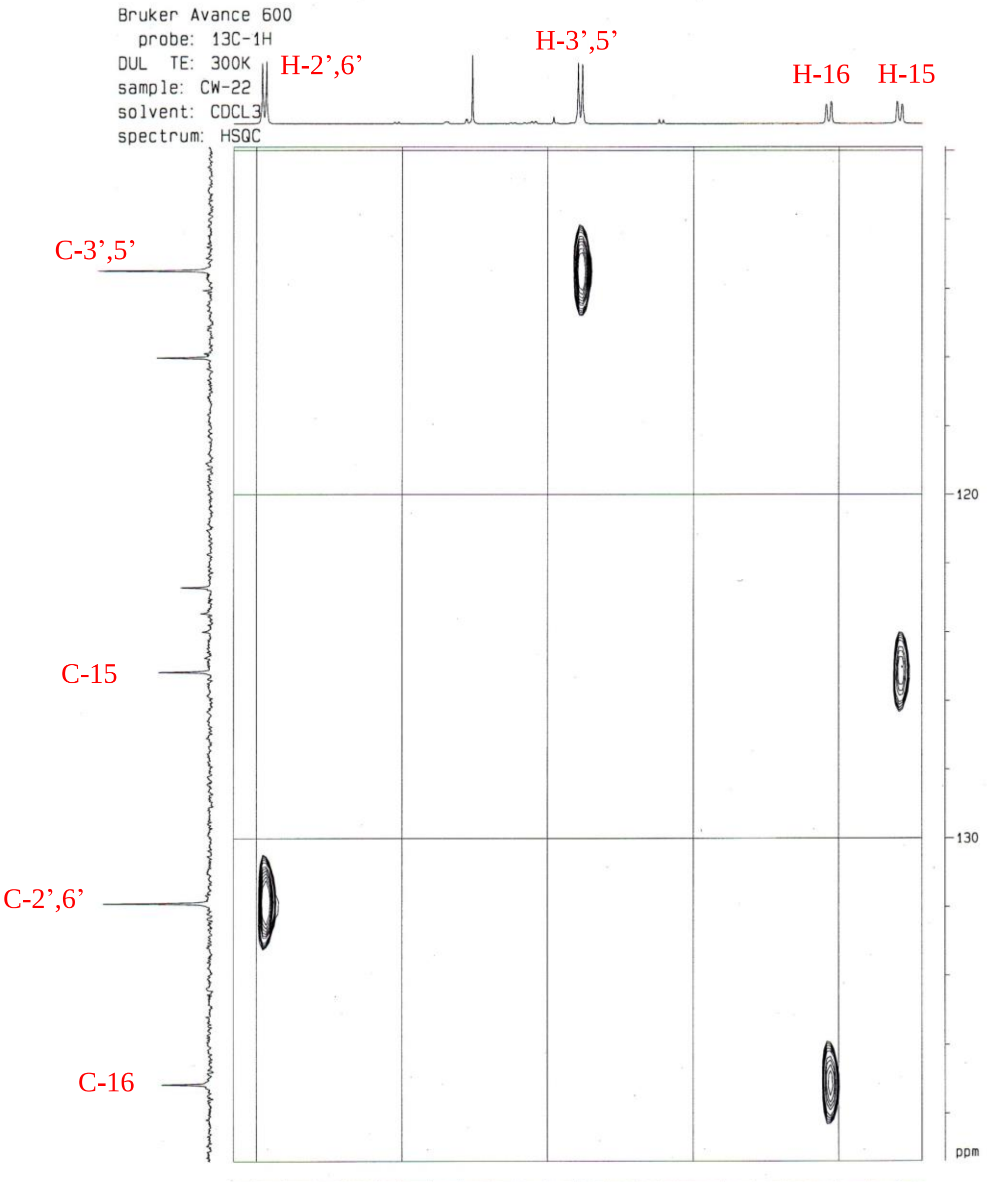

Supplementary Figure 18. Key HSQC correlations of 16-demethoxy-$\Delta^{15(16)}$-8-*O*-methylcrassicauline A (Detail 1).

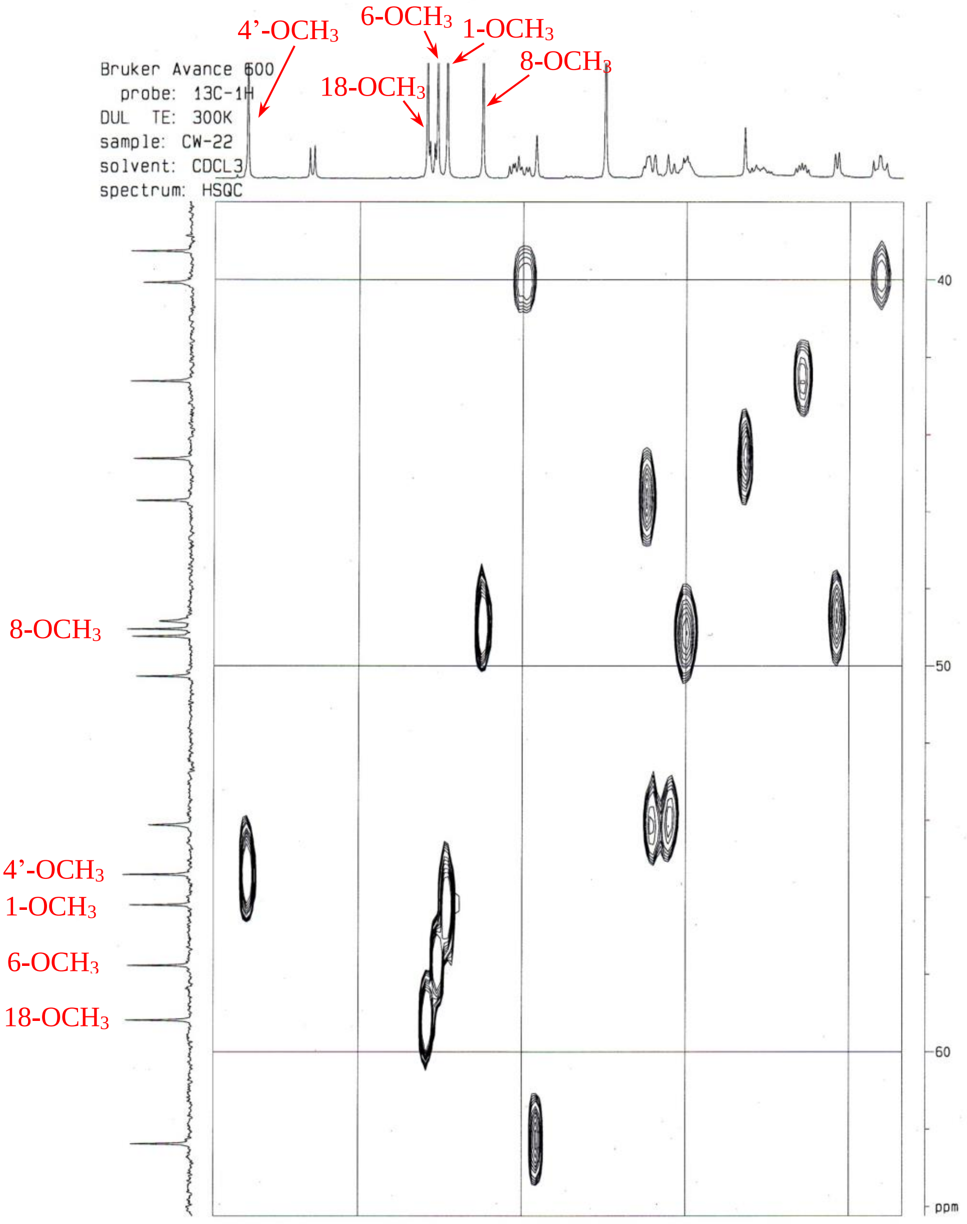

Supplementary Figure 19. Key HSQC correlations of 16-demethoxy-$\Delta^{15(16)}$-8-*O*-methylcrassicauline A (Detail 2).

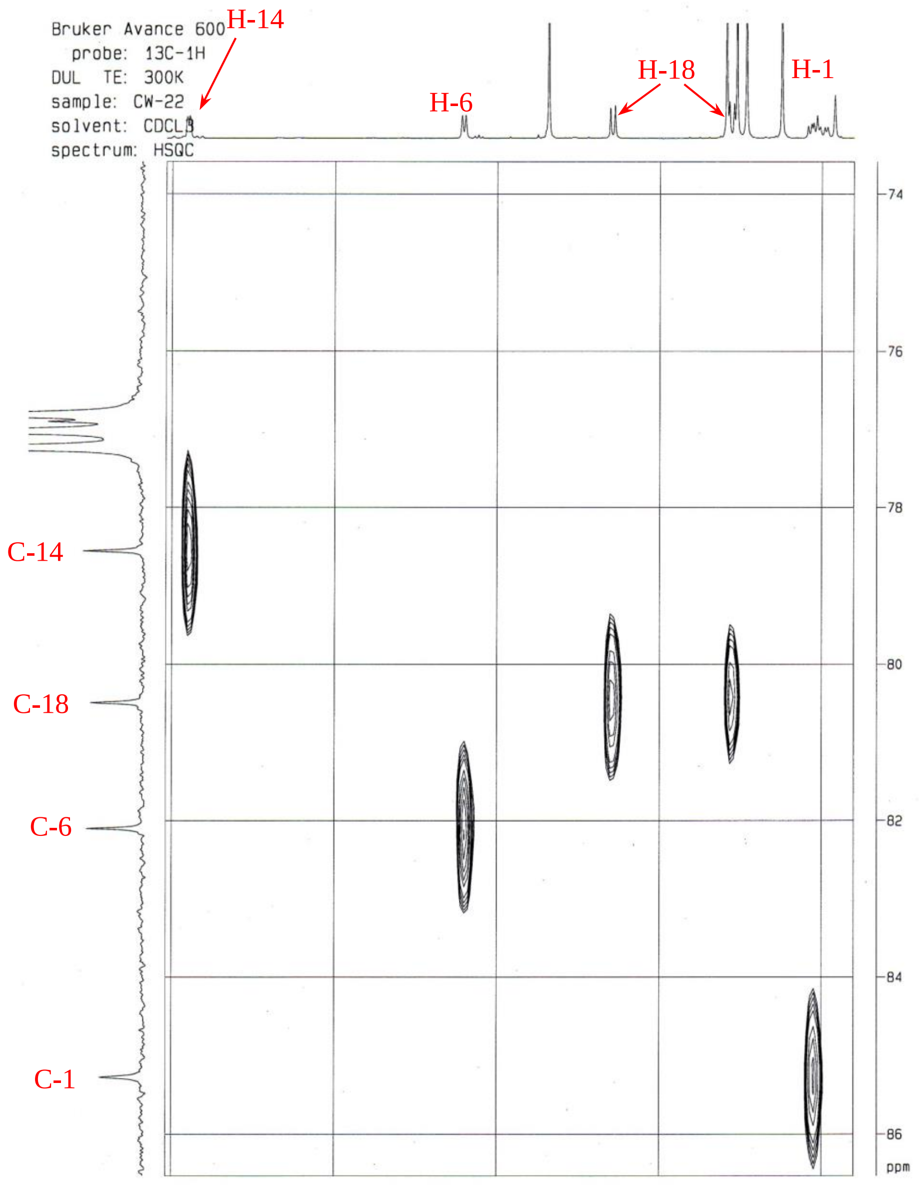

Supplementary Figure 20. Key HSQC correlations of 16-demethoxy-$\Delta^{15(16)}$-8-*O*-methylcrassicauline A (Detail 3).

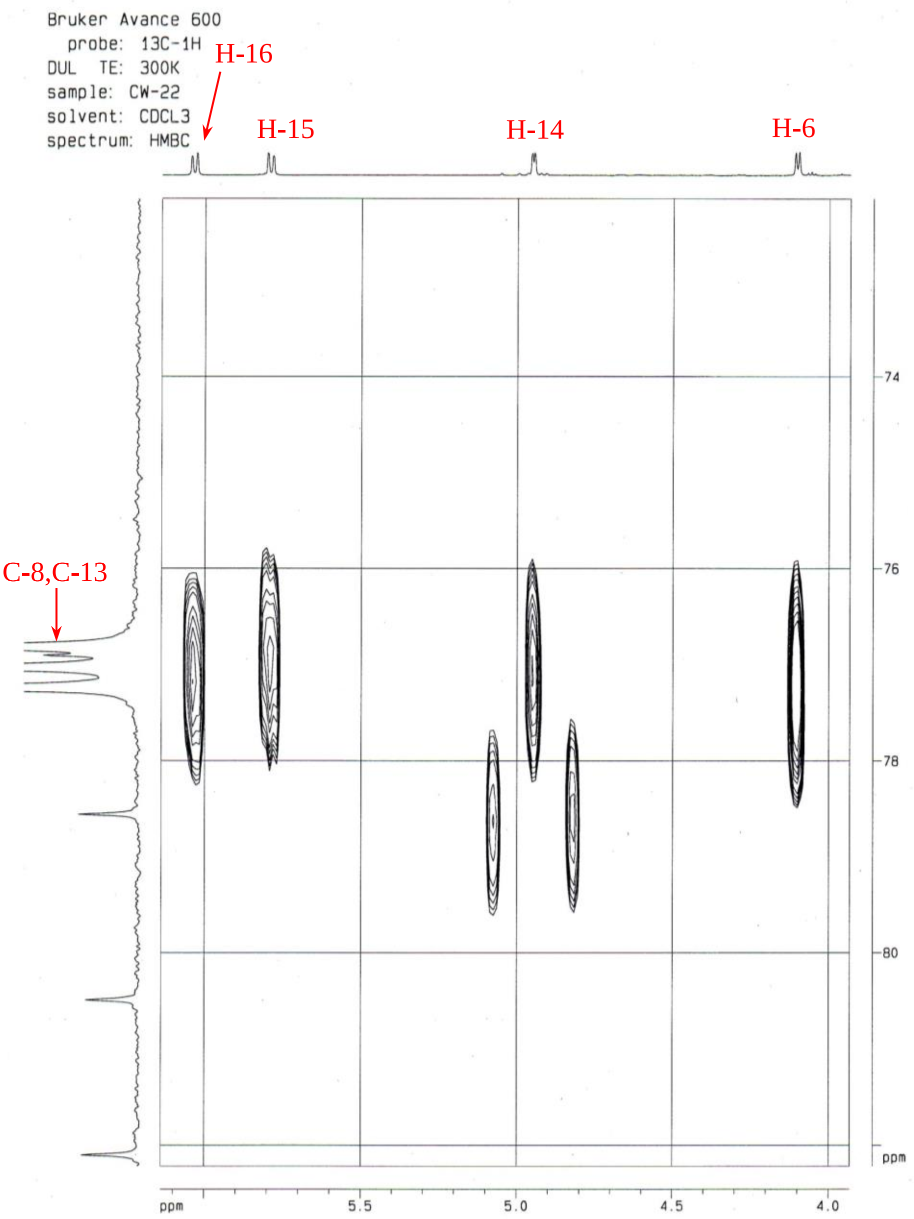

Supplementary Figure 21. Key HMBC correlations of 16-demethoxy-$\Delta^{15(16)}$-8-*O*-methylcrassicauline A (Detail 1).

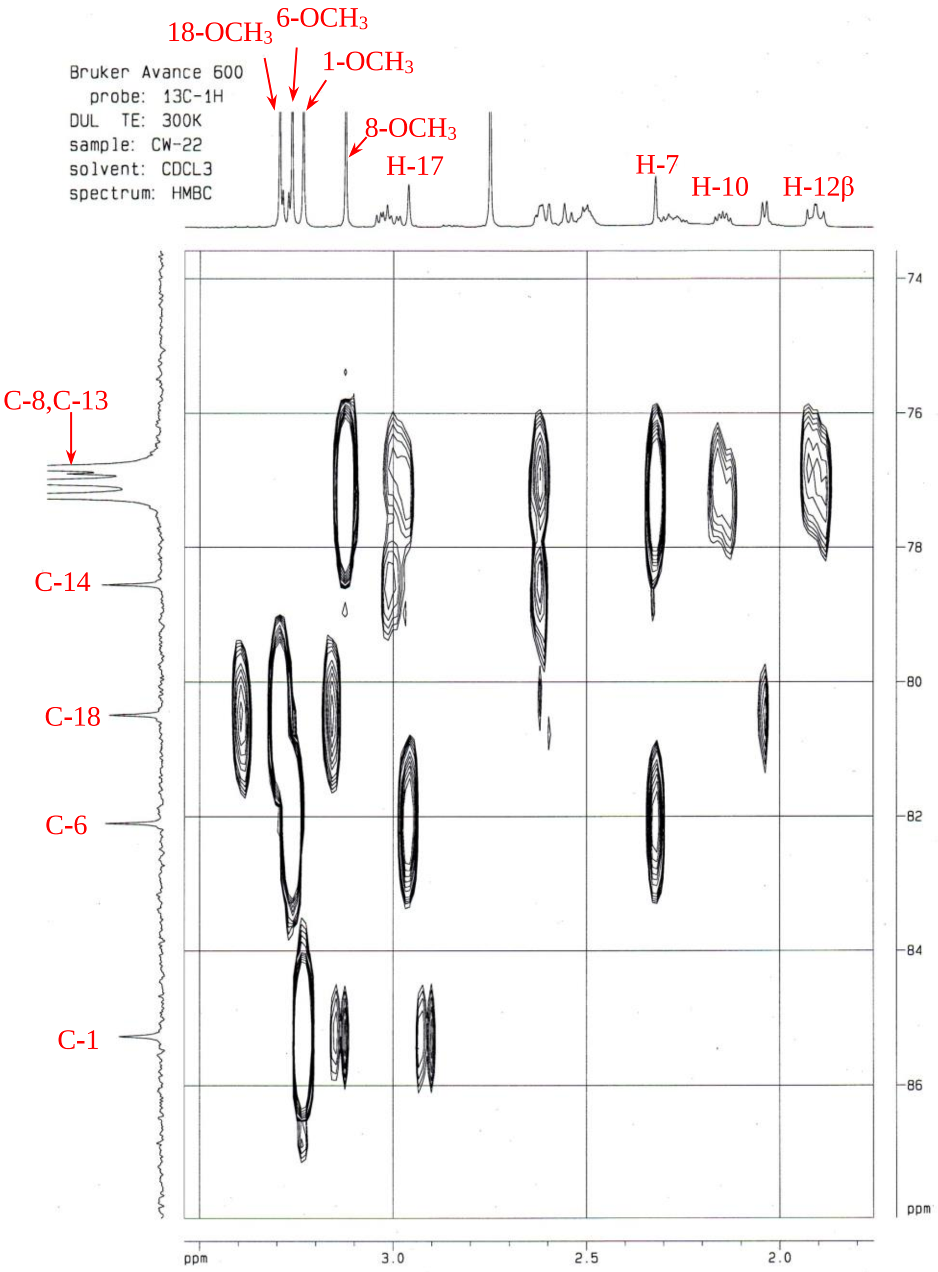

Supplementary Figure 22. Key HMBC correlations of 16-demethoxy-$\Delta^{15(16)}$-8-*O*-methylcrassicauline A (Detail 2).

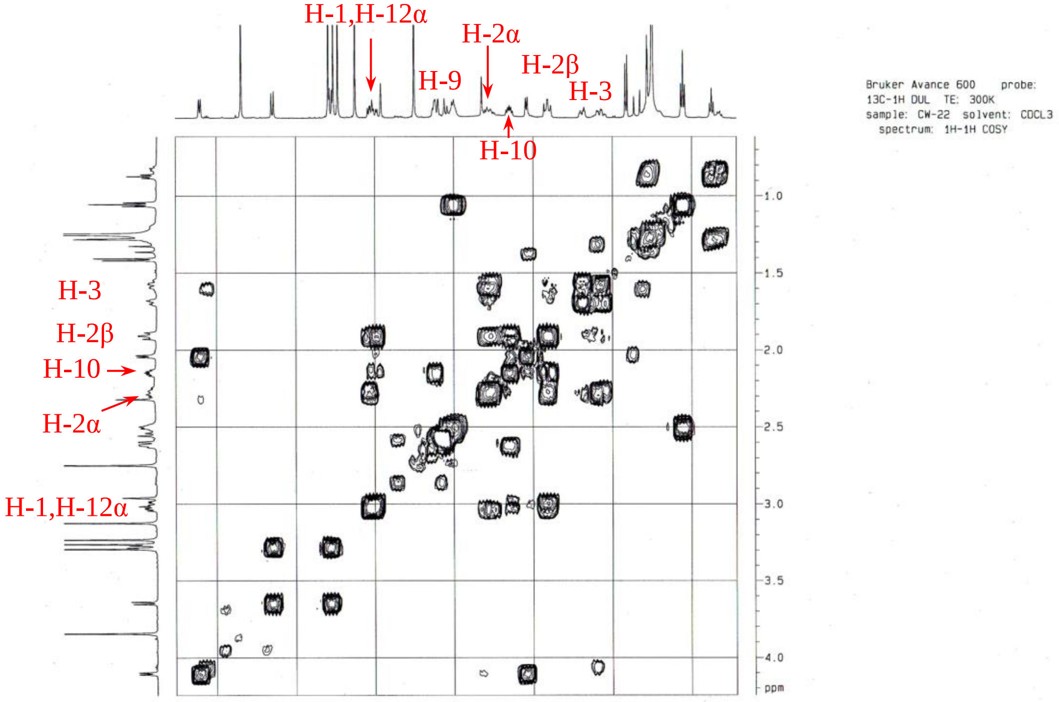

Supplementary Figure 23. Key ^{1}H-^{1}H COSY correlations of 16-demethoxy-$\Delta^{15(16)}$-8-*O*-methylcrassicauline A.

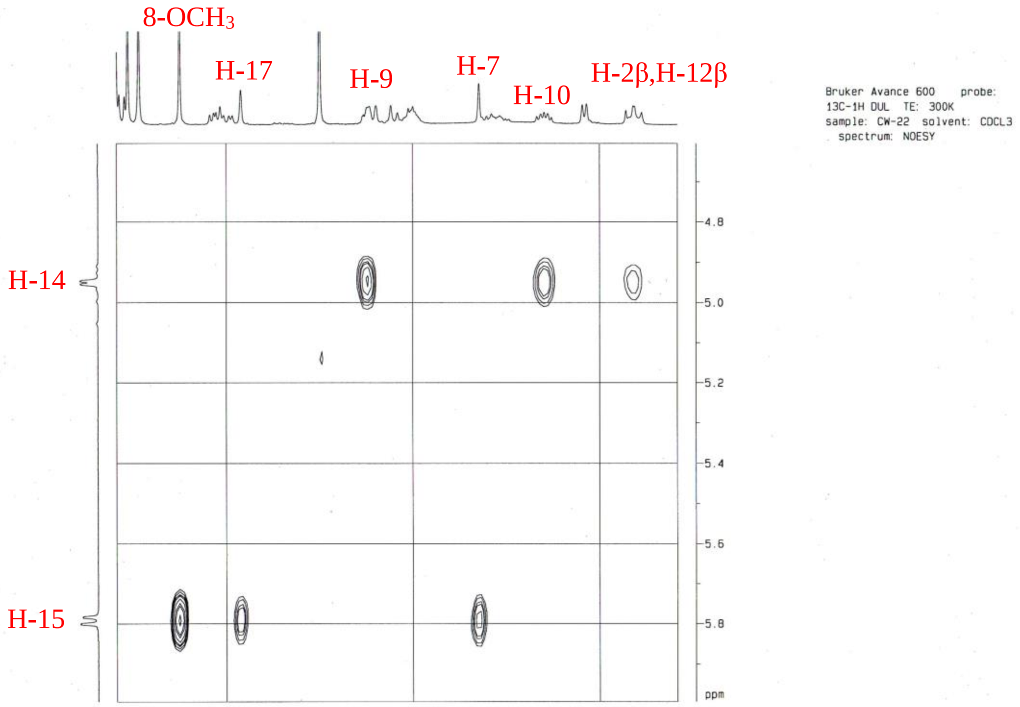

Supplementary Figure 24. Key NOESY correlations of 16-demethoxy-$\Delta^{15(16)}$-8-*O*-methylcrassicauline A (Detail 1).

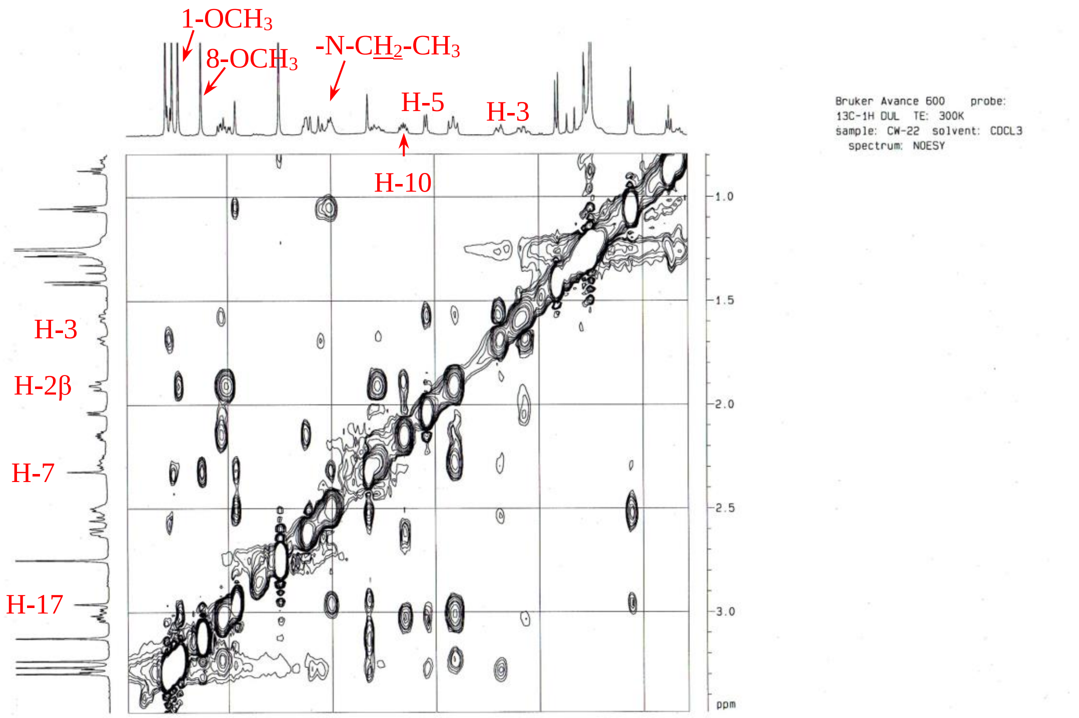

Supplementary Figure 25. Key NOESY correlations of 16-demethoxy-$\Delta^{15(16)}$-8-*O*-methylcrassicauline A (Detail 2).

Mass Spectrum SmartFormula Report

Analysis Info

Analysis Name	D:\Data\USER-2020\CW-22.d	Acquisition Date	7/30/2020 2:54:59 PM
Method	tune_low_NEW.m	Operator	Ma
Sample Name	CW-22	Instrument / Ser#	micrOTOF-Q II 10203
Comment			

Acquisition Parameter

Source Type	ESI	Ion Polarity	Positive	Set Nebulizer	0.8 Bar
Focus	Not active	Set Capillary	4500 V	Set Dry Heater	180 °C
Scan Begin	50 m/z	Set End Plate Offset	-500 V	Set Dry Gas	6.0 l/min
Scan End	1500 m/z	Set Collision Cell RF	150.0 Vpp	Set Divert Valve	Source

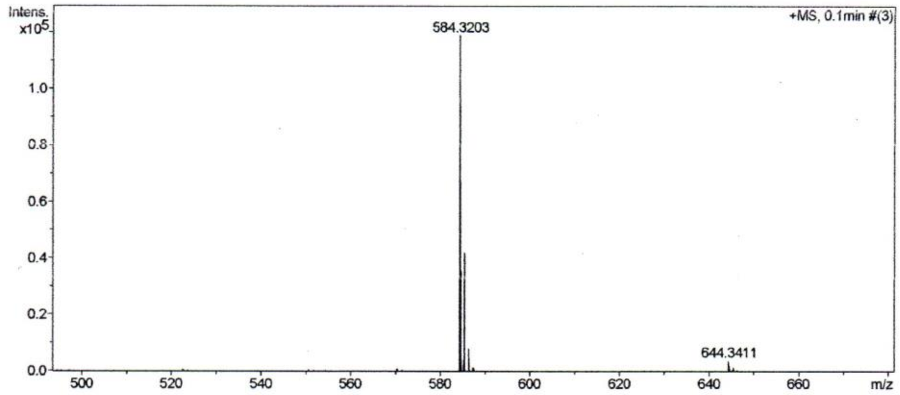

Meas. m/z	Formula	m/z	err [ppm]	mSigma	N-Rule	e⁻ Conf
584.3203						
	C 33 H 46 N O 8	584.3218	2.6	9.61	ok	even

Bruker Compass DataAnalysis 4.0 Page 1 of 1

Supplementary Figure 26. High-resolution ESI-MS of 16-demethoxy-$\Delta^{15(16)}$-8-*O*-methylcrassicauline A.

2.1.3 Structure elucidation of 16-demethoxy-Δ$^{15(16)}$-crassicauline A (compound 3)

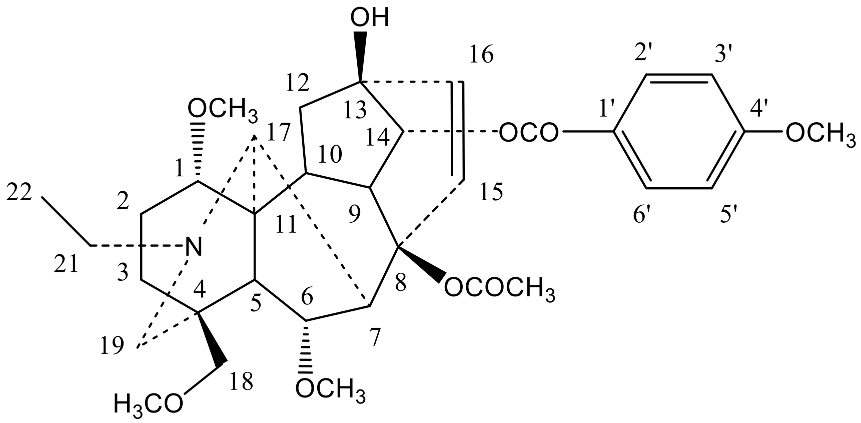

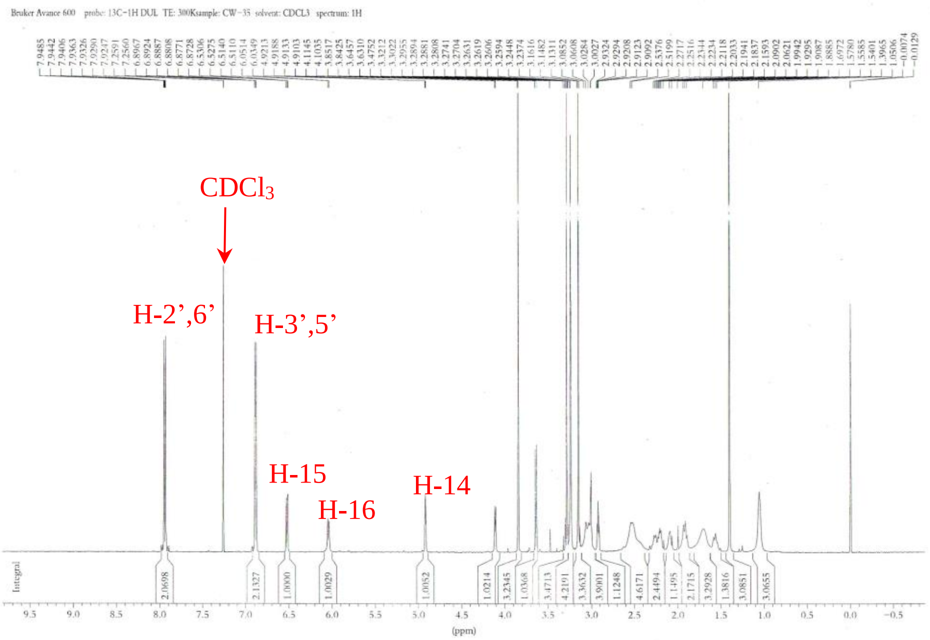

Supplementary Figure 27. ^{1}H NMR of 16-demethoxy-Δ$^{15(16)}$-crassicauline A.

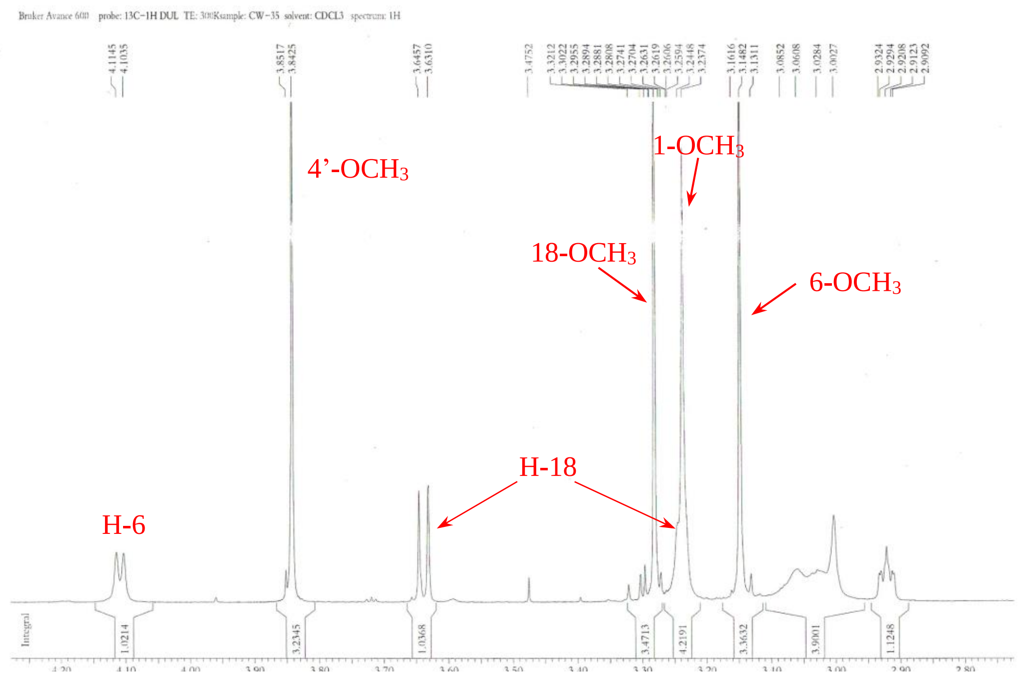

Supplementary Figure 28. ^{1}H NMR of 16-demethoxy-$\Delta^{15(16)}$-crassicauline A (Detail).

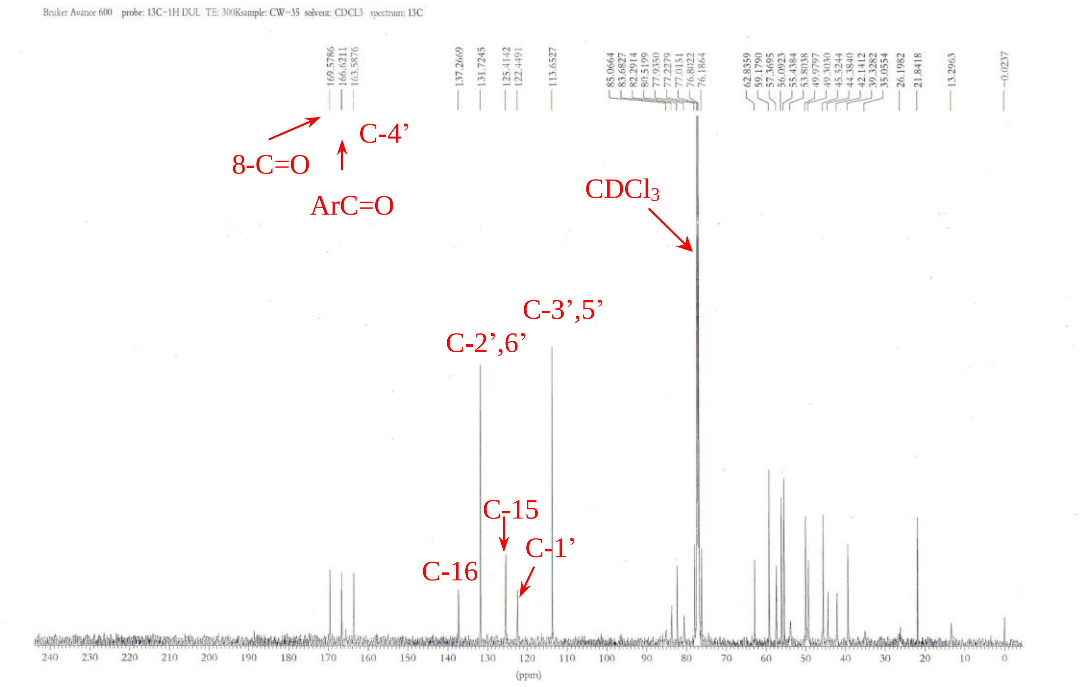

Supplementary Figure 29. ^{13}C NMR of 16-demethoxy-$\Delta^{15(16)}$-crassicauline A.

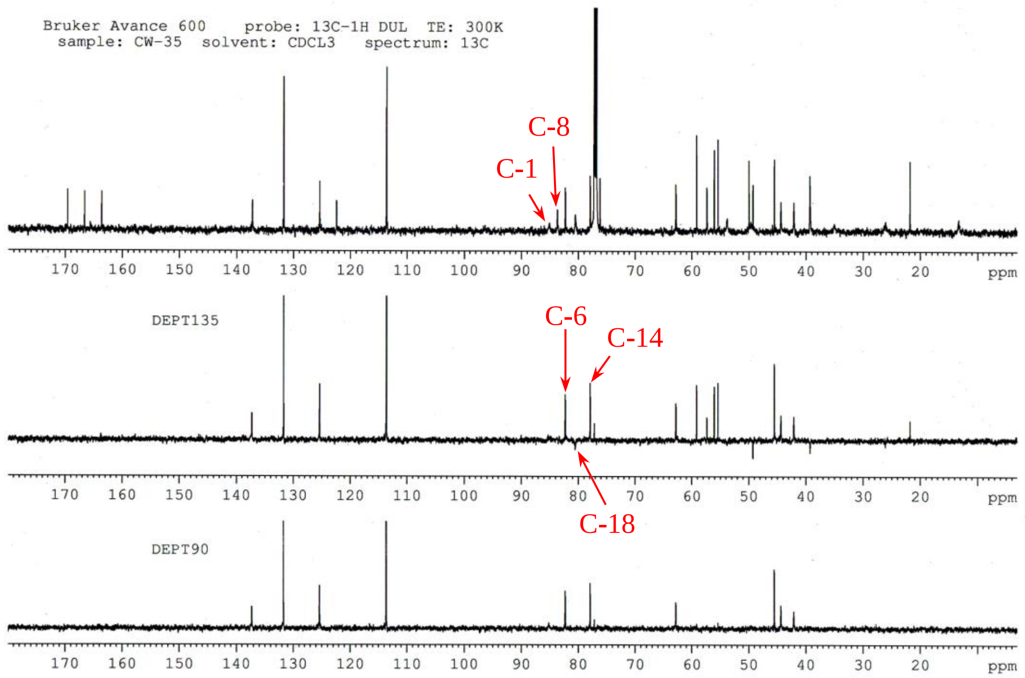

Supplementary Figure 30. DEPT of 16-demethoxy-$\Delta^{15(16)}$-crassicauline A.

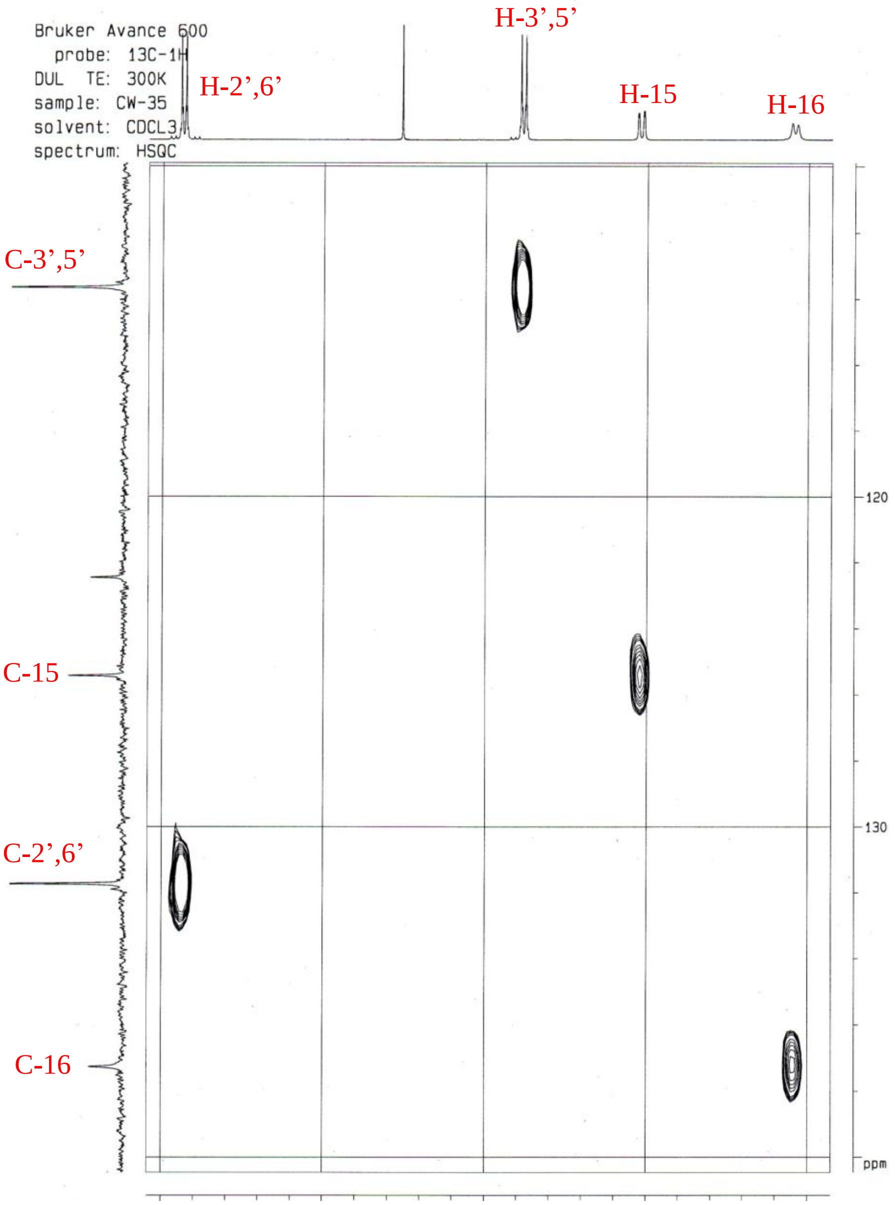

Supplementary Figure 31. Key HSQC correlations of 16-demethoxy-$\Delta^{15(16)}$-crassicauline A (Detail 1).

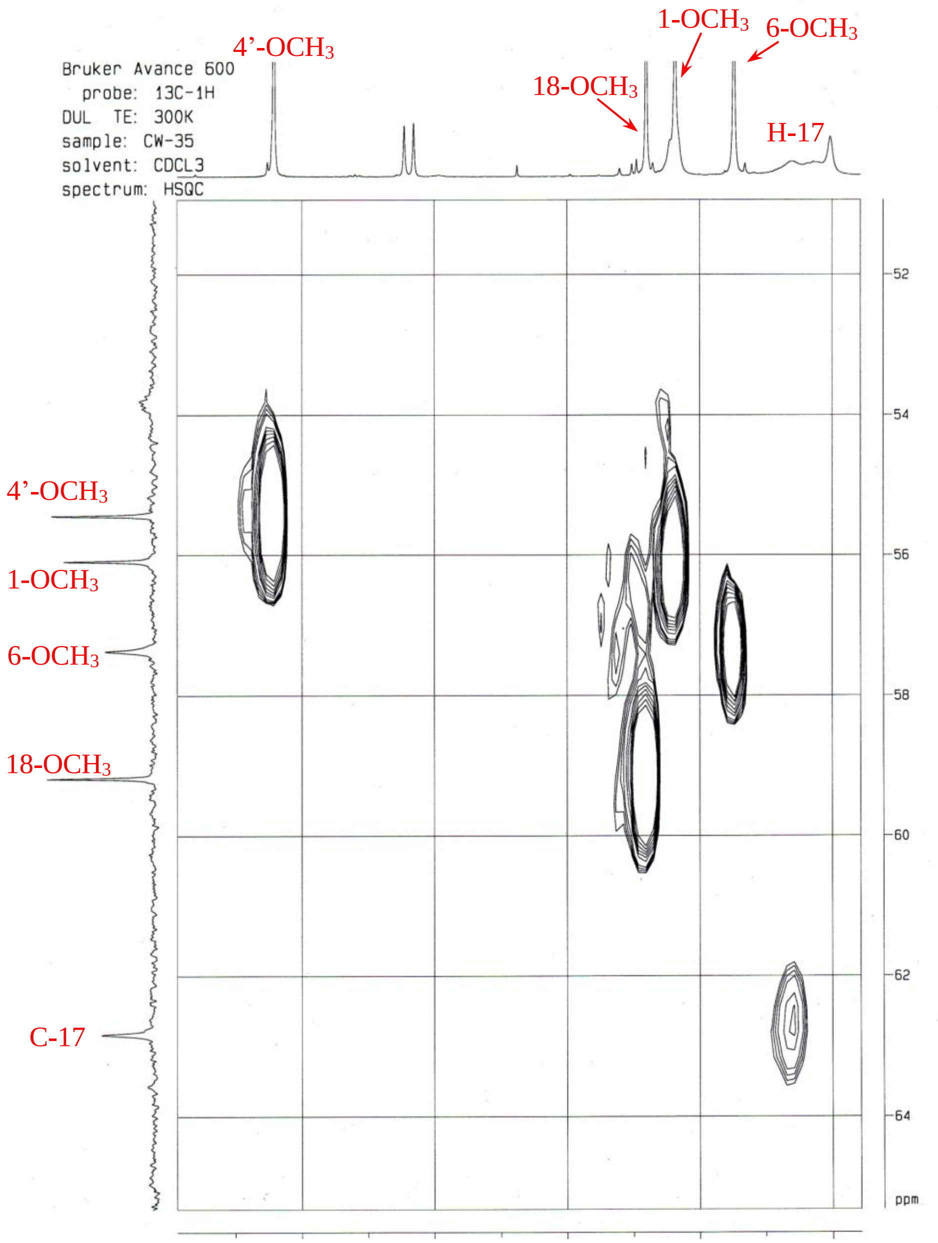

Supplementary Figure 32. Key HSQC correlations of 16-demethoxy-$\Delta^{15(16)}$-crassicauline A (Detail 2).

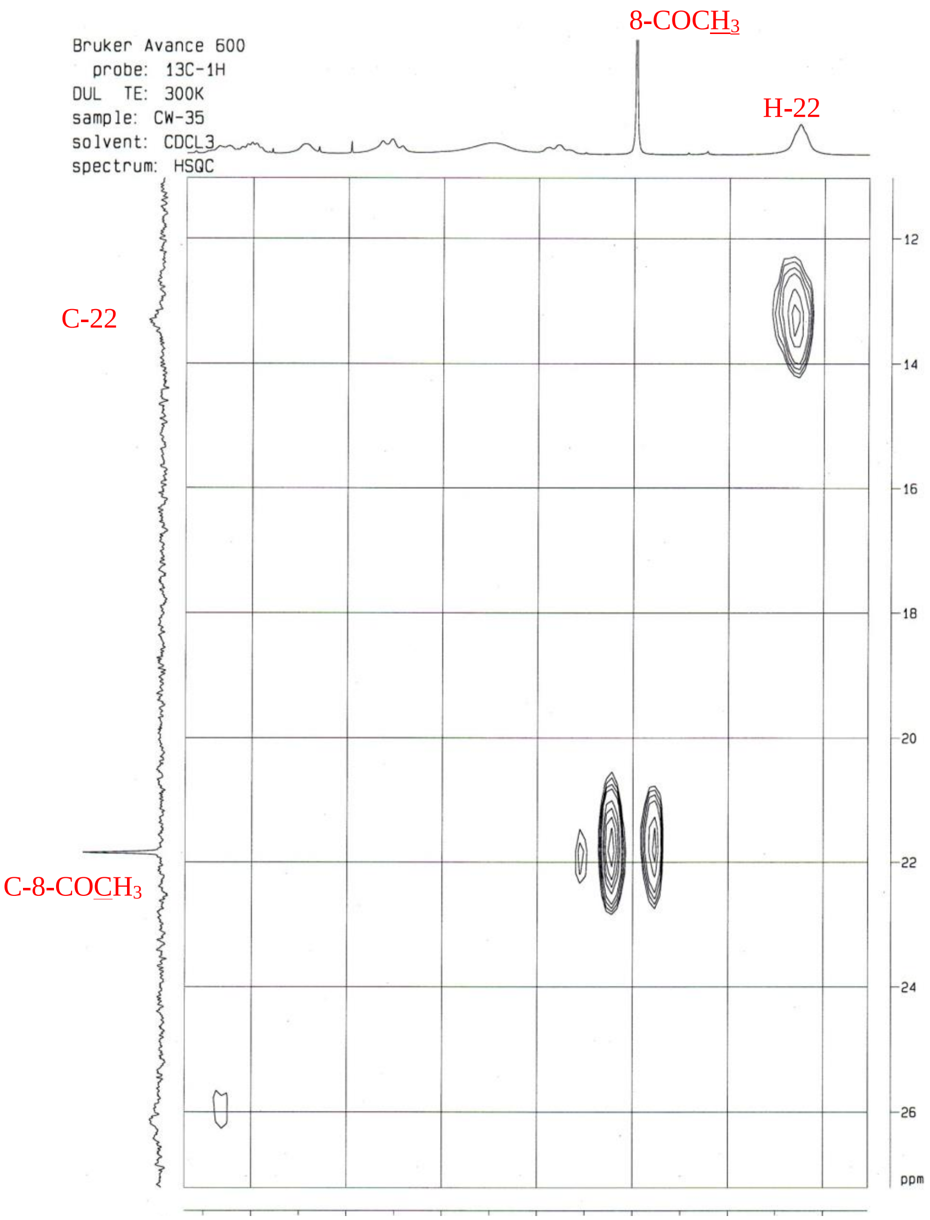

Supplementary Figure 33. Key HSQC correlations of 16-demethoxy-$\Delta^{15(16)}$-crassicauline A (Detail 3).

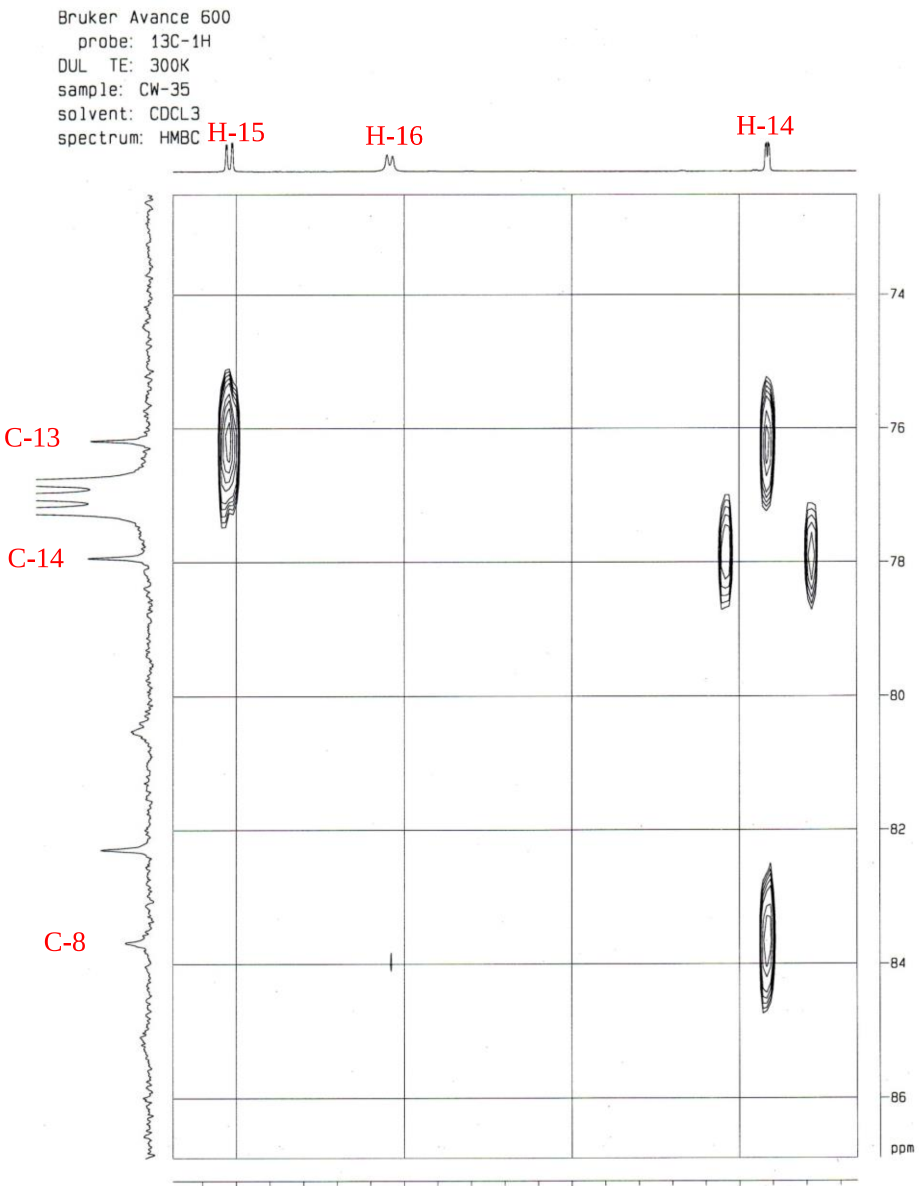

Supplementary Figure 34. Key HMBC correlations of 16-demethoxy-Δ$^{15(16)}$-crassicauline A (Detail 1).

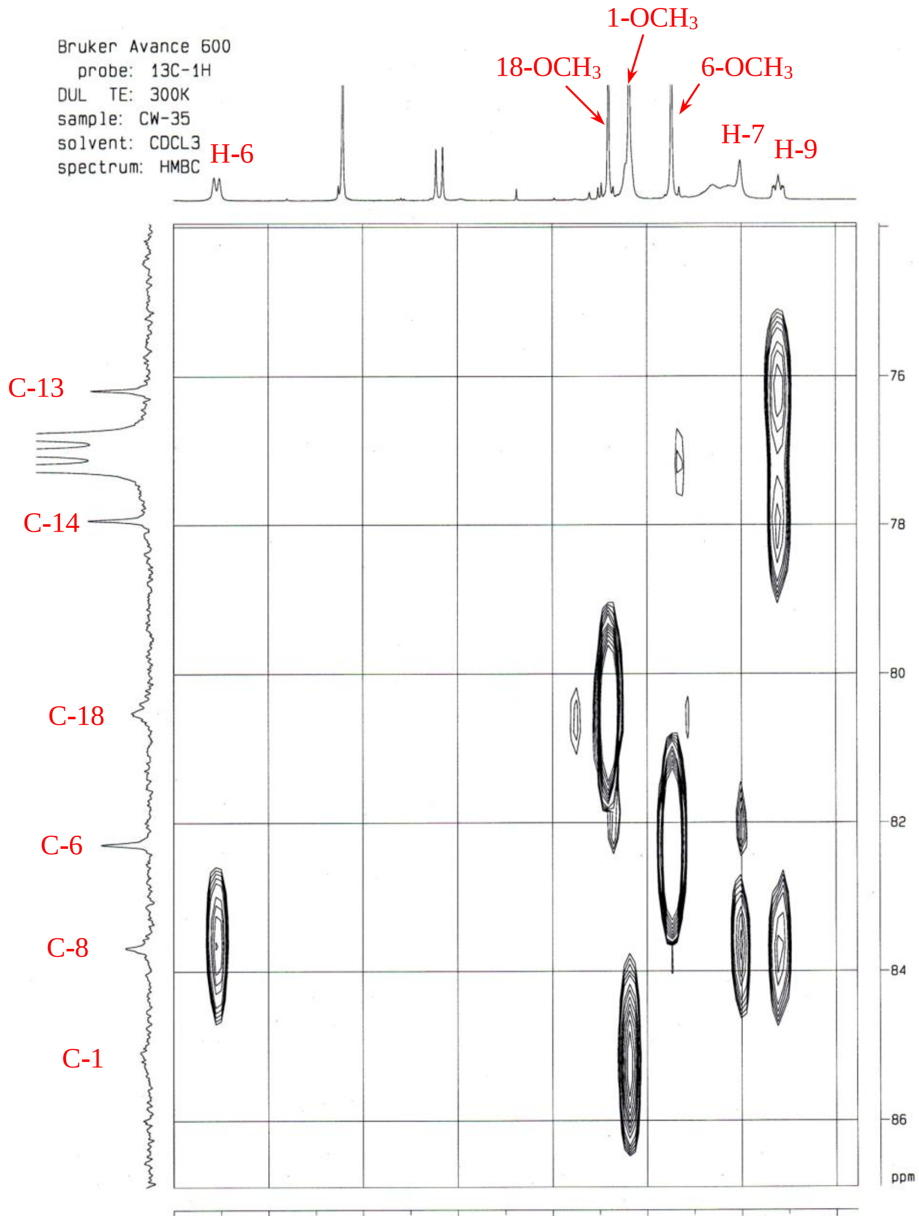

Supplementary Figure 35. Key HMBC correlations of 16-demethoxy-$\Delta^{15(16)}$-crassicauline A (Detail 2).

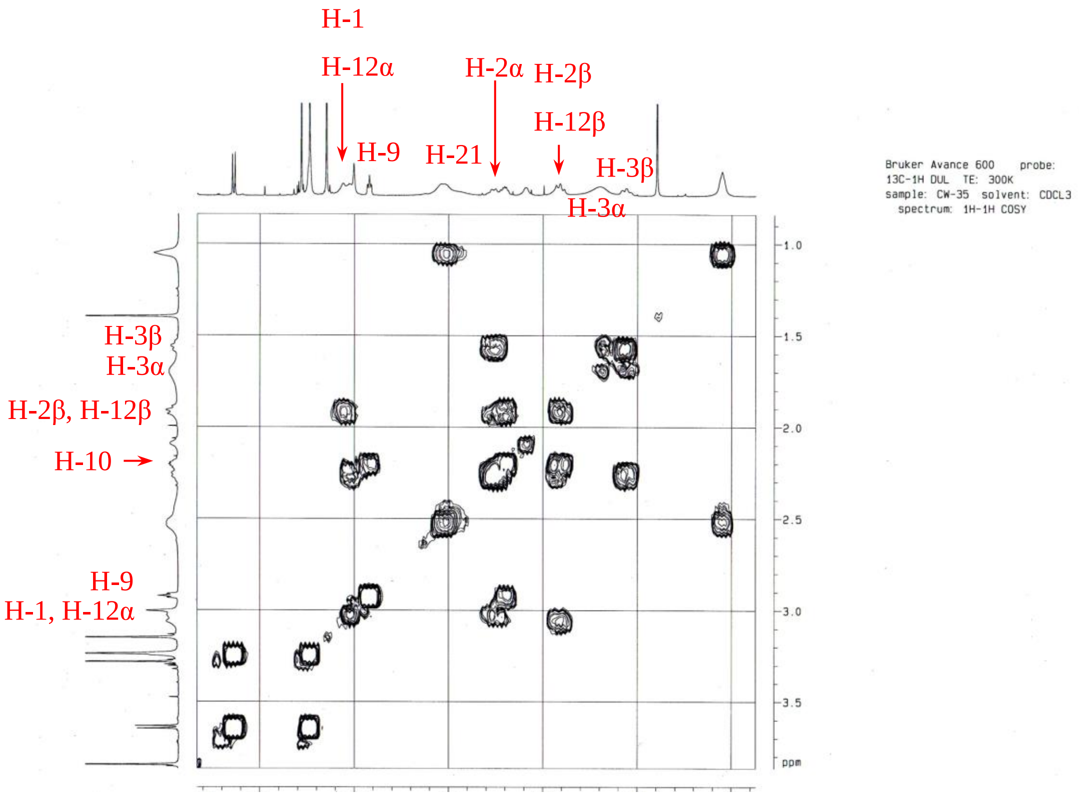

Supplementary Figure 36. Key ^{1}H-^{1}H COSY correlations of 16-demethoxy-$\Delta^{15(16)}$-crassicauline A.

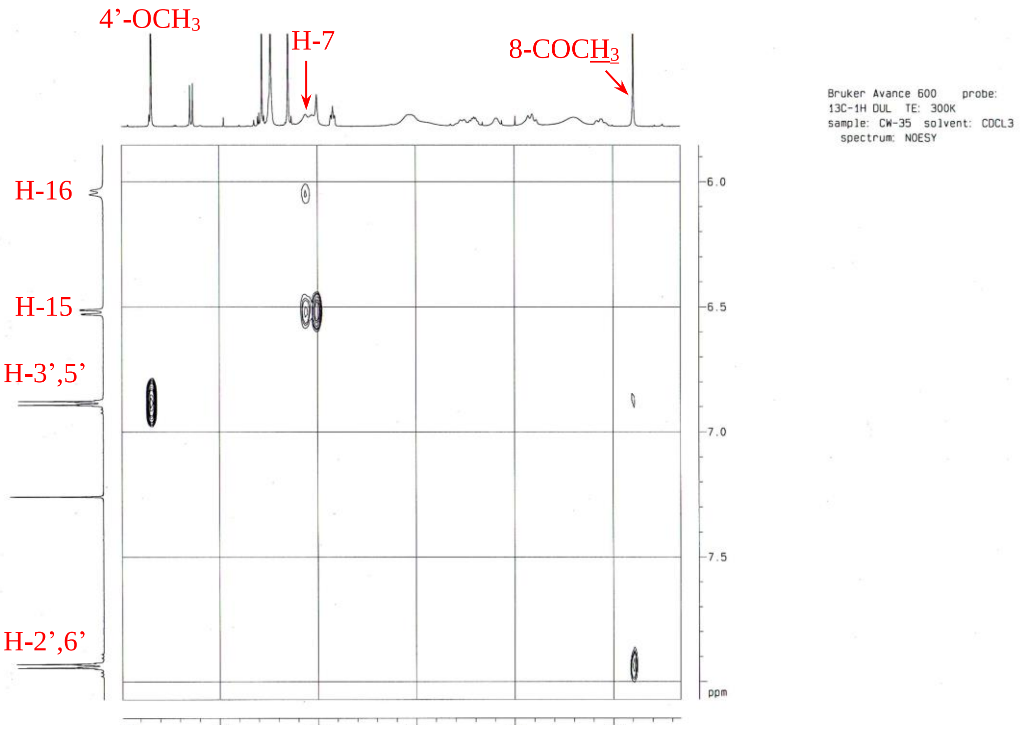

Supplementary Figure 37. Key NOESY correlations of 16-demethoxy-$\Delta^{15(16)}$-crassicauline A (Detail 1).

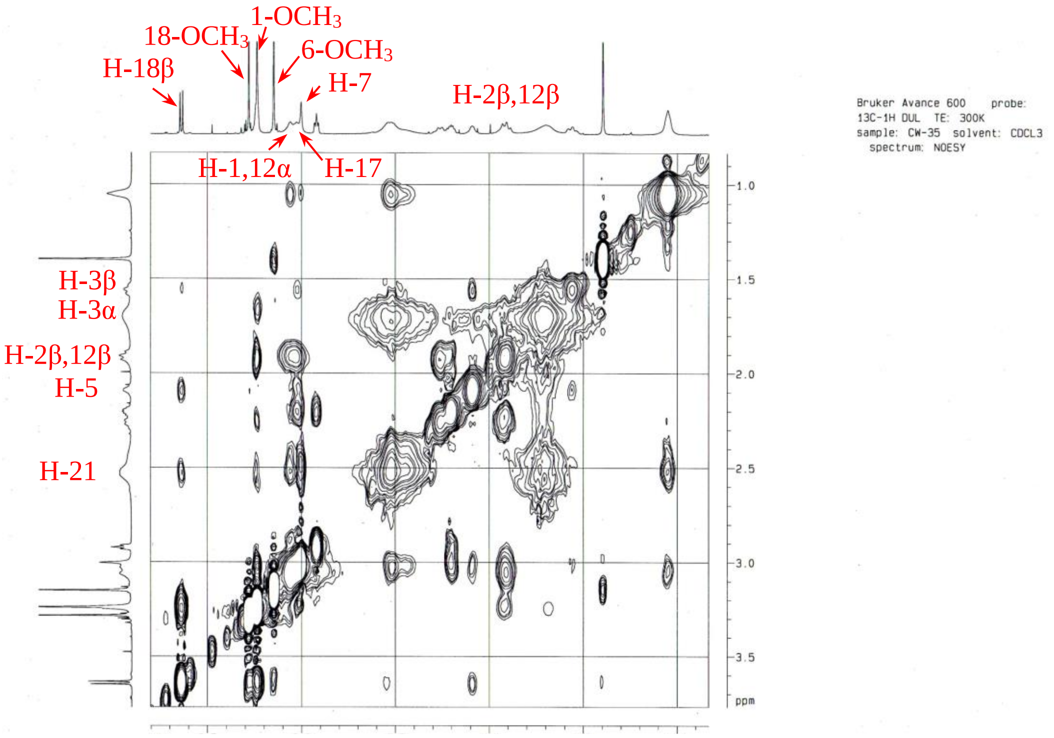

Supplementary Figure 38. Key NOESY correlations of 16-demethoxy-$\Delta^{15(16)}$-crassicauline A (Detail 2).

Mass Spectrum SmartFormula Report

Analysis Info

Analysis Name	D:\Data\USER-2020\CW-35.d	Acquisition Date	7/30/2020 2:56:45 PM
Method	tune_low_NEW.m	Operator	Ma
Sample Name	CW-35	Instrument / Ser#	micrOTOF-Q II 10203
Comment			

Acquisition Parameter

Source Type	ESI	Ion Polarity	Positive	Set Nebulizer	0.8 Bar
Focus	Not active	Set Capillary	4500 V	Set Dry Heater	180 °C
Scan Begin	50 m/z	Set End Plate Offset	-500 V	Set Dry Gas	6.0 l/min
Scan End	1500 m/z	Set Collision Cell RF	150.0 Vpp	Set Divert Valve	Source

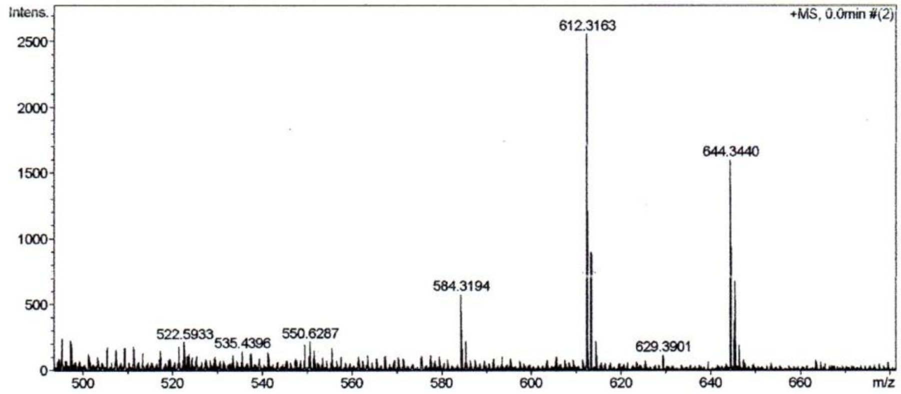

Meas. m/z	Formula	m/z	err [ppm]	mSigma	N-Rule	e⁻ Conf
612.3163						
	C 30 H 171 O 5	612.3121	-6.8	11.40	ok	even

Bruker Compass DataAnalysis 4.0 Page 1 of 1

Supplementary Figure 39. High-resolution ESI-MS of 16-demethoxy-$\Delta^{15(16)}$-crassicauline A.

2.1.4 Structure elucidation of pyrocrassicauline A (compound 4)

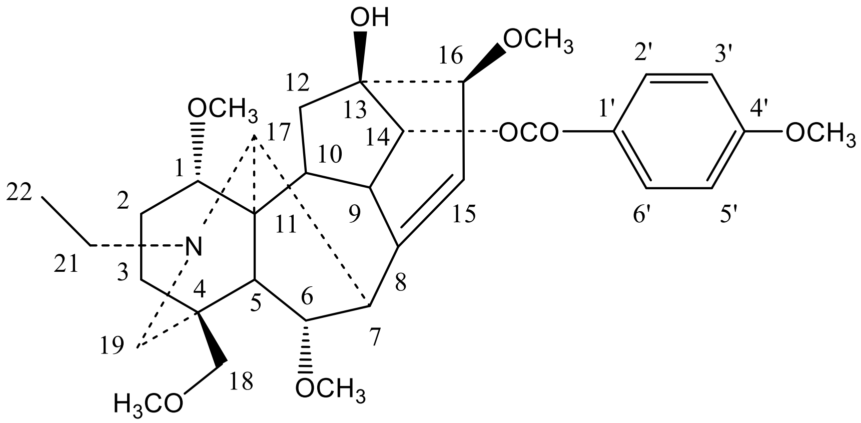

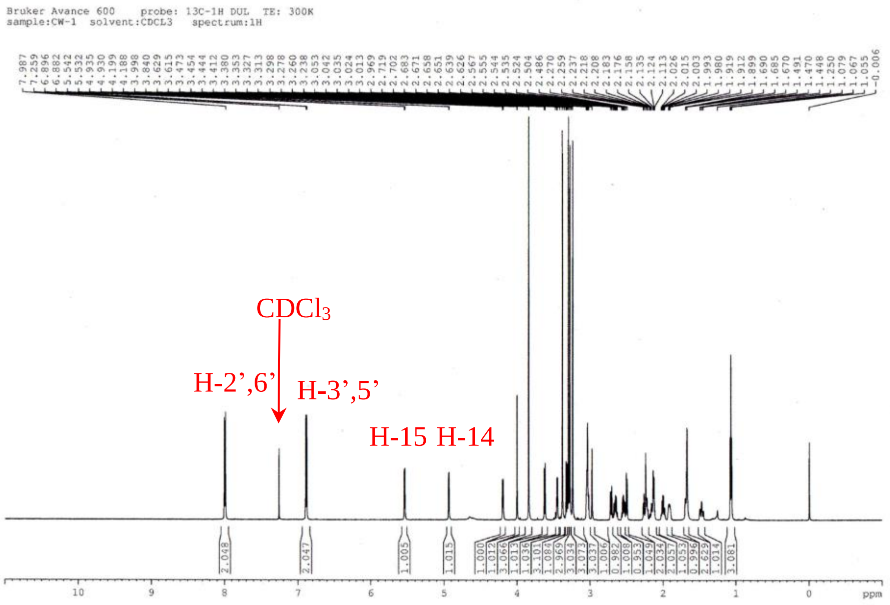

Supplementary Figure 40. ^{1}H NMR of pyrocrassicauline A.

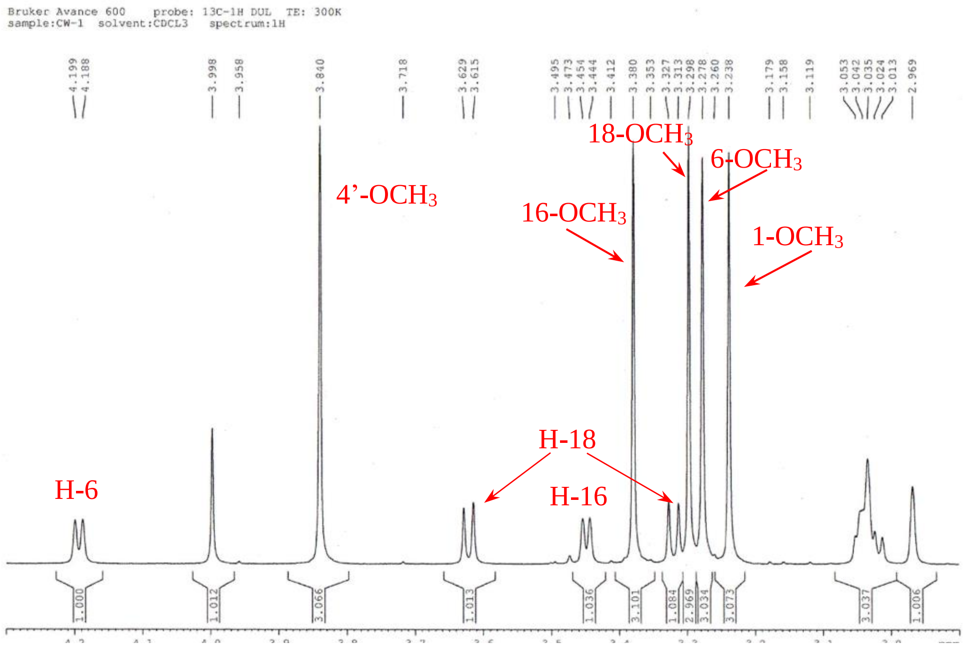

Supplementary Figure 41. ^{1}H NMR of pyrocrassicauline A (Detail).

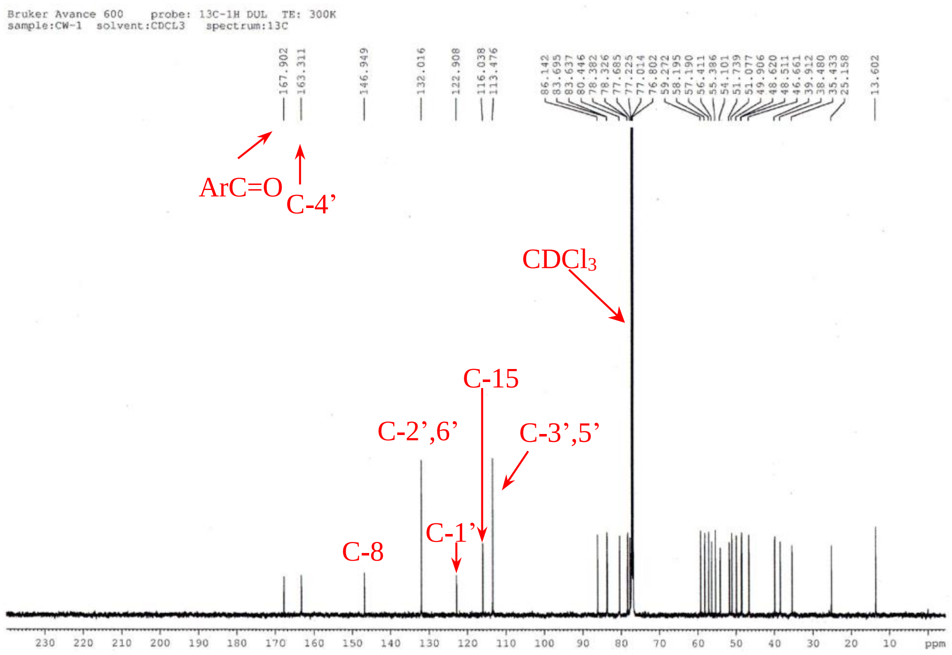

Supplementary Figure 42. ^{13}C NMR of pyrocrassicauline A.

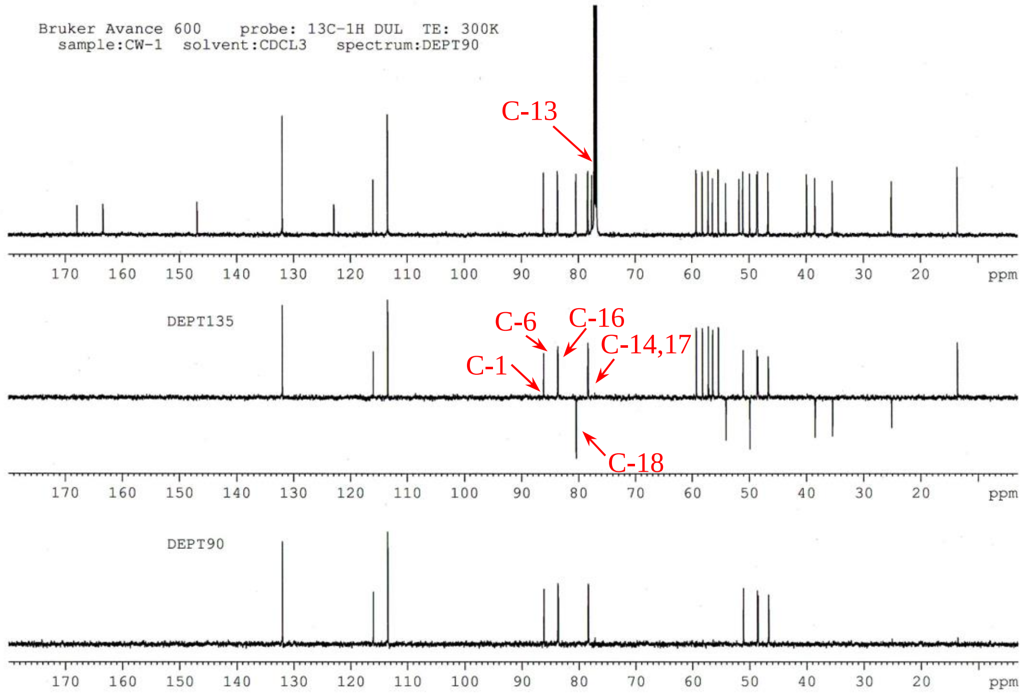

Supplementary Figure 43. DEPT of pyrocrassicauline A.

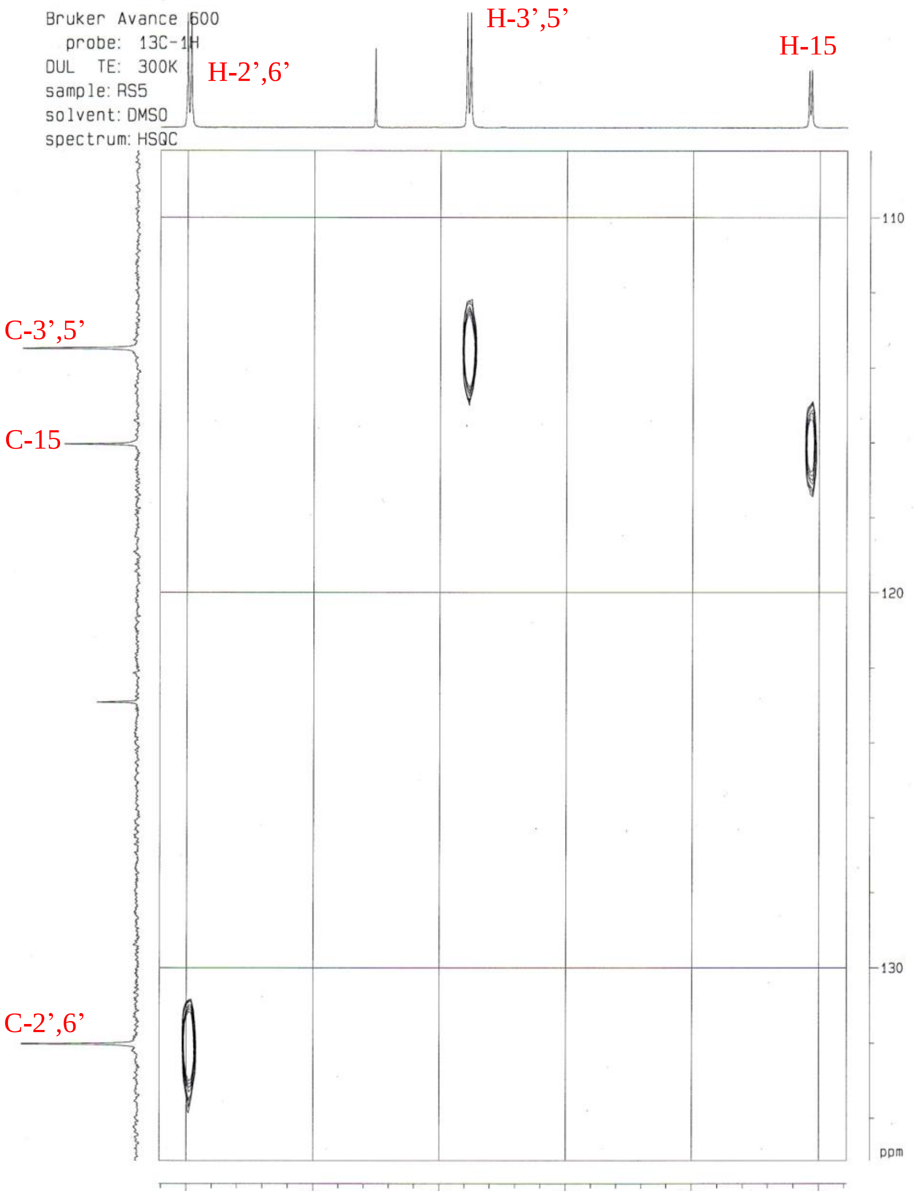

Supplementary Figure 44. Key HSQC correlations of pyrocrassicauline A (Detail 1).

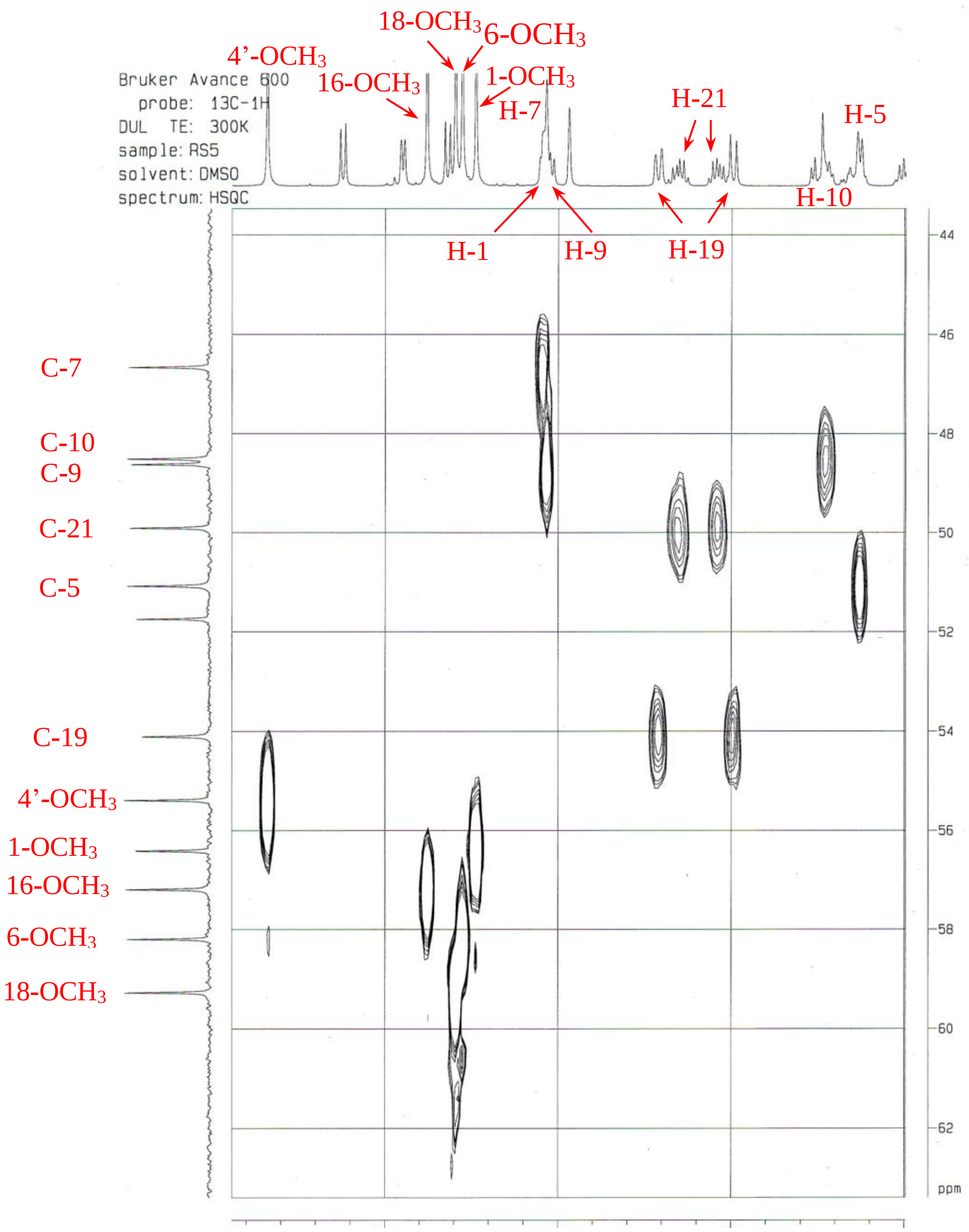

Supplementary Figure 45. Key HSQC correlations of pyrocrassicauline A (Detail 2).

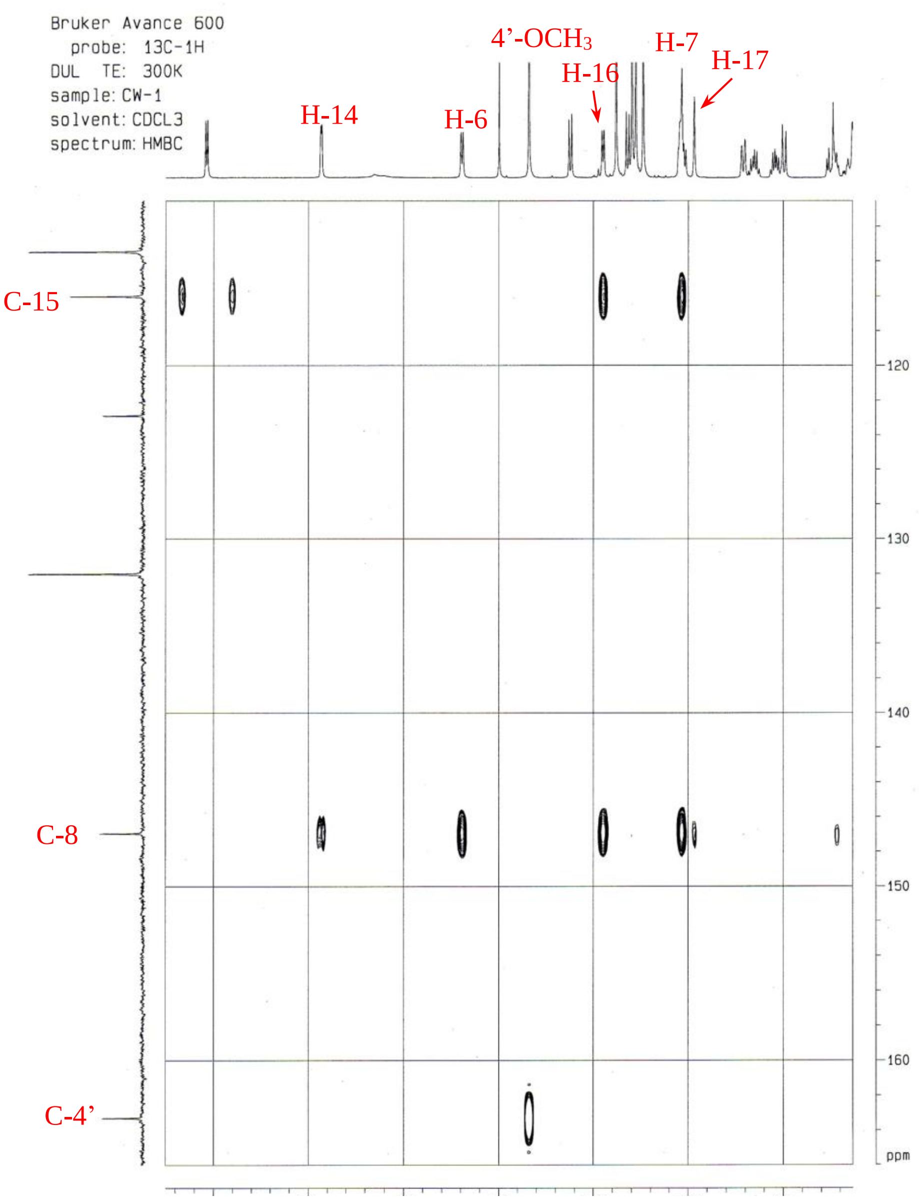

Supplementary Figure 46. Key HMBC correlations of pyrocrassicauline A (Detail 1).

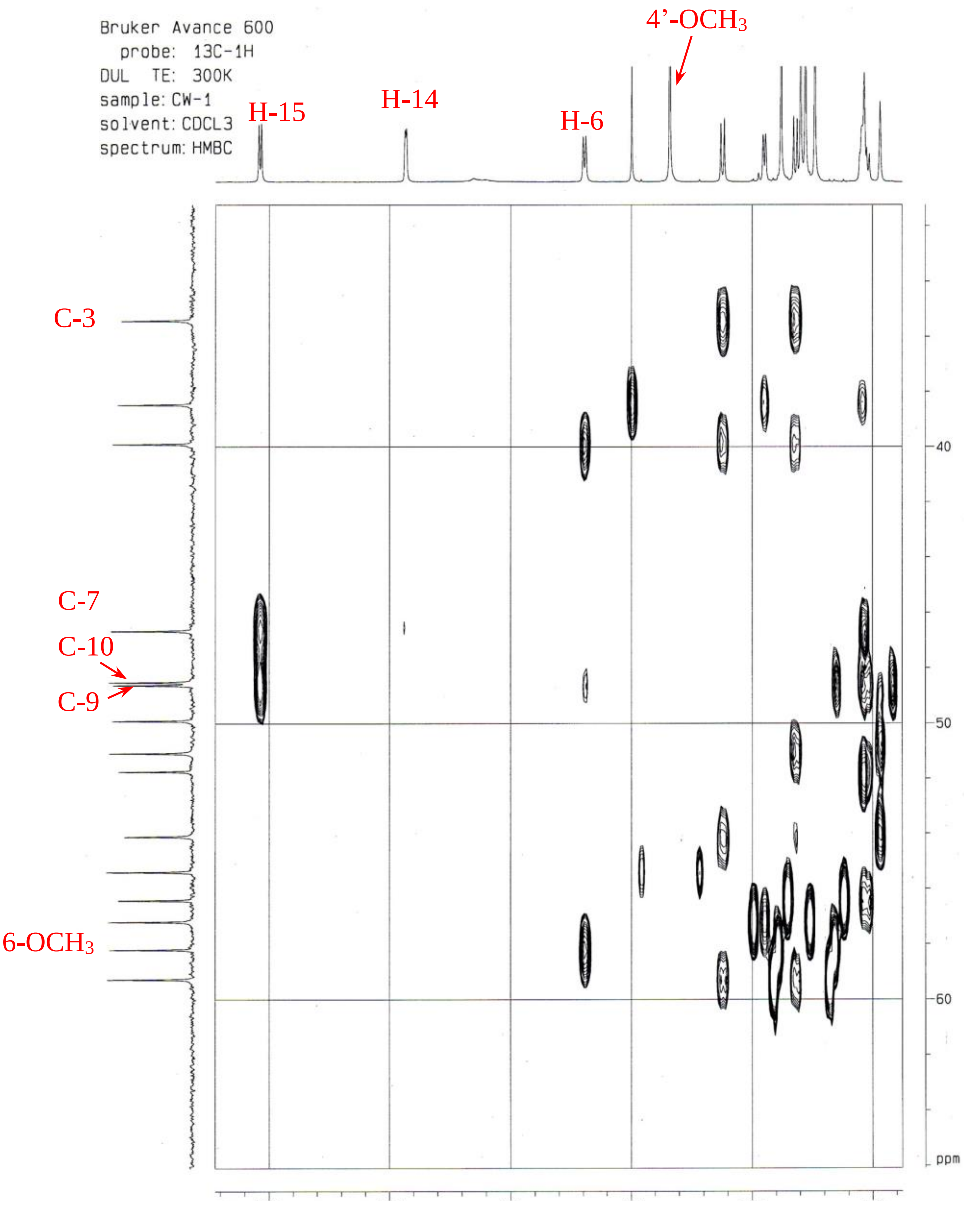

Supplementary Figure 47. Key HMBC correlations of pyrocrassicauline A (Detail 2).

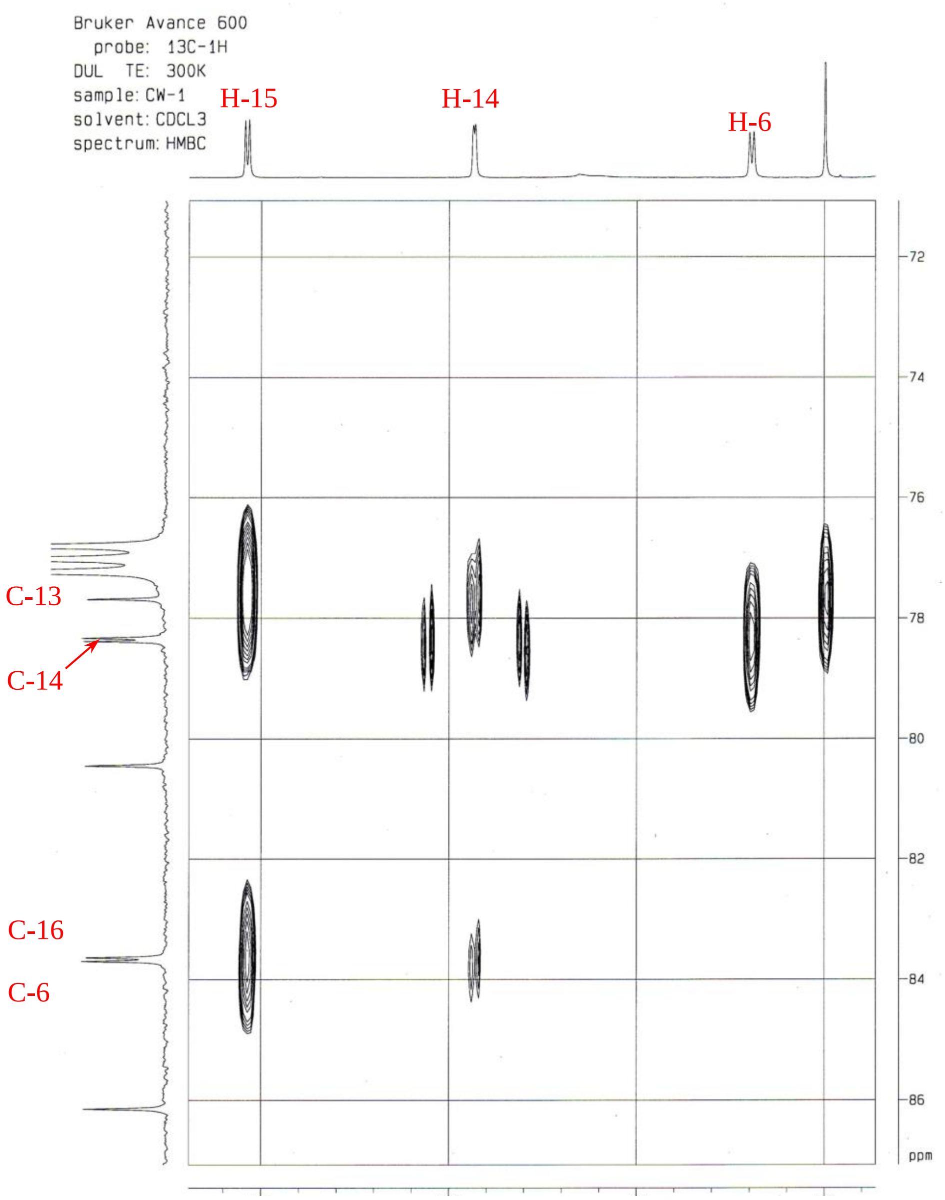

Supplementary Figure 48. Key HMBC correlations of pyrocrassicauline A (Detail 3).

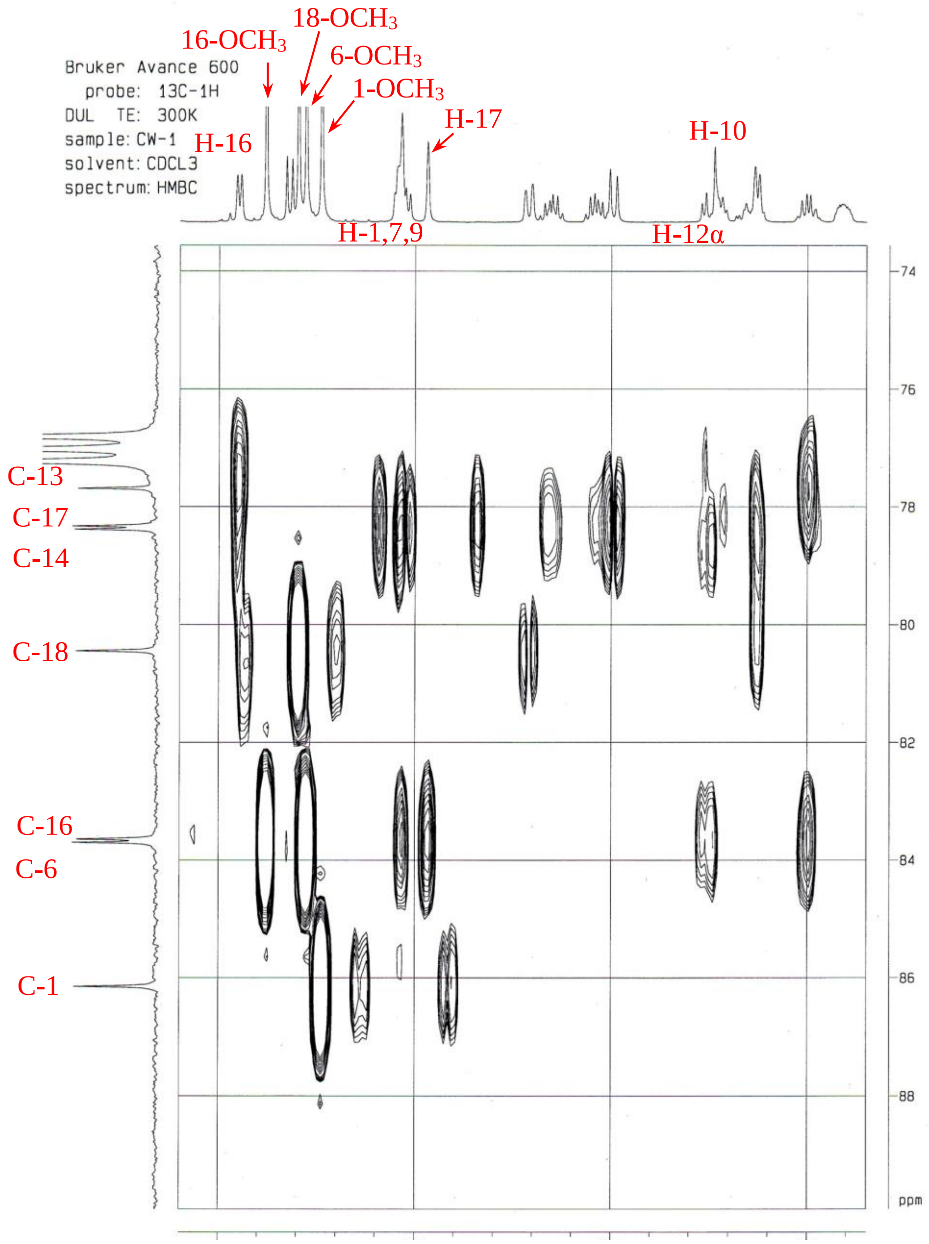

Supplementary Figure 49. Key HMBC correlations of pyrocrassicauline A (Detail 4).

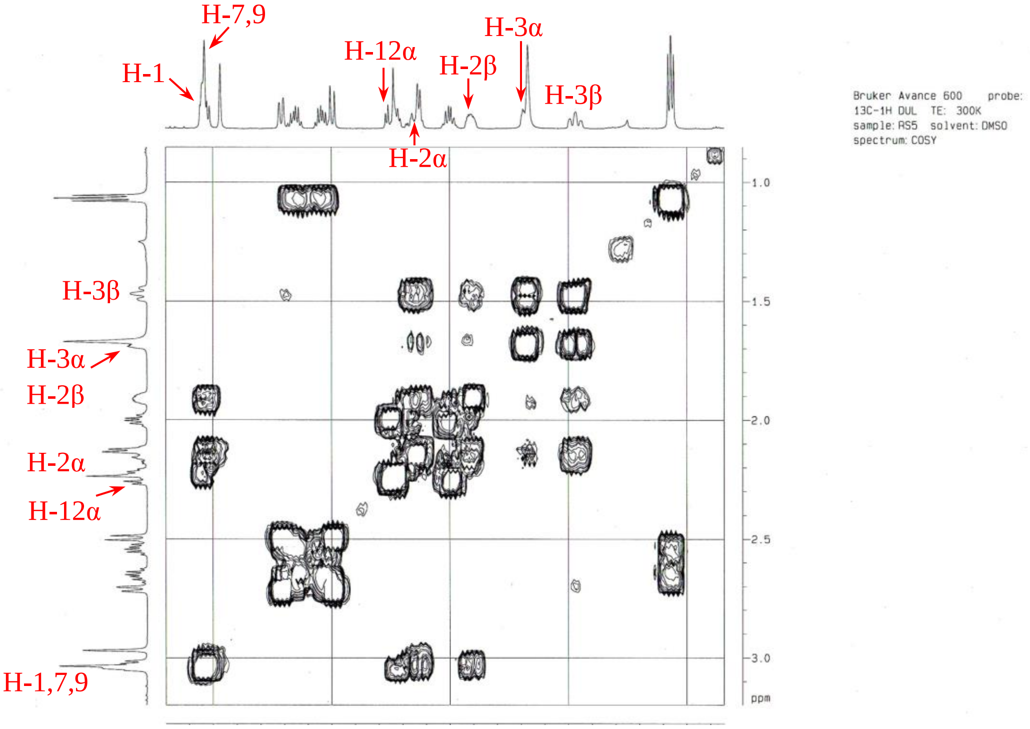

Supplementary Figure 50. Key ^{1}H-^{1}H COSY correlations of pyrocrassicauline A.

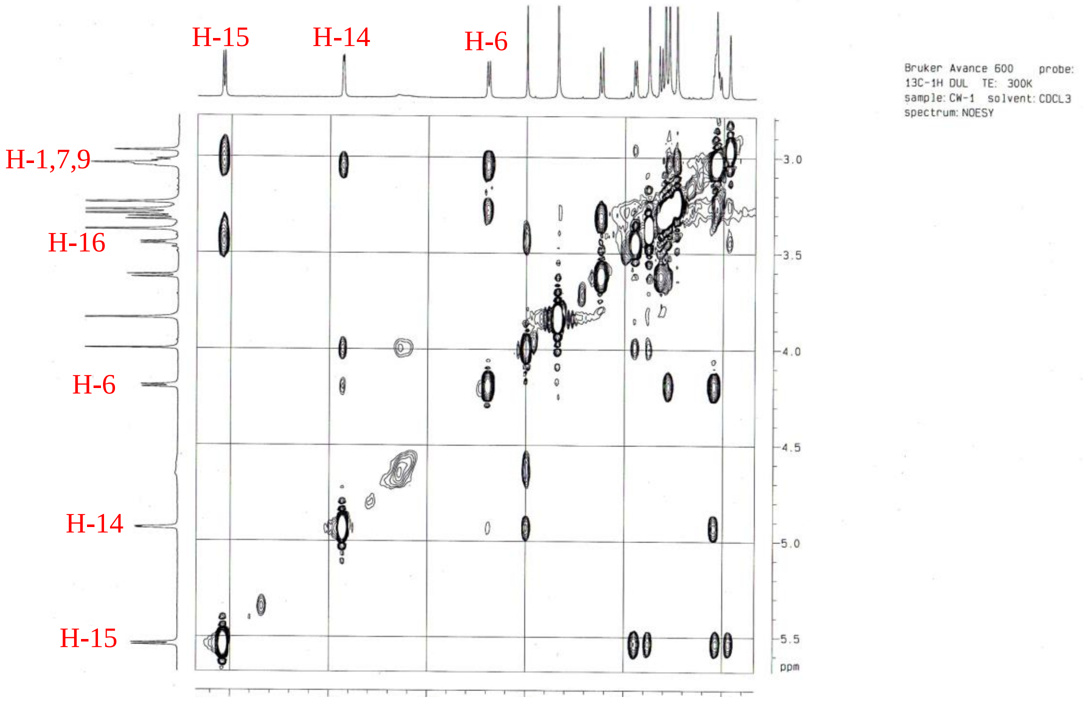

Supplementary Figure 51. Key NOESY correlations of pyrocrassicauline A (Detail 1).

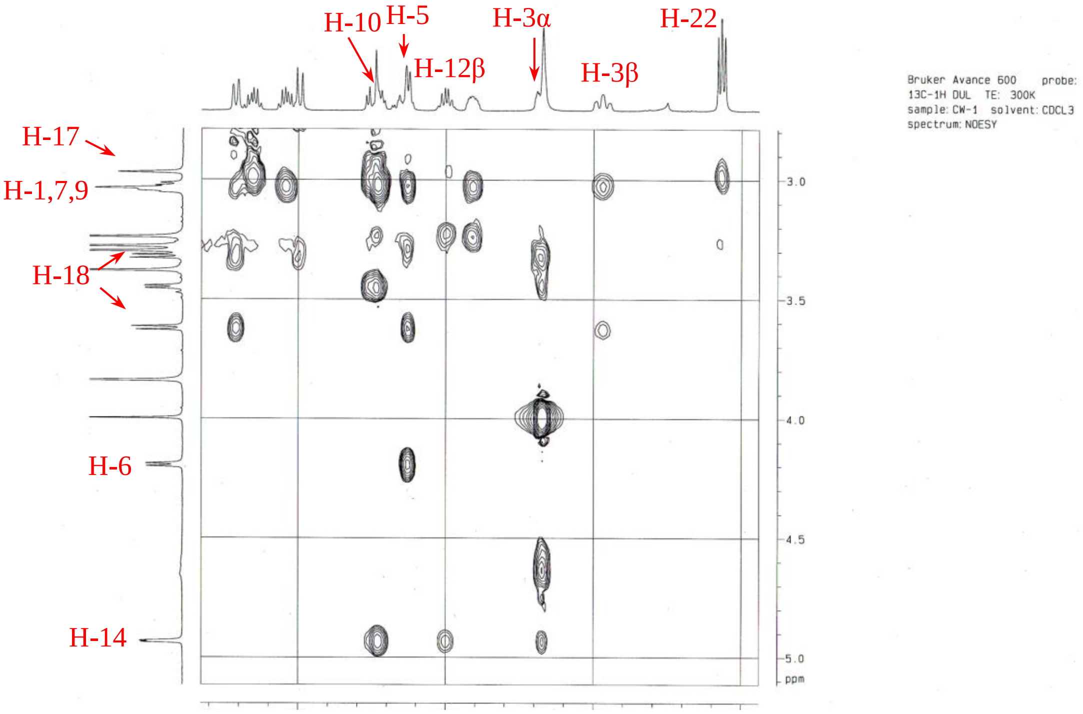

Supplementary Figure 52. Key NOESY correlations of pyrocrassicauline A (Detail 2).

Mass Spectrum SmartFormula Report

Analysis Info

Analysis Name	D:\Data\USER-2019\CW-1.d	Acquisition Date	11/26/2019 2:22:46 PM
Method	tune_low_NEW.m	Operator	Ma
Sample Name	CW-1	Instrument / Ser#	micrOTOF-Q II 10203
Comment			

Acquisition Parameter

Source Type	ESI	Ion Polarity	Positive	Set Nebulizer	0.8 Bar
Focus	Not active	Set Capillary	4500 V	Set Dry Heater	180 °C
Scan Begin	50 m/z	Set End Plate Offset	-500 V	Set Dry Gas	6.0 l/min
Scan End	1500 m/z	Set Collision Cell RF	150.0 Vpp	Set Divert Valve	Source

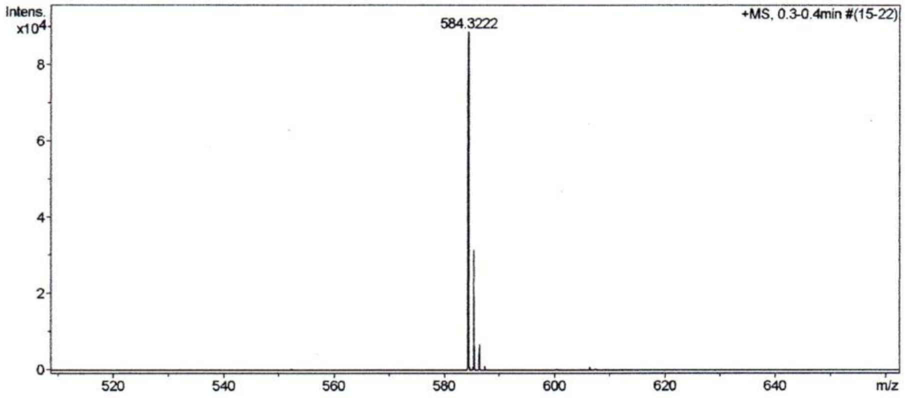

Meas. m/z	Formula	m/z	err [ppm]	mSigma	N-Rule	e⁻ Conf
584.3222						
	C 31 H 47 N Na O 8	584.3194	-4.8	6.05	ok	even
	C 33 H 46 N O 8	584.3218	-0.7	6.17	ok	even

Bruker Compass DataAnalysis 4.0 Page 1 of 1

Supplementary Figure 53. High-resolution ESI-MS of pyrocrassicauline A.

2.1.5 Representative ECGs of SD rats

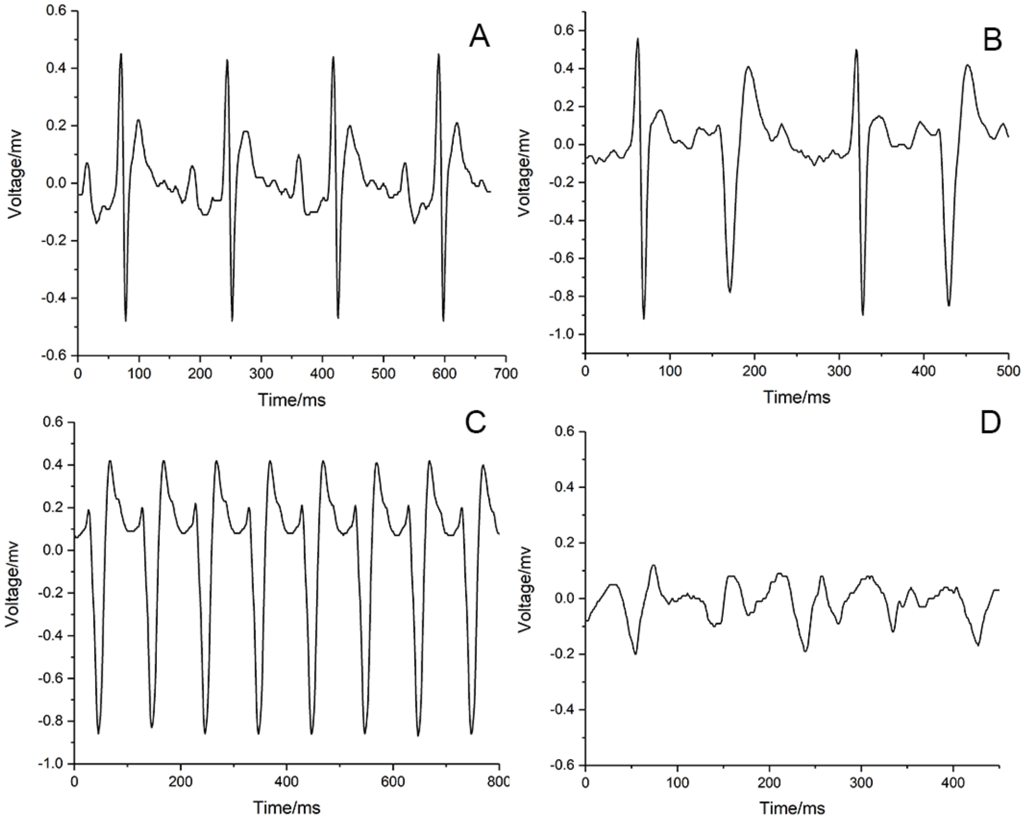

Supplementary Figure 54. Representative ECGs of SD rats. A: normal electrocardiogram, B: ventricular premature beat, C: ventricular tachycardia, D: ventricular fibrillation.

2.2 Supplementary Table

Supplementary Table 1. ^{1}H (600 MHz) and ^{13}C (150 MHz) NMR data for compound **4** ($CDCl_3$)

Position	δ_H (*J* in Hz)	δ_C, type	HMBC	NOESY	^{1}H-^{1}H COSY
1	3.05 m	86.1, CH	C-10, C-17, 1-OCH_3	H-3β, H-10, 1-OCH_3	H-2α, β
2α	2.18 m	25.2, CH_2	C-3, C-4, C-11	——	H-1, H-2β, H-3α, β
2β	1.92 m		C-3, C-4, C-11	——	H-1, H-2α, H-3α, β
3α	1.69 m	35.4, CH_2	——	H-18α	H-2α, β, H-3β
3β	1.47 m		——	H-1, H-5	H-2α, β, H-3α
4	——	39.9, C	——	——	——
5	2.12 d (6.6)	51.1, CH	C-10, C-17, C-18, C-19	H-3β, H-18α, β	H-6
6	4.19 d (6.6)	83.7, CH	C-4, C-8, C-17, 6-OCH_3	H-9, 6-OCH_3	H-5, H-7
7	3.04 s	46.7, CH	C-11, C-15	H-15, 6-OCH_3	H-6, H-17
8	——	146.9, C	——	——	——
9	3.01 m	48.6, CH	C-11, C-12	H-6	H-10, H-14
10	2.22 m	48.5, CH	C-8, C-17	H-1, H-14	H-9, H-12α, β
11	——	51.7, C	——	——	——
12α	2.27 m	38.5, CH_2	C-9, C-11, C-14, C-16	——	H-10, H-12β
12β	2.00 m		C-9, C-16	H-14	H-10, H-12α
13	——	77.7, C	——	——	——
14	4.93 d (3.0)	78.4, CH	C-8, C-10, C-16	H-10, H-12β	H-9
15	5.53 d (6.0)	116.0, CH	C-7, C-9, C-13	H-7, H-17	H-16
16	4.19 d (6.0)	83.6, CH	C-8, C-12, 16-OCH_3	H-17	H-15
17	2.97 s	78.3, CH	C-5, C-6, C-8, C-10, C-19	H-15, H-16, H-21, H-22	——
18α	3.33 d (8.4)	80.4, CH_2	C-3, C-5, C-19, 18-OCH_3	H-3α, H-5, H-19α, β, 18-OCH_3	H-18β
18β	3.63 d (8.4)		C-3, C-19, 18-OCH_3	H-5, H-19β, 18-OCH_3	H-18α
19α	2.50 d (10.2)	54.1, CH_2	C-3, C-5, C-17	H-18α	H-19β
19β	2.72 d (10.2)		C-3, C-18, C-21	H-18α, β	H-19α
21α	2.54 m	49.9, CH_2	C-17, C-19	H-17	H-22
21β	2.66 m		C-17, C-19	H-17	H-22
22	1.07 t (7.2)	13.6, CH_3	——	H-17	H-21α, β
1-OCH_3	3.24 s	56.4, CH_3	C-1	H-1	——
6-OCH_3	3.28 s	58.2, CH_3	C-6	H-6, H-7	——
16-OCH_3	3.38 s	57.2, CH_3	C-16	H-16, H-2′, 6′	——
18-OCH_3	3.30 s	59.3, CH_3	C-18	H-18α, β	——
ArC=O	——	167.9, C	——	——	——
1′	——	122.9, C	——	——	——
2′, 6′	7.99 d (8.4)	132.0, CH	C-4′, ArC=O	16-OCH_3	H-3′, 5′
3′, 5′	6.88 d (8.4)	113.5, CH	C-1′	4′-OCH_3	H-2′, 6′
4′	——	163.3, C	——	——	——
4′-OCH_3	3.84 s	55.4, CH_3	C-4′	H-3′, 5′	——
